# Iron supplementation is sufficient to rescue skeletal muscle mass and function in cancer cachexia

**DOI:** 10.15252/embr.202153746

**Published:** 2022-02-24

**Authors:** Elisabeth Wyart, Myriam Y Hsu, Roberta Sartori, Erica Mina, Valentina Rausch, Elisa S Pierobon, Mariarosa Mezzanotte, Camilla Pezzini, Laure B Bindels, Andrea Lauria, Fabio Penna, Emilio Hirsch, Miriam Martini, Massimiliano Mazzone, Antonella Roetto, Simonetta Geninatti Crich, Hans Prenen, Marco Sandri, Alessio Menga, Paolo E Porporato

**Affiliations:** ^1^ Department of Molecular Biotechnology and Health Sciences Molecular Biotechnology Center University of Torino Turin Italy; ^2^ Department of Biomedical Sciences University of Padova Padova Italy; ^3^ Department of Surgical, Oncological and Gastroenterological Sciences Padova University Hospital Padova Italy; ^4^ Department of Clinical and Biological Sciences University of Torino Turin Italy; ^5^ Metabolism and Nutrition Research Group Louvain Drug Research Institute Université catholique de Louvain (UCLouvain) Brussels Belgium; ^6^ Department of Life Sciences and System Biology University of Torino Turin Italy; ^7^ Laboratory of Tumor Inflammation and Angiogenesis Center for Cancer Biology (CCB) Vlaams Instituut voor Biotechnologie (VIB) Leuven Belgium; ^8^ Laboratory of Tumor Inflammation and Angiogenesis Department of Oncology Katholieke Universiteit Leuven (KUL) Leuven Belgium; ^9^ Department of Medical Oncology University Hospital Antwerp Edegem Belgium; ^10^ Center for Oncological Research (CORE) Integrated Personalized and Precision Oncology Network (IPPON) University of Antwerp Antwerp Belgium

**Keywords:** cachexia, iron, metabolism, mitochondria, muscle, Cancer, Metabolism, Musculoskeletal System

## Abstract

Cachexia is a wasting syndrome characterized by devastating skeletal muscle atrophy that dramatically increases mortality in various diseases, most notably in cancer patients with a penetrance of up to 80%. Knowledge regarding the mechanism of cancer‐induced cachexia remains very scarce, making cachexia an unmet medical need. In this study, we discovered strong alterations of iron metabolism in the skeletal muscle of both cancer patients and tumor‐bearing mice, characterized by decreased iron availability in mitochondria. We found that modulation of iron levels directly influences myotube size *in vitro* and muscle mass in otherwise healthy mice. Furthermore, iron supplementation was sufficient to preserve both muscle function and mass, prolong survival in tumor‐bearing mice, and even rescues strength in human subjects within an unexpectedly short time frame. Importantly, iron supplementation refuels mitochondrial oxidative metabolism and energy production. Overall, our findings provide new mechanistic insights in cancer‐induced skeletal muscle wasting, and support targeting iron metabolism as a potential therapeutic option for muscle wasting diseases.

## Graphical abstract

Cancer induces striking alterations in iron metabolism of the skeletal muscle and iron supplementation prevents muscle atrophy and loss of strength.

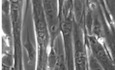

## Introduction

In healthy humans, skeletal muscle makes up to 40% of the total body mass (Janssen *et al,* 2000), of which 20% are constituted by proteins. Massive skeletal muscle atrophy is the hallmark of a multi‐organ wasting disorder known as cachexia, which causes severe asthenia and intolerance to therapies in patients with chronic diseases such as cardiac failure, COPD, and notably cancer, leading to poor clinical outcomes (Fearon *et al,* 2011; Porporato, 2016). Indeed, the prevalence of cachexia reaches 80% in advanced‐stage cancer patients and has been estimated to directly cause at least 20% of all cancer‐related deaths (Tisdale, 2002).

In cancer cachexia, systemic alterations contribute to the uncontrollable decrease in quality of life, insulin resistance, liver dysfunction, chronic inflammation, and even altered gut microbiota and nutrient absorption (Porporato, 2016). Remarkably, iron deficiency is diagnosed in more than half of patients afflicted with colorectal, lung, and pancreatic cancers, which are also associated with high prevalence of cachexia (Ludwig *et al,* 2013). Chronic inflammation hampers iron absorption from the diet and causes iron retention in reticuloendothelial cells, which results in insufficient iron availability to meet the body’s needs. Iron is indeed a versatile cofactor essential to a multitude of vital metabolic processes including oxygen supply, DNA synthesis, redox homeostasis, or energy metabolism. Energy production directly depends on the availability of iron. It is indispensable for the activity of several mitochondrial enzymes involved in the TCA cycle and the electron transport chain, where iron is found under the form of heme or iron–sulfur cluster (ISC). Moreover, iron has been shown to directly regulate mitochondrial biogenesis, highlighting the sensitivity of these organelles to iron availability (Rensvold *et al,* 2016). Notably, both iron accumulation and iron deficiency are detrimental to mitochondrial function. Cellular iron homeostasis is thus a tightly regulated process involving a broad variety of proteins responsible for its transport (transferrin), uptake (transferrin receptor/TFR1), storage (ferritin/FT), and export (ferroportin/FPN1). The fine tuning of intracellular iron metabolism is made possible by the Iron Responsive Element/Iron Responsive Protein (IRE/IRP) system exerting a major control on the translation of several key iron‐related proteins.

In the skeletal muscle, iron is particularly needed to support the high metabolic activity required for ATP generation, a requisite for contraction and movement. While mitochondrial dysfunction (in particular, decreased oxidative capacity, inefficient energy production, and altered mitochondrial dynamics) has been proven to promote skeletal muscle wasting in cachexia (Boengler *et al*, 2017; Abrigo *et al,* 2019), little is known about the consequence of altered iron levels on skeletal muscle function and mass. Importantly, iron deficiency is present in the vast majority of cancer patients and has been associated to advanced stage and poor prognosis (Ludwig *et al,* 2013). Hence, we decided to investigate the role of iron metabolism in cancer cachexia‐related muscle wasting.

## Results

### Iron deficiency induces skeletal muscle atrophy

**Figure 1:**
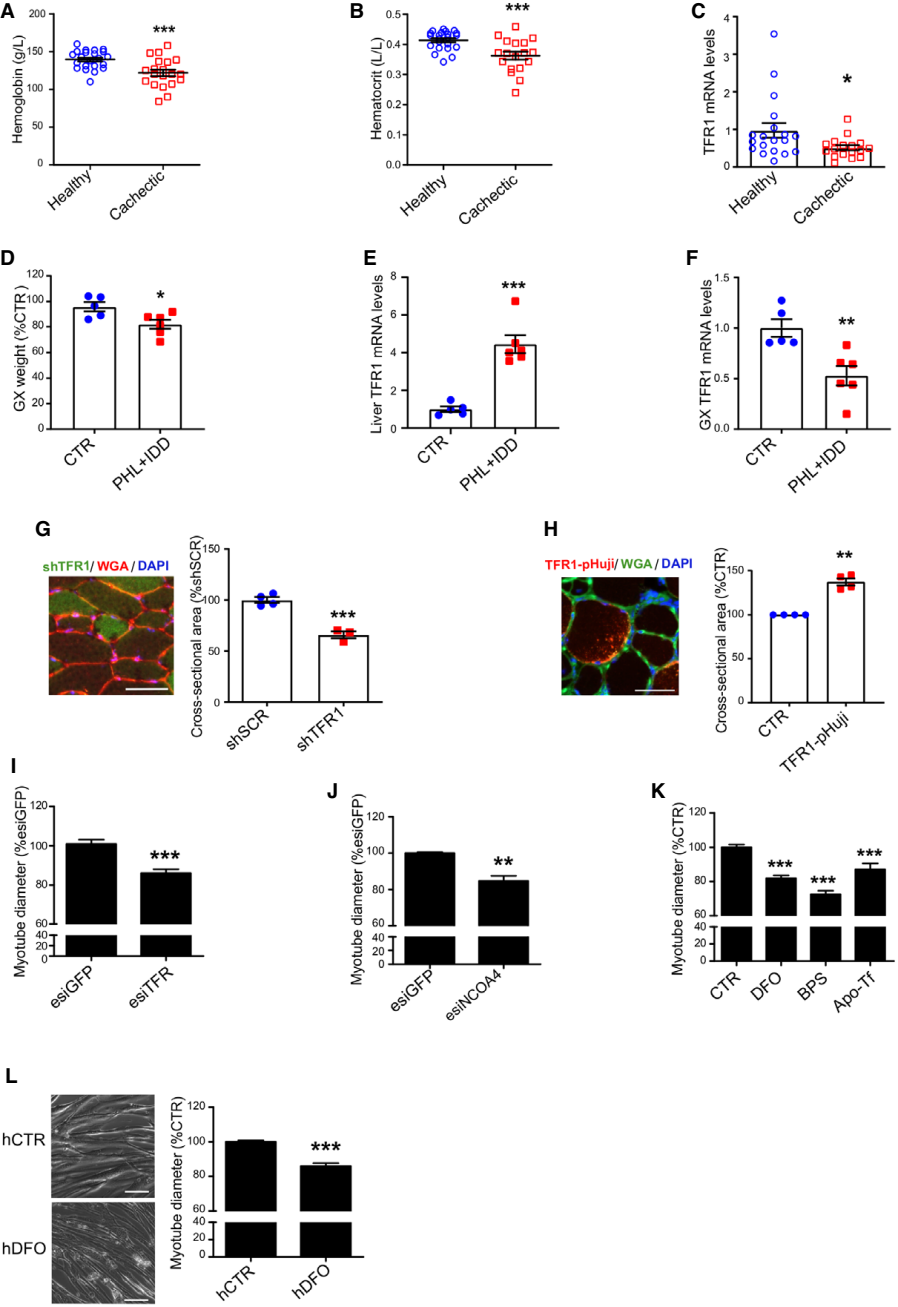
Iron deficiency induces skeletal muscle atrophy A, BHemoglobin (A) and hematocrit (B) levels of healthy subjects and cachectic cancer patients presenting a body weight loss superior to 10% of initial body weight (19 healthy subjects, 17 cachectic patients).CTFR1 mRNA levels in muscle biopsies from cancer patients of late stage cachexia with at least 10% total body weight loss (19 healthy subjects, 17 cachectic patients).DGastrocnemius weight in mice subjected to iron deprivation by feeding with an iron‐deficient diet (IDD) combined to a phlebotomy (PHL) (*n* = 5–6).ETFR1 mRNA levels in the liver of mice subjected to iron deprivation by feeding with an iron‐deficient diet (IDD) combined to a phlebotomy (PHL) (*n* = 5–6).FTFR1 mRNA levels in the gastrocnemius of mice subjected to iron deprivation by feeding with an iron‐ deficient diet (IDD) combined to a phlebotomy (PHL) (*n* = 5–6).GCross‐sectional area of skeletal muscle fibers transfected with shSCR (scramble) and shTFR1 (*n* = 3–4) and representative picture of shTFR1 transfected fibers. Scale bar = 50 µm.HCross‐sectional area of skeletal muscle fibers transfected with TFR‐pHuji (*n* = 4) and representative picture of TFR‐pHuji transfected fibers. Scale bar = 50 µm.I, JDiameter of TFR1 (I) or NCOA4 (J) knocked down C2C12 myotubes at day 3 post‐transfection (*n* = 7 and *n* = 3 respectively).KDiameter of C2C12 myotubes after 48 h treatment with Deferoxamine (DFO), bathophenanthroline disulfonate (BPS), or apo‐transferrin (Apo‐Tf).LRepresentative pictures and diameter measurements of human myoblast‐derived myotubes after 48 h treatment with DFO (*n* = 3). Scale bar = 50 µm. Data information: For all data, *n* represents the number of biological replicates. Statistical significance was calculated by unpaired, two‐tailed Student’s *t*‐test. Data are mean ± SEM. *P < 0.05, **P < 0.01, ***P < 0.001. Source data are available online for this figure.

**Figure EV1:**
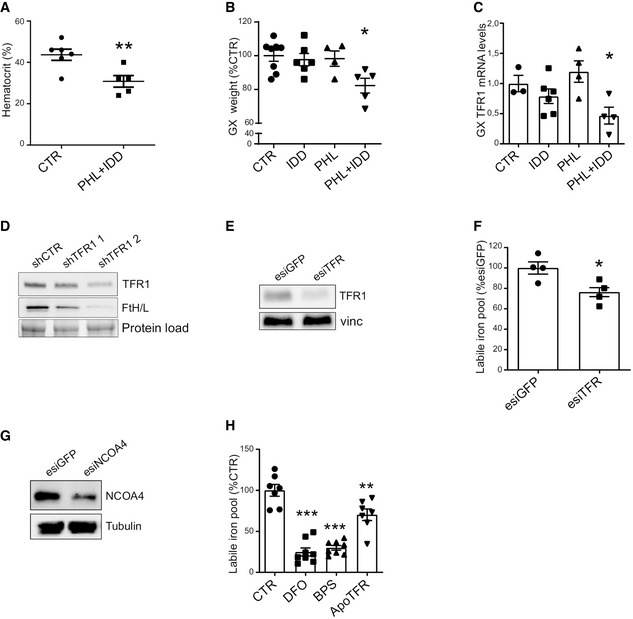
Iron deficiency induces skeletal muscle atrophy Hematocrit in mice subjected to iron deprivation by a combination of iron‐deficient diet (IDD) and phlebotomy (PHL) (*n* = 5–6).Gastrocnemius weight in mice subjected to iron deprivation by iron‐deficient diet (IDD), phlebotomy (PHL), or a combination of both (*n* = 4–8).Gastrocnemius TFR1 mRNA levels in mice after iron deprivation by iron‐deficient diet (IDD), phlebotomy (PHL) or a combination of both (*n* = 3–6).Representative Western blot of TFR1 and ferritin after transfection with shTFR1‐pGFP in 3T3 cells (*n* = 3).Representative Western blot of TFR1 after knockdown in C2C12 myotubes (*n* = 3).Labile iron pool in C2C12 myotubes after TFR1 knockdown (*n* = 3).Representative Western blot of NCOA4 after knockdown in C2C12 myotubes (*n* = 3).Labile iron pool in C2C12 myotubes treated with iron chelators DFO, BPS, or apo‐transferrin (*n* = 3). Data information: For all data, *n* represents the number of biological replicates. Statistical significance was calculated by unpaired, two‐tailed Student’s *t*‐test (A, F) or one‐way Anova with Bonferroni’s correction (B, C, and H). Data are mean ± SEM. *P < 0.05, **P < 0.01, ***P < 0.001.

Iron deficiency is highly prevalent in cancer patients and has been associated to advanced stage and poor prognosis (Ludwig *et al,* 2013). To assess the effects of cancer‐induced iron deficiency on skeletal muscle metabolism, we analyzed the transcript levels of the main cellular iron importer, transferrin receptor 1 (TFR1) in a cohort of cancer patients presenting body weight loss and anemia (Fig 1A and B). During iron deficiency, cells normally increase iron import through TFR1 to maintain homeostasis (Camaschella *et al,* 2020). Surprisingly, the patients displayed decreased TFR1 (Fig 1C). To simulate the condition of iron‐deficient anemia typical of cancer patients, we induced severe anemia in mice by combined phlebotomy and iron‐free diet (Fig EV1A). This treatment resulted in muscle atrophy (Figs 1D and EV1B), supporting the hypothesis of an involvement of iron homeostasis in the onset of cancer‐associated muscle wasting. As expected, iron deficiency promoted TFR1 upregulation in liver (Fig 1E), presumably to ensure the necessary supply of iron to the organ (Camaschella *et al,* 2020). However, TFR1 was downregulated in skeletal muscle of iron‐deficient mice (Figs 1F and EV1C), suggesting a different response of this tissue to iron deprivation. To study the role of TFR1 expression on muscle mass, we transfected TFR1‐silencing or TFR1‐overexpressing plasmid by electroporating the tibialis anterior of healthy mice. TFR1 silencing was sufficient to induce fiber atrophy (Figs 1G and EV1D), while TFR1 overexpression triggered hypertrophy in the positive fibers (Fig 1H). Coherently, inhibition of iron import by silencing TFR1 induced significant myotube atrophy *in vitro* and decrease of the labile iron pool (Figs 1I and EV1E and F). Similarly, blocking intracellular iron mobilization by silencing NCOA4 (a cytoplasmic protein that mediates autophagic degradation of ferritin (Bellelli *et al,* 2016)), also caused significant myotube atrophy (Figs 1J and EV1G). Furthermore, to assess the direct impact of iron deficiency on muscle, we evaluated the effect of iron chelation on murine and human myotubes. Treatment with different iron chelators, namely deferoxamine (DFO), bathophenanthroline disulfonic acid (BPS), and apotransferrin (Apo‐Tf), which is known to decrease transferrin saturation, led to a reduction in C2C12 myotube diameter and labile iron pool (Figs 1K and EV1H). Consistently, iron chelation by DFO exerted the same atrophic effect on human myotubes (Figs 1L). In summary, we found that cachectic cancer patients have decreased muscular TFR1 expression, and decreased iron availability is sufficient to induce skeletal muscle atrophy *in vivo* and myotube diameter reduction *in vitro*.

### Altered iron metabolism in the skeletal muscle is a feature of cancer‐induced cachexia

**Figure 2:**
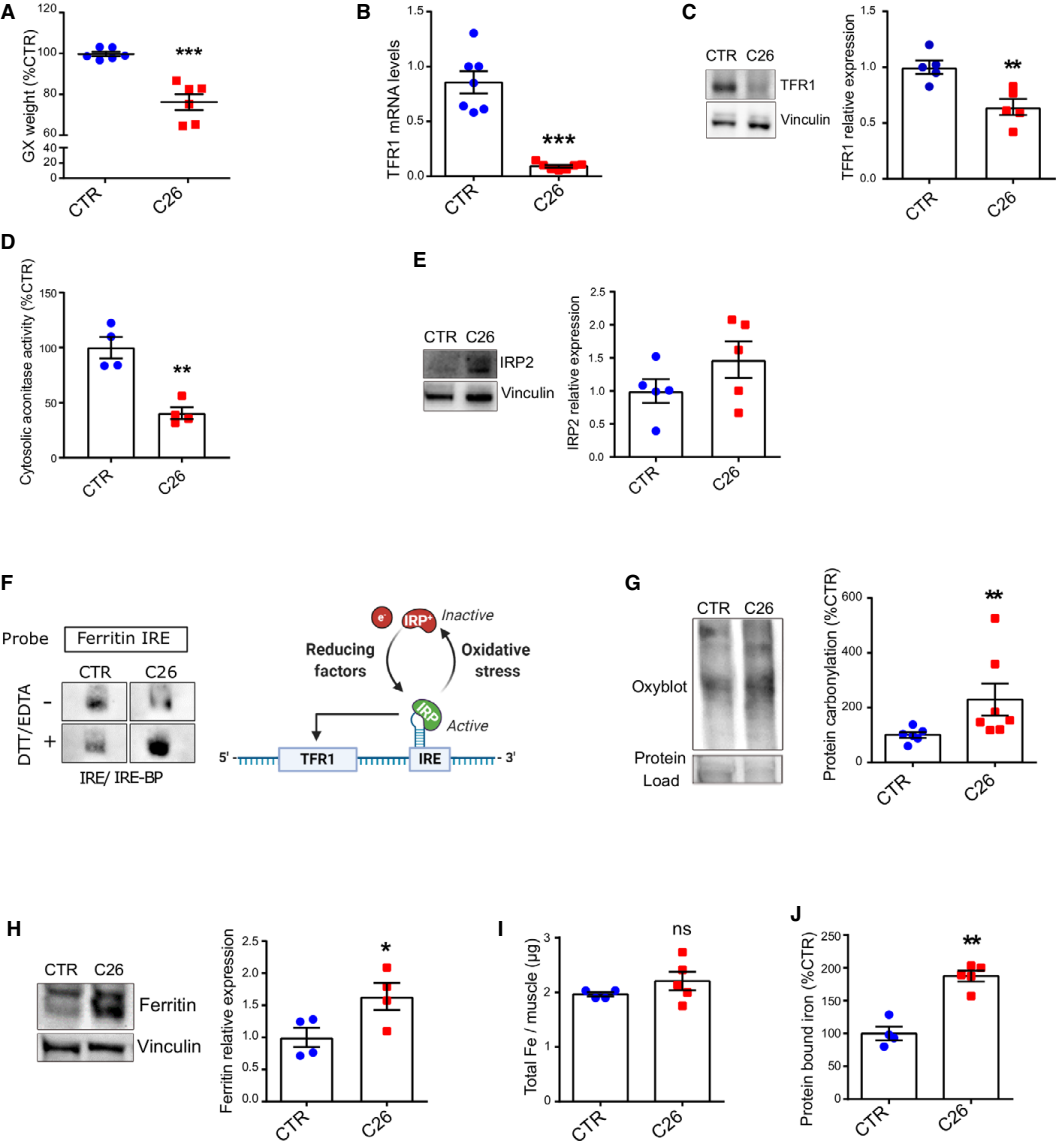
Altered iron metabolism in the skeletal muscle is a feature of cancer‐induced cachexia AGastrocnemius weight normalized to tibial length in C26 tumor‐bearing mice on day 12 post C26‐injection (*n* = 6–7).BTFR1 mRNA levels normalized to 18s (*n* = 6–7).CTFR1 protein expression and densitometric quantification in mouse gastrocnemius (*n* = 5).DCytosolic aconitase activity in mouse quadriceps (*n* = 4) measured following subcellular fractionation (*n* = 4).ERepresentative Western blot of IRP2 in mouse gastrocnemius and densitometric quantification (*n* = 5).FBinding of IRE‐BPs to the ferritin IRE. The biotin‐labeled IRE probe was incubated with cytosolic gastrocnemius extracts from mice, in native or reducing conditions (with EDTA and DTT) (*n* = 3).GRepresentative protein carbonylation blot and densitometric quantification in mouse quadriceps (*n* = 6–7).HRepresentative Western blot of ferritin in mouse gastrocnemius and densitometric quantification (*n* = 4).I, JICP‐MS quantification of total (I) and protein‐bound (J) iron in mouse quadriceps (*n* = 4–5). Data information: For all data, *n* represents the number of biological replicates. Statistical significance was calculated by unpaired, two‐tailed Student’s *t*‐test. Data are mean ± SEM. *P < 0.05, **P < 0.01, ***P < 0.001. Source data are available online for this figure.

**Figure EV2:**
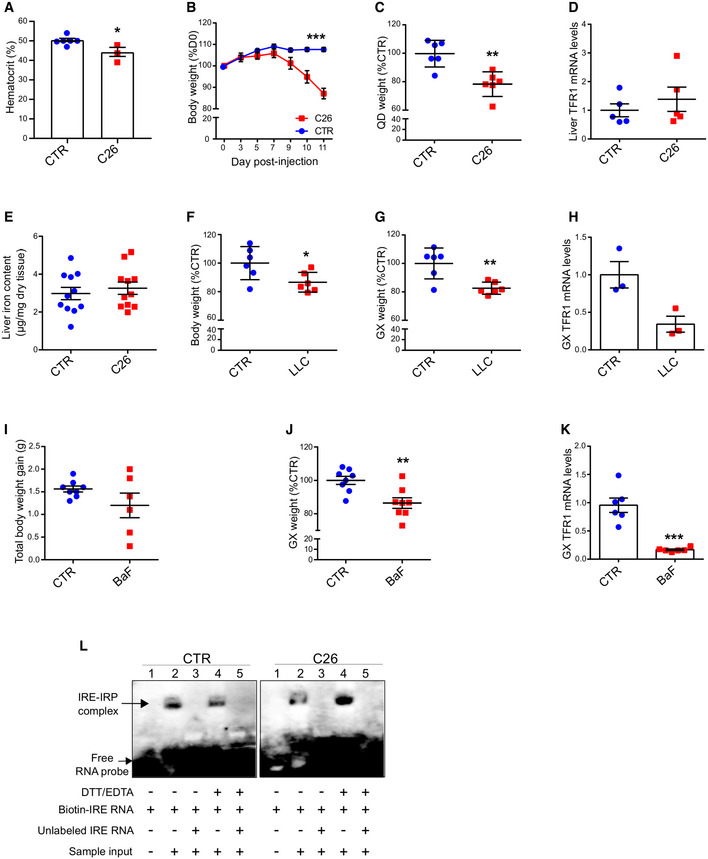
Altered iron metabolism in the skeletal muscle is a feature of cancer‐induced cachexia AHematocrit levels of C26 tumor‐bearing mice at day 12 post C26 injection (*n* = 3–6).BBody weight evolution of mice after C26 injection (*n* = 6).CQuadriceps weight of C26 tumor‐bearing mice normalized to tibial lenght (*n* = 6).DLiver TFR1 mRNA levels normalized to 18s in C26 tumor‐bearing mice (*n* = 5).ETotal liver iron content in C26 tumor‐bearing mice (*n* = 11).F, GTotal body weight (F) and gastrocnemius weight (G) in LLC tumor‐bearing mice (*n* = 5–6).HTFR1 mRNA levels normalized to 18s in the gastrocnemius of LLC tumor‐bearing mice (*n* = 3).I, JTotal body weight gain (I) and gastrocnemius weight (J) of BaF‐transplanted mice (*n* = 6–8).KTFR1 mRNA levels normalized to 18s in the gastrocnemius of BaF‐transplanted mice (*n* = 6–8).LRaw blots of Fig 2F RNA electrophoretic mobility shift assay (REMSA). The biotin‐labeled IRE probe was incubated without (lane 1) or with cytosolic gastrocnemius extracts from CTR and C26 tumor‐bearing mice, in native (lane 2) or reducing conditions (lane 4). Where indicated, unlabeled IRE probe was added in 200‐fold molar excess (lanes 3 and 5) (*n* = 3). Data information: For all data, *n* represents the number of biological replicates. Statistical significance was calculated by unpaired, two‐tailed Student’s *t*‐test (A, C‐K) or ordinary two‐way Anova (B). Data are mean ± SEM. *P < 0.05, **P < 0.01, ***P < 0.001.

To confirm the link between cancer cachexia and iron metabolism in the skeletal muscle, we recreated cancer cachexia in mice using the C26‐colon cancer model in Balb/C mice, which led to significant hematocrit reduction, total body weight loss, and muscle mass reduction (Figs 2A and EV2A–C). In line with the human data, cachectic mice showed a drastic reduction of TFR1 in the skeletal muscle (Fig 2B and C) despite no change in liver TFR1 or hepatic iron content (Fig EV2D and E), suggesting that the regulation of iron metabolism is organ‐specific. Muscle TFR1 downregulation was further confirmed in two different murine cachexia models, namely, LLC (Lewis Lung Carcinoma) and BaF3 (murine interleukin 3‐dependent pro‐B cell line) (Fig EV2F–K). Moreover, we assessed iron‐sensing RNA‐binding proteins mediating post‐transcriptional regulation of iron metabolism in mammalian cells (Meyron‐Holtz *et al*, 2004; Wang *et al*, 2020). We observed a decrease in cytosolic aconitase activity (hence a switch to iron‐regulatory protein/IRP1) (Fig 2D), and an upregulation of iron‐regulatory protein 2 (IRP2) (Fig 2E), indicating an iron‐deficient state in the skeletal muscle of tumor‐bearing mice. As IRP activity should drive TFR1 expression via iron‐responsive element (IRE), we measured the activity of the IRE‐IRP system in cachectic muscles by RNA electrophoretic mobility shift assay (REMSA). Despite the decreased aconitase function of IRP1 and the overexpression of IRP2, we observed a lower RNA‐binding activity to the IRE site of ferritin (FT) in cachectic samples compared to control in native conditions (Figs 2F and EV2L). The phenotype appeared to be linked to protein oxidation. Indeed, by performing the assay in reducing condition, we evidenced the opposite pattern, with cachectic samples presenting a higher IRE‐binding, suggesting an oxidative damage, which is known to negatively regulate IRP2 activity (Gehring *et al,* 1999). The oxidative stress in skeletal muscle of C26 tumor‐bearing mice was further confirmed by upregulated protein carbonylation (Fig 2G). In addition, we observed an overexpression of FT (Fig 2H), which is in line with impaired IRP activity (Cairo *et al,* 1996). Coherently, cachectic muscles showed significantly increased protein‐chelated iron despite no change in total iron content (Fig 2I and J).

### Cachectic muscles are characterized by mitochondrial iron deficiency and impaired oxidative metabolism

**Figure 3:**
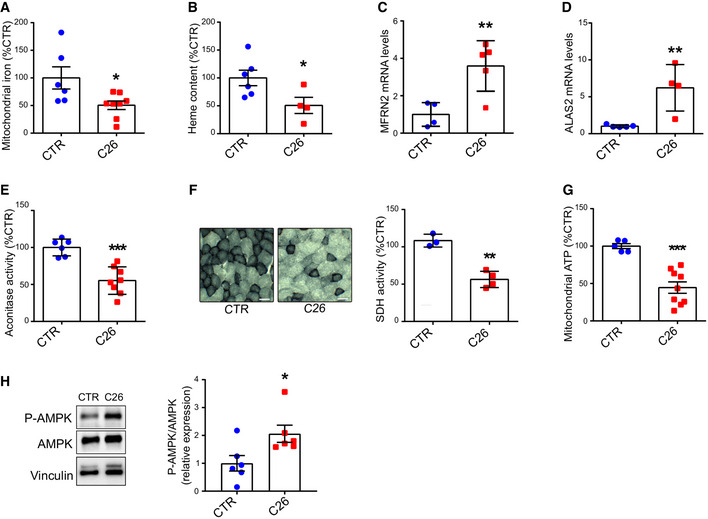
Cachectic muscles are characterized by mitochondrial iron deficiency and impaired oxidative metabolism AICP‐MS quantification of mitochondrial iron in mouse quadriceps (*n* = 6–8).BGastrocnemius heme content quantified by fluorescent heme assay (*n* = 4–6).C, DmRNA levels of mitochondrial iron importer MFRN2 (C) and the rate limiting enzyme of heme synthesis ALAS2 (D) normalized to 18s in mouse gastrocnemius (*n* = 4–5).EAconitase activity in mouse quadriceps lysates normalized to protein content (*n* = 6–8).FSuccinate Dehydrogenase activity staining in transversal sections of mouse gastrocnemius and corresponding intensity quantification (*n* = 3–4). Scale bar = 50 µm.GMitochondrial ATP content in mouse quadriceps (*n* = 5–9).HRepresentative Western blot and densitometric quantification of phospho‐AMPK and total AMPK in mouse gastrocnemius (*n* = 6). Data information: For all data, *n* represents the number of biological replicates. Statistical significance was calculated by unpaired, two‐tailed Student’s *t*‐test. Data are mean ± SEM. *P < 0.05, **P < 0.01, ***P < 0.001. Source data are available online for this figure.

In most cells, a major amount of iron is taken up by mitochondria for the production of ISCs and heme. In the skeletal muscle of C26 tumor‐bearing mice, we found a significant reduction of mitochondrial iron and total heme content (Fig 3A and B), as well as upregulated levels of mitochondrial iron importer mitoferrin 2 (MFRN2) and of the rate‐limiting enzyme of heme synthesis aminolevulinic acid synthase 2 (ALAS2) (Fig 3C and D) (Barman‐Aksozen *et al,* 2019). Given that iron is essential for several enzymes involved in the TCA cycle and mitochondrial oxidative metabolism (OXPHOS) (Xu *et al,* 2013), we assessed the enzymatic activity of two iron–sulfur proteins, aconitase (ACO) and succinate dehydrogenase (SDH), and found a 50% reduction in the activity of both enzymes in cachectic muscles (Fig 3E and F). Along with these alterations, we observed a drop in mitochondrial ATP (Fig 3G) and increased AMPK phosphorylation, denoting mitochondrial dysfunction in cachectic muscles (Zhao *et al,* 2016) (Fig 3H). In summary, tumor‐bearing mice display remarkable alterations in muscle iron metabolism coupled with mitochondrial dysfunction, which has been linked to muscle atrophy.

### Iron supplementation prevents mitochondrial dysfunction and atrophy *in vitro*

**Figure 4:**
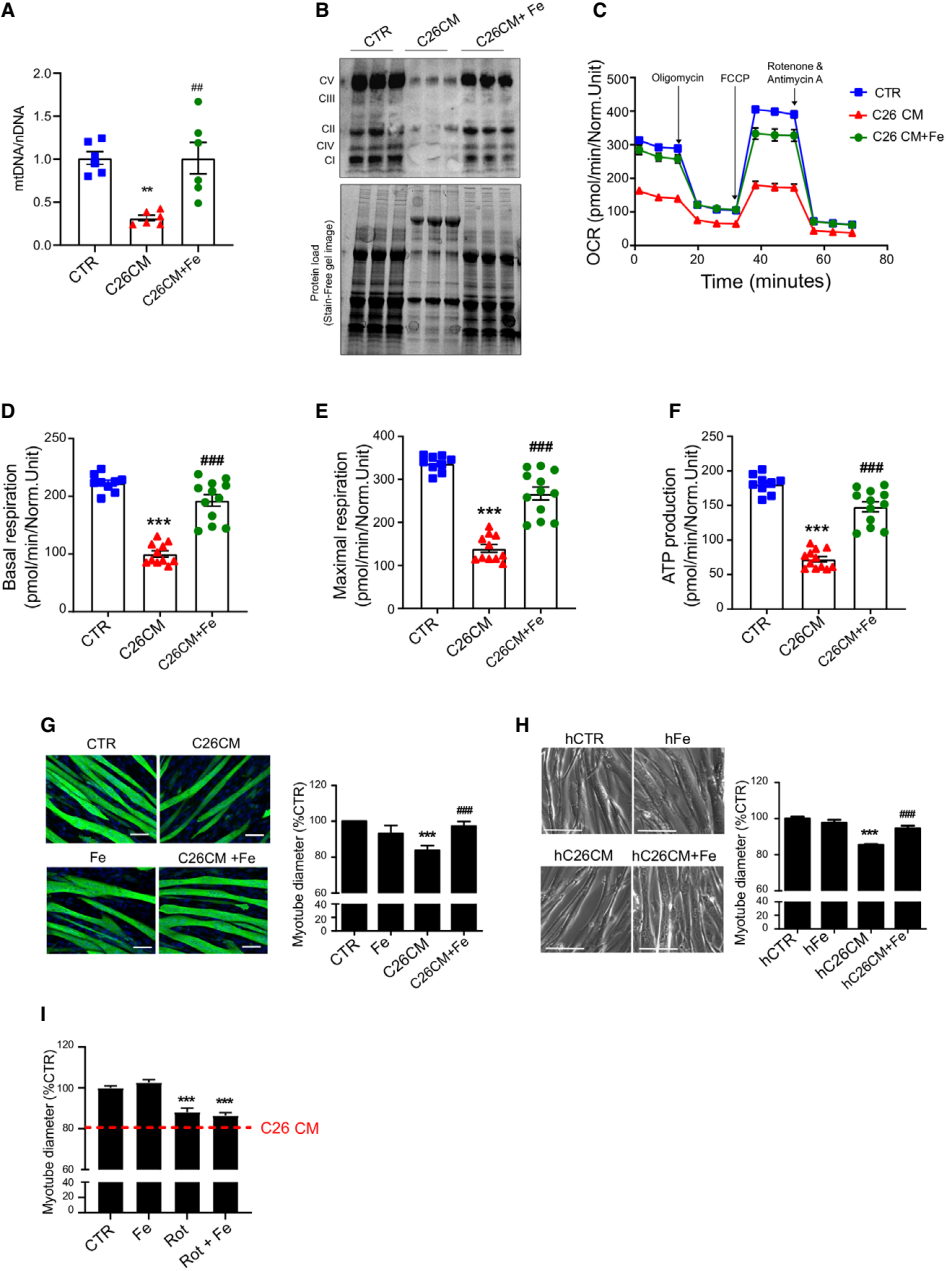
Iron enhances mitochondrial function and prevents cancer‐induced myotube atrophy AqPCR analysis of mitochondrial DNA (mtDNA) on nuclear DNA (nDNA) in C2C12 myotubes treated for 48 h with C26 CM and ferric citrate (*n* = 6).BWestern blot of mitochondrial OXPHOS respiratory complexes in C2C12 myotubes treated for 48 h with C26 CM and ferric citrate (*n* = 3).C–FProfile of oxygen consumption rate OCR (C), basal OCR (D), maximal OCR (E), and OCR used for mitochondrial ATP production (F) in C2C12 myotubes after 48 h treatment with C26 CM and ferric citrate supplementation. Data normalized to protein content (*n* = 9–12).G, HRepresentative microscopic pictures and diameter of C2C12 myotubes stained for myosin heavy chain (G) or human myoblast‐derived myotubes (H) after 48 h treatment with C26 CM and ferric citrate (*n* = 3 per condition). Scale bars = 50 and 100 µm, respectively.IDiameter of C2C12 myotubes treated with rotenone and ferric citrate (*n* = 3). Data information: For all data, *n* represents the number of biological replicates. Statistical significance was calculated by unpaired, two‐tailed Student’s *t*‐test (D‐E), or one‐way Anova with Bonferroni’s correction (F‐G). Data are mean ± SEM. **P < 0.01, ***P < 0.001 compared to control and ##*P* < 0.01, ###*P* < 0.001 compared to C26 CM‐treated group. Source data are available online for this figure.

**Figure EV3:**
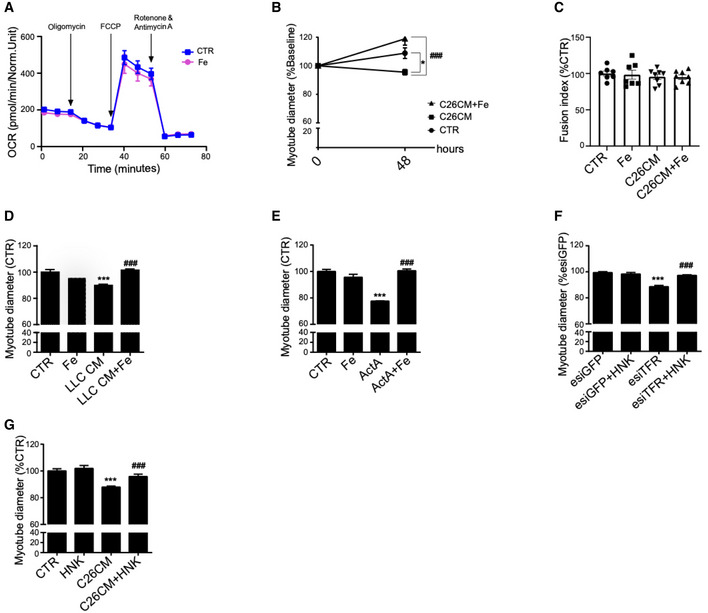
Iron enhances mitochondrial function and prevents cancer‐induced myotube atrophy Profile of oxygen consumption rate OCR in C2C12 myotubes after 48 h treatment with ferric citrate (*n* = 6).Myotube diameter normalized to Day 0 values (*n* = 3).Fusion index of C2C12 myotubes treated with C26 CM and ferric citrate for 48 h (*n* = 7–8).Diameter of C2C12 myotubes treated with LLC CM and iron citrate for 48 h (*n* = 3).Diameter of C2C12 myotubes treated with Activin A (ActA) and ferric citrate for 48 h (*n* = 3).Diameter of TFR1‐silenced C2C12 myotubes after 24 h treatment with iron ionophore hinokitiol (HNK) (*n* = 3).Diameter of C2C12 myotubes treated with C26 CM and HNK for 48 h (*n* = 3). Data information: For all data, *n* represents the number of biological replicates. Statistical significance was calculated by one‐way Anova with Bonferroni’s correction (A‐E). Data are mean ± SEM. ***P < 0.001 compared to control and ###*P* < 0.001 compared to conditioned medium, ActA, or esiTFR‐treated group.

In line with the *in vivo* data, we found considerably decreased mitochondrial DNA and proteins in myotubes treated with C26 conditioned medium (CM) (Fig 4A and B). To verify the hypothesis that cancer‐associated iron shortage could cause mitochondrial dysfunction, a known feature of muscle atrophy (Liu *et al,* 2016), we supplemented atrophic C2C12‐myotubes with iron. Iron supplementation fully rescued the C26 CM‐induced reduction of the oxygen consumption rate (OCR) (Fig 4C–F), while showing no effect on control myotubes (Fig EV3A). Moreover, microscopic analysis confirmed that iron supplementation prevents C26‐induced diameter decreased in both murine and human myotubes *in vitro* (Fig 4G and H). Noteworthy, although iron substantially increases the diameter of C26‐treated myotubes over time, we observed no change in the fusion index, excluding a potential effect on myogenesis (Fig EV3B and C). Consistently with the C26 model, iron supplementation prevented other cancer CM‐ and activin A (ActA)‐induced atrophy (Zhou *et al,* 2020) in murine myotubes (Fig EV3D and E). Similarly, normalization of iron levels using the iron–ionophore hinokitiol (Grillo *et al*, 2017) also rescued myotube atrophy induced by TFR1‐silencing or C26 CM (Fig EV3F and G). Importantly, low‐dose rotenone (complex 1 inhibitor) caused a mild atrophy that cannot be rescued by iron, indicating that the protective effects of iron are mediated by mitochondrial function (Fig 4I). Altogether, these data demonstrate that C26 CM treatment recapitulates the mitochondrial dysfunction observed *in vivo* (Fig 3), which can be fully rescued *in vitro* by iron supplementation.

### Iron supplementation rescues skeletal muscle mass and mitochondrial function

**Figure 5:**
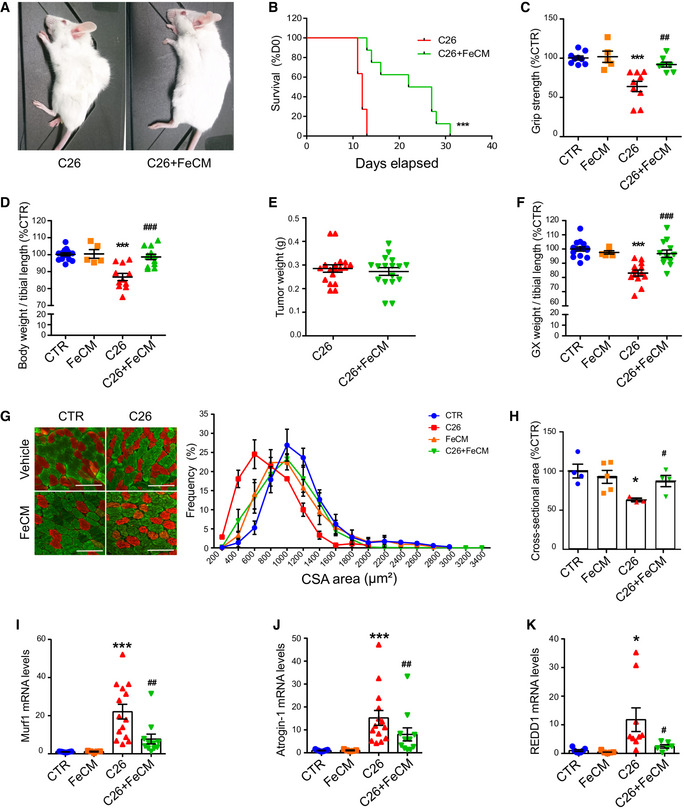
Iron supplementation prevents cancer‐induced cachexia ARepresentative images of C26 tumor‐bearing mice receiving saline solution (left) or FeCM 15 mg/kg I.V. injection (right) taken at day 12 after C26 injection.BKaplan–Meier depicting the survival of C26 tumor‐bearing mice after I.V. injection of saline or iron every 5 days post C26‐injection (3‐month‐old Balb/C, *n* = 8–11).CGrip strength of mice measured at day 12 post C26 injection, normalized to average strength of the control group (*n* = 5–9).DFinal body weight of C26 tumor‐bearing mice after iron supplementation at day 12 post C26 injection (*n* = 5–12).EFinal weight of total tumor mass extracted from mice after sacrifice (*n* = 17).FGastrocnemius weight normalized to tibial length of C26 tumor‐bearing mice after iron supplementation at day 12 post C26 injection (*n* = 5–12).G, HImmunofluorescent staining of myosin heavy chain fast (green) and slow (red) of transversal sections of gastrocnemius (midbelly) with corresponding frequency distribution (G) and average cross‐sectional areas (H) (*n* = 3–5). Scale bar = 100 µm.I–KmRNA levels of Murf 1 (I), Atrogin 1 (J), and REDD1 (K) normalized to GAPDH in gastrocnemius (*n* = 5–14). Data information: For all data, *n* represents the number of biological replicates. Statistical significance was calculated by one‐way Anova with Bonferroni’s correction, or chi‐square test for the survival curves (B). Data are mean ± SEM. *P < 0.05 and ***P < 0.001 compared to control and #*P* < 0.05, ##*P* < 0.01, ###*P* < 0.001 compared to C26‐untreated group. Source data are available online for this figure.

**Figure EV4:**
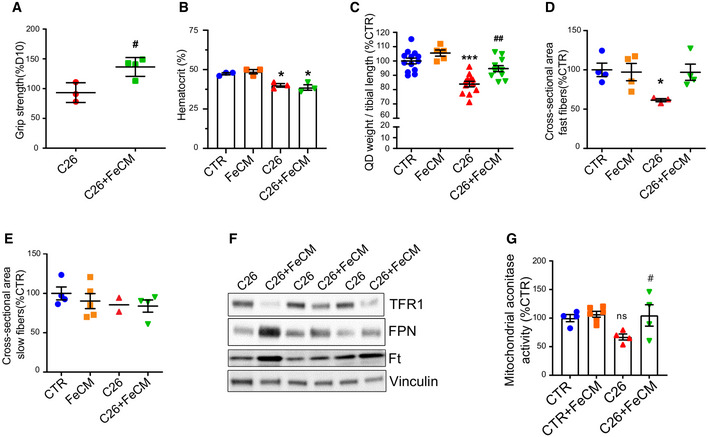
Iron supplementation prevents cancer‐induced cachexia AGrip strength of C26 tumor‐bearing mice measured at day 11 and normalized to day 10 (*n* = 3–4).BHematocrit in tumor‐bearing mice measured on day 12 post C26 injection (*n* = 3)CQuadriceps weight normalized to tibial length of C26 tumor‐bearing mice at day 12 post C26 injection (*n* = 5–12).D, EAverage cross‐sectional area of fast (D) and slow (E)–twitch muscle fibers (*n* = 2–5).FRepresentative Western blot of iron metabolism proteins in tumor extracts from C26 tumor‐bearing mice showing a physiological response to iron loading (*n* = 3).GMitochondrial aconitase activity in quadriceps of C26 tumor‐bearing mice supplemented with FeCM (*n* = 4–6). Data information: For all data, *n* represents the number of biological replicates. Statistical significance was calculated by unpaired, two‐tailed Student’s *t*‐test (A) or one‐way Anova with Bonferroni’s correction (B‐D). Data are mean ± SEM. *P < 0.05, ***P < 0.001 compared to control and #*P* < 0.05, ##*P* < 0.01compared to C26‐untreated group.

To assess if iron supplementation could prevent cancer‐induced muscle atrophy *in vivo*, C26 tumor‐bearing mice were *i.v*. treated with ferric carboxymaltose (FeCM) every 5 days post C26 injection. Remarkably, intravenous injections of iron resulted in healthier (smooth fur, no orbital discharge, no humpback) and more physically active mice that survived far beyond the usually fatal 2 weeks (Fig 5A and B). Of note, iron improved notably the grip strength within 24 h (Fig EV4A) and the protection was preserved until the end‐point of the experiment (Fig 5C), without affecting hematocrit (Fig EV4B). In addition, the loss of body weight and muscle mass occurring at day 12 after C26 injection was prevented in FeCM‐treated mice (Figs 5D–F and EV4C). Noteworthy, iron supplementation downregulated TFR1 in the tumor without affecting tumor growth (Figs 5E and EV4F). Consistently, immunostaining for myosin heavy chain revealed larger muscle fibers in the gastrocnemius of FeCM‐treated mice, especially in the fast‐twitch fibers, the most susceptible to atrophy (Fig 5G, in green). The protection from atrophy was further confirmed by the cross‐sectional area (CSA) distribution, showing a shift toward bigger areas in FeCM‐treated mice compared to C26 tumor‐bearing, untreated animals, and the average CSA (Figs 5G and H, and EV4D and E). These findings were further reinforced by a significant drop of FBXO32 (ATRO1), TRIM63 (MURF1), and DDIT4 (REDD1) mRNA levels, which are indicators of skeletal muscle atrophy (Fig 5I–K).

### Iron supplementation refuels mitochondrial oxidative metabolism and energy generation

**Figure 6:**
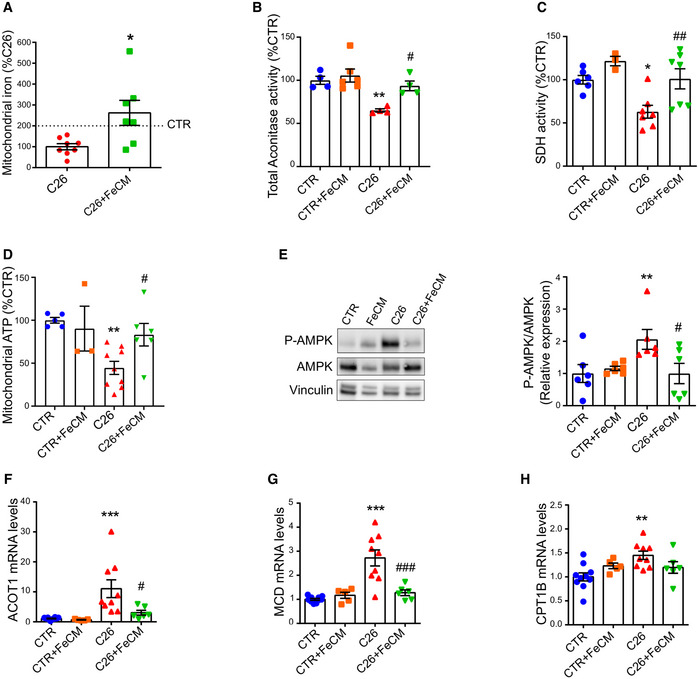
Iron supplementation rescues mitochondrial function AMitochondrial iron quantified by ICP‐MS in quadriceps of C26 tumor‐bearing mice after FeCM supplementation (*n* = 7–8).BAconitase activity of quadriceps lysates normalized to protein content (*n* = 3–7).CSuccinate Dehydrogenase activity staining of gastrocnemius transversal sections and resulting intensity quantification (*n* = 3–7).DMitochondrial ATP content determined by luminescence assay in quadriceps (*n* = 3–9).ERepresentative Western blot and densitometric quantification of phospho‐AMPK and total AMPK (stripped and re‐blotted) in the gastrocnemius of C26 tumor‐bearing mice after iron carboxymaltose supplementation (*n* = 6).F–HmRNA levels of ACOT 1 (F), MCD (G), and CPT1B (H) normalized to GAPDH in gastrocnemius (*n* = 5–10). Data information: For all data, *n* represents the number of biological replicates. Statistical significance was calculated by unpaired, two‐tailed Student’s *t*‐test. Data are mean ± SEM. *P < 0.05, **P < 0.01, ***P < 0.001 compared to control and #*P* < 0.05, ##*P* < 0.01, ###*P* < 0.001 compared to C26‐untreated group. Source data are available online for this figure.

Since our findings *in vitro* indicate that iron can refuel mitochondrial metabolism and maintain myotube mass, we sought to validate our hypothesis *in vivo*. After verifying the replenishment of mitochondrial iron in skeletal muscle (Fig 6A), we measured the activity of aconitase and succinate dehydrogenase and observed a significant recovery of enzymatic functionality following iron injection (Figs 6B and C, and EV4G). In agreement with these findings in mice showing restored mitochondrial metabolism upon iron treatment, we also found a significant increase in mitochondrial ATP content (Fig 6D) and coherently a drop in AMPK phosphorylation (Fig 6E). As AMPK drives fatty acid oxidation (FAO), which has been functionally linked to the cachectic process (Fukawa *et al,* 2016), we next evaluated the effect of iron injection on FAO. Consistently, iron supplementation reduced the C26‐induced upregulation of FAO genes (Fig 6F–H). Altogether, these data indicate that iron supplementation of tumor‐bearing mice mitigates energetic stress and catabolic pathways, which mediate the increase in muscle functionality and mass.

### Iron supplementation improves muscle strength in cancer patients

**Figure 7:**
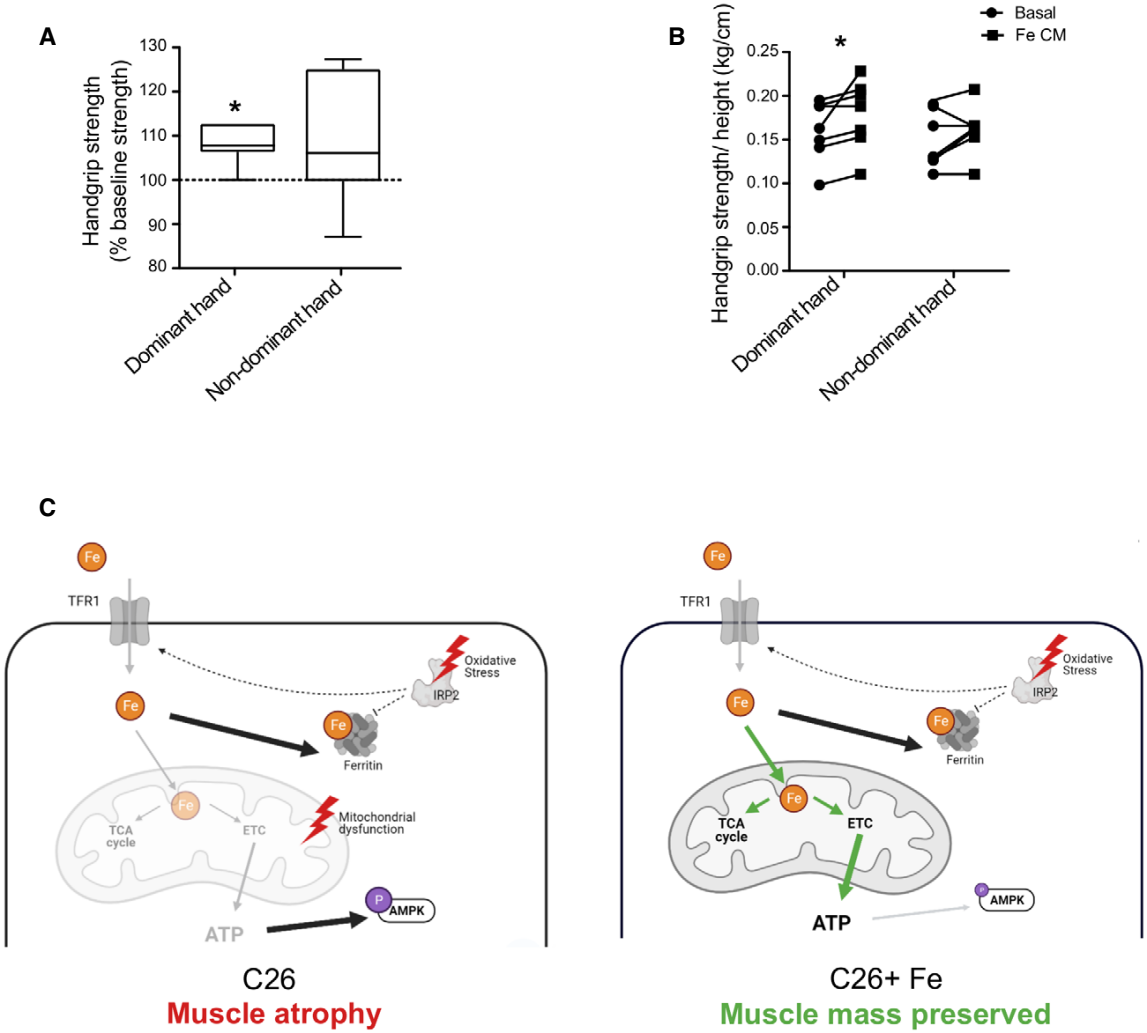
Iron supplementation improves muscle function in iron deficient cancer patients A, BGrip force of dominant or non‐dominant arm in iron‐deficient cancer patients, expressed in percentage of baseline force (A) and absolute values normalized to height (B) before/after single dose of iron carboxymaltose (15 mg/kg, 7 subjects).CWorking model: C26 tumor‐bearing mice present alterations of key iron metabolism proteins such as the downregulation of TFR1 and the upregulation FT in the skeletal muscle. Decreased cytosolic aconitase activity and the stabilization of IRP2 indicate a low iron status. However, IRP activity is hampered by oxidative stress and is no longer able to regulate TFR and FT (indicated by dashed lines). As a consequence, mitochondrial iron loading is low, and the decrease activity of iron‐dependent enzymes negatively affect the TCA cycle and the electron transport chain, resulting in decreased ATP production, AMPK activation, and muscle atrophy. Iron supplementation replenishes the mitochondrial iron pool and prevents mitochondrial dysfunction. Notably, it restores TCA cycle and electron transport chain activity resulting in higher ATP production, deactivation of AMPK, and preserved muscle mass. Data information: For all data, *n* represents the number of biological replicates. Statistical significance was calculated by paired, one‐tailed Student’s *t*‐test. Data are mean ± SEM. *P < 0.05. In graph (A), the boxes represent the range of values with the median value being the central band and whiskers the SEM.

Based on the results obtained in the pre‐clinical model of cancer‐associated muscle wasting, we measured the handgrip force in cancer patients with severe anemia, who reported muscle weakness, before and after FeCM injection (Table EV1). Improved strength was observed in the dominant hand of all patients, while more than half showed also increased force in the non‐dominant hand (Fig 7A and B) as short as 4§ days after the injection. Together with our data reporting TFR1 downregulation in the skeletal muscle of cachectic patients (Fig 1C), these findings indicate that altered iron metabolism may contribute to muscle weakness in cachectic patients. Consequently, these results highlight the contribution of iron on both muscle mass and functionality and suggest a new promising therapeutic strategy to counteract cancer‐induced skeletal muscle wasting. Altogether, our findings show that iron supplementation prevents cancer‐induced cachexia through a recovery of mitochondrial function (Fig 7C).

## Discussion

Our study indicates iron as a key element of skeletal muscle mass homeostasis through the maintenance of mitochondrial function. In particular, we evidenced that cancer causes striking iron dysregulation in cachectic muscle, and that muscle wasting is reversible by iron supplementation. Despite being the biggest tissue in the human body, the involvement of skeletal muscle in systemic iron homeostasis has been generally neglected. Nevertheless, skeletal muscle holds a substantial pool of iron that can be mobilized, as demonstrated by its ability to support erythropoiesis in healthy humans during high‐altitude hypoxia (Robach *et al,* 2007). Since iron deficiency and anemia are common in cancer patients (Ludwig *et al,* 2013) and are associated with several features of cachexia, such as impaired physical function, weakness, and fatigue, we hypothesized a link between altered iron metabolism and muscle wasting. We found that iron deficiency, either cancer‐induced or obtained with iron chelators, disrupts iron homeostasis in the skeletal muscle and leads to muscle atrophy in different murine models. Surprisingly, atrophic muscle paradoxically downregulate the iron importer TFR1 despite the lack of iron, highlighting the role of skeletal muscle as an expendable body compartment. Similarly, we found a consistent decrease of TFR1 in late‐stage anemic cancer cachexia patients while it has also been reported that chemotherapy negatively affects TFR1 levels in the skeletal muscle (Hulmi *et al,* 2018). TFR1 emerges as a direct player in muscle mass homeostasis, as we show that its expression is sufficient *per se* to regulate myofiber size *in vivo*. These data are also supported by previous work on muscle‐specific TFR1 KO mice which displayed severe systemic metabolic dysfunction (Barrientos *et al,* 2015). Intriguingly, skeletal muscle from C26 tumor‐bearing mice combine typical hallmarks of iron deficiency (*i.e*., IRP2 upregulation, decrease in both cytosolic and mitochondrial aconitase activity, and mitochondrial iron) with the ones of iron overload (*i.e*., TFR1 downregulation, increase in ferritin, and in protein‐bound iron). Such discrepancy can be explained by the impaired IRE‐IRP system, due to a significant oxidative damage, which can be reversed by reducing conditions *ex vivo* (Cairo *et al,* 1998; Mikhael *et al,* 2006). Consequently, cachectic muscles sequestrate intracellular iron, downregulate its import despite the need for iron. This phenotype matches the observations reported in several IRP2 knockout models (Meyron‐Holtz *et al,* 2004; Galy *et al,* 2010; Li *et al,* 2018). To understand whether the resulting inadequate levels of iron impact muscle fitness, we focused on the *in vitro* myotube model (both murine and human). Importantly, iron deprivation in myotubes, induced by TFR1 or NCOA4 knockdown, apotransferrin, DFO, or BPS treatment was sufficient to trigger atrophy, whereas iron replenishment proved to be protective against conditioned medium‐induced atrophy. Thus, we speculated that iron availability directly influences muscle mass. To this aim, we used ferric carboxymaltose (FeCM), a clinically approved supplement (Scott, 2018) known to enter in cells regardless of TFR1 expression (Marone *et al,* 1986). Surprisingly, intravenous administrations of FeCM rescued body mass, muscle mass and function, and even increased the viability of tumor‐bearing mice, in accordance with a previous study demonstrating that preventing muscle loss in cachexia resulted in longer survival (Zhou *et al*, 2010). From the mechanistic standpoint, our *in vitro* data shows that iron supplementation refuels mitochondrial function. The upregulation of MFRN2 and ALAS2 could be interpreted as a compensatory response of the skeletal muscle to mitochondrial iron deficiency. In particular, ALAS2 overexpression has been associated to muscle weakness and atrophy in transgenic mice (Peng *et al,* 2021). Indeed, mitochondrial dysfunction has been functionally linked to wasting, both in pathological conditions (Romanello *et al,* 2010) and aging (Tezze *et al,* 2017; Palla *et al,* 2021), while an increasing pool of data associates declined oxidative metabolism with muscle myopathies (Dziegala *et al,* 2018). Notably, cardiac iron concentration inversely correlated with disease severity in non‐ischemic heart failure patients (Hirsch *et al,* 2020). Of note, iron is essential for mitochondrial function (Levi & Rovida, 2009) as it catalyzes several bioenergetic processes and its deprivation causes impaired mitochondrial biogenesis, enhanced mitophagy as well as metabolic dysfunction (Allen *et al,* 2013; Rensvold *et al,* 2013; Bastian *et al,* 2019).

We specifically found in cachectic mice that reduced mitochondrial iron content impairs mitochondrial activity and promotes AMPK phosphorylation, reflecting increased catabolism. The oxidative stress generated by dysfunctional mitochondria might reinforce iron dysregulation via IRPs inhibition, creating a vicious cycle. Furthermore, mitochondrial dysfunction is known to trigger catabolic pathways, notably FAO (Romanello & Sandri, 2015) that has been widely associated with skeletal muscle wasting (Brown *et al,* 2017; Abrigo *et al,* 2019). Our results pointed at two major ISC proteins of the TCA cycle and electron transport chain that have strongly reduced enzymatic activities, aconitase, and SDH, corroborating with the decreased oxidative capacities and energetic inefficiency as features of cachexia (Argiles *et al,* 2014; Neyroud *et al,* 2019). This confirms and extends studies showing that iron deficiency decreases ISC proteins, cytochrome content, and total oxidative capacity in cancer cachexia (Maguire *et al,* 1982; Oexle *et al,* 1999; Leermakers *et al,* 2020). Our data indicate that adequate iron supply restores mitochondrial function, as reflected by ATP production increase, AMPK deactivation, and FAO reduction.

Noteworthy, iron‐induced muscle protection was independent from tumor growth, as iron supplementation did not alter tumor volume. This observation is in line with the fact that C26 tumors do not show iron addiction and respond to iron supplementation by downregulating the iron‐import machinery.

Of note, consistent with the *in vitro* data of rapid mitochondrial metabolism rescue, the beneficial effects of iron injection *in vivo* were unexpectedly fast as muscle strength was improved within 24 h. The rescue is therefore unlikely due to muscle stem cell differentiation/regeneration or erythropoiesis. In line with this hypothesis, anemia remained after iron supplementation in C26 tumor‐bearing mice.

Although we found that iron supplementation can restore muscle mass homeostasis, both *in vitro* and *in vivo*, we do not exclude other systemic effects of iron repletion, for example liver (Klempa *et al,* 1989) and adipose tissue function (Gao *et al,* 2015), or immune system modulation (Serra *et al,* 2020), which might contribute to the increased survival but require further studies. However, our *in vivo* electroporation data show that TFR1 levels can directly influence myofiber size in healthy mice, without confounding systemic factors such as inflammation. Coherently, our preliminary data from iron‐deficient patients demonstrated an improvement of handgrip strength within a few days after iron treatment, excluding the potential involvement of erythropoiesis, while previous data on cardiac failure patients showed beneficial effects after long‐term treatment (Jankowska *et al,* 2016). Nevertheless, this is a very preliminary study on a limited number of patients who required iron supplementation because of iron‐deficiency anemia; hence they lack a placebo group. Further studies will be essential to better define the impact of short‐term iron treatment on patients, including a control group, as well as the impact on blood parameters a few days following supplementation.

Our findings corroborate with the assumption that iron deficiency causes metabolic dysfunction and energy insufficiency in skeletal muscle (Dziegala *et al,* 2018). Indeed, both iron deficiency (Leermakers *et al,* 2020) (Higashida *et al,* 2020) and iron overload (Alves *et al,* 2021) are detrimental to muscle physiology. Previous work suggested that excessive iron could underlie muscle loss in sarcopenic gastric cancer patients (Zhou *et al,* 2020). However, our study indicates that the distribution of iron rather than its total amount influences muscle homeostasis. Notably, we show that the total amount of iron is not changed in cachectic muscle compared to healthy controls in our C26 model, but it is mostly sequestrated in the cytosol and lacking in the mitochondria. While we confirmed the phenotype of altered iron metabolism in several wasting models (related to cancer or not), the beneficial effects of iron supplementation remain to be validated in other cancer models given that some cancer types would rely more on iron than others (Hsu *et al,* 2020).

In conclusion, our findings establish a direct role of iron availability in the control of skeletal muscle mass. Therefore, iron supplementation restores skeletal muscle homeostasis via mitochondrial metabolism normalization, paving the way for a new therapeutic strategy to fight muscle atrophy in cachectic patients, but also in non‐cancer conditions presenting iron deficiency as co‐morbidity, such as COPD and chronic cardiac failure.

## Materials and Methods

### Human skeletal muscle biopsies

The study enrolled patients (age > 18 years) with pancreatic cancer surgically treated at the 3rd Surgical Clinic of Padova University Hospital. Cancer patients were classified as severely cachectic in cases of > 10% weight loss in the 6 months preceding surgery.

The study also enrolled control, healthy donors undergoing elective surgery for non‐neoplastic and non‐inflammatory diseases. Patients with signs of infection were excluded. All patients joined the protocol according to the guidelines of the Declaration of Helsinki and the research project has been approved by Ethical Committee for Clinical Experimentation of Provincia di Padova (protocol number 3674/AO/15). Written informed consent was obtained from participants. From all patients a blood sample was retrieved prior to any surgical manipulation and the biopsies were performed during elective surgery within 30 min of the start of the surgery by cold section of a rectus abdominal fragment of about 0.5–1 cm. The fragment was immediately frozen and conserved in liquid nitrogen for gene expression analysis.

### Human handgrip strength

Participants with either an absolute iron deficiency (AID) or a functional iron deficiency (FID) were included in the clinical study. AID is defined by an iron‐saturation of transferrin (TSAT) < 20% and serum ferritin level < 30 ng/ml. FID is defined by a TSAT < 20% and serum ferritin levels above 30 ng/ml. Patients were treated with a single infusion of 1,000 mg of iron intravenously (ferric carboxymaltose).

The participants were asked to perform two handgrip tests to measure their strength using a hand dynamometer. The first handgrip test (HG1) was conducted prior to iron administration. The second handgrip test (HG2) was conducted within 2–12 days after the iron IV administration.

The hand dynamometer was calibrated and the measurements have an accuracy of +/− 5%. The test–retest reliability is good (*r* > 0.80) and the inter‐rater reliability is excellent (*r* = 0.98).

The handgrip test required the participants to be seated, positioning their forearm of their hand in a 90° angle with their body. The arm should not be pressed to the body or supported by an armrest and the shoulders should be relaxed. The grip of the hand dynamometer was adjusted to the hand size of the participant. The hand dynamometer was placed in the dominant hand and the participant was asked to squeeze the hand dynamometer as hard as possible until the strength indicator was stabilized (this took approximately 3–5 s). This was repeated three times, in between each measurement the participant was given 30 s to relax the arm and hand muscles.

All participants gave written informed consent to participate in the study and the study was approved by the Antwerp University Hospital ethical committee in accordance with the ethical standards established by the 1964 Declaration of Helsinki.

### Animal experimentation

All animal experiments were authorized by the Italian Ministry of Health and carried out according to the European Community guiding principles in the care and use of animals. The BaF experiments performed in Belgium were approved by and performed in accordance with the guidelines of the local ethics committee from the UCLouvain, Belgium. Housing conditions were as specified by the Belgian Law of 29 May 2013, regarding the protection of laboratory animals. In all experiments, female littermates of 8–10 weeks were assigned randomly to experimental groups. Cancer cell lines (C26 colon murine adenocarcinoma and LLC Lewis lung carcinoma, 1 × 10^6^ cells/mouse) were injected subcutaneously in the flank of 8‐weeks‐old female mice (BALB/C for C26, C57 BL/6 for LLC). Baf3 cachexia was induced as previously reported (Bindels *et al,* 2012), injecting Bcr‐Abl‐transfected Baf3 intravenously in 6‐weeks‐old Balb/C mice. LLC (Lewis Lung Carcinoma) tumor‐bearing mice were necropsied at day 24. For C26, to compare the various treatment all mice were necropsied at day 12, except for the survival experiment. Electroporation experiments were performed as previously described (Sartori *et al,* 2021) on tibialis anterior muscle with pHuji‐TFR1 plasmid (Addgene 61505) or shTFR‐GFP plasmid using BLOCK‐iT PolII miR RNAi expression kit (Thermo Fisher K493600).

Ferric carboxymaltose (Ferrinject 15 mg/kg, Vifor Pharma) or saline solution (NaCl 0.9%) was injected in the tail vein every 5 days starting from day 5 post‐C26 inoculation. Blood was collected by cardiac puncture, and perfusion with PBS after anesthesia was performed to obtain samples for iron quantification. For the phlebotomy experiment, retroorbital bleeding (400 µl of blood) was performed under anesthesia, and mice were fed with iron‐deprived diet (Mucedola) for 11 days before sacrifice. To measure strength, mice were held by the middle part of the tail and allowed to grab the metal grid of a dynamometer (2Biol) in a parallel position before being gently pulled backward. The maximal force generated by the grip was recorded, and the measure was repeated six times. To quantify muscle wasting, gastrocnemii and quadriceps were freshly isolated, weighted, and normalized to the respective tibial length.

### Cell culture and *in vitro* treatments

C2C12 myoblasts were purchased from ATCC and cultured in DMEM with 10% fetal bovine serum (FBS). After reaching full confluency, differentiation was induced by switching to 2% horse serum (HS) DMEM for 4 days. Human myoblast cell line, originated from the quadriceps of a 38‐year‐old male donor. The cells were cultured in Skeletal Muscle Cell Basal Medium (PromoCell, Heidelberg, Germany) containing 5% FBS and supplemented with hbFGF, hEGF, fetuin, insulin, and dexamethasone. Differentiation was induced by switching to DMEM containing 10 µg/ml insulin, gentamicin 1% (Gibco) for 7 days.

Conditioned medium (CM) was prepared as previously described (Wyart *et al,* 2018). Briefly, cancer cells were grown to high confluency, then conditioned in serum‐free DMEM for 24 h, medium was harvested and centrifuged at 500 *g* for 10 min. Supernatant was collected and used as CM at 10% final concentration. Deferoxamine (DFO, Sigma D9533) and bathophenanthroline disulfonic acid (BPS, Sigma 146617) were used at 100 µM. Apotransferrin (Sigma T0178), Hinokitiol (HNK, Sigma 469521), and ferric citrate (Sigma F3388) were used at 400 µg/ml, 5 µM, and 250 nM, respectively. Activin A (RnD Sytem 338‐AC) and Dexamethasone (DEXA, Sigma D4902) were used at 1 nM and 1 µM, respectively. Rotenone was used at 20 nM. Myotubes were treated for 48 h for all compounds. For cell transfection, C2C12 myoblasts were differentiated for 3 days prior to transfection with esiTFR1, esiNCOA4, or esiGFP (250 ng/ml) using Lipofectamine 2000 (Invitrogen 11668019). Myotubes were photographed and lysed at 72 h post‐transfection. For myotube diameter quantification, pictures of myotubes were taken with phase contrast microscopy (Zeiss) at 20× magnification, and myotube diameter was measured using the software JMicroVision as previously described (Murata *et al,* 2018).

### Western Blotting

Frozen gastrocnemius samples were lysed in RIPA lysis buffer (150 mM of NaCl, 50 mM of Tris‐HCl, 0.5% sodium deoxycholate, 1% Triton X‐100, 0.1% SDS, and 1 mM of EDTA) supplemented with protease and phosphatase inhibitor cocktail (Roche). Protein concentration was determined using BCA assay (Thermo Fisher Scientific). Fifteen or thirty micrograms of protein from cell or gastrocnemius lysates, respectively, were loaded per well on Mini‐Protean TGX Stain‐Free precast polyacrylamide gels (Bio‐rad) for SDS‐PAGE. Stain‐Free imaging was performed using Chemidoc MP imager in order to visualize total protein patterns. Proteins were then transferred onto PVDF membranes prior to immunoblotting analysis. Blots were probed with the following primary antibodies: P‐Thr172‐AMPK (Cell Signaling 2535), Total‐AMPK (Cell Signaling 2532), Ferritin (Sigma F5012), IRP2(PA‐116544), Transferrin Receptor 1 (Santa Cruz sc65882), NCOA4 (Santa Cruz C‐4), OxPhos cocktail (Thermofisher 8199), Ferroportin (Novus NBP1‐21502), Vinculin (Cell Signaling 4650). Protein carbonylation was assessed by measuring the levels of carbonyl groups using the OxyBlot protein oxidation detection kit (Sigma‐Aldrich S7150). Quantification analysis of blots was performed with Image Lab software (BioRad).

### Iron quantification and Heme assay

Iron content in skeletal muscle was quantified by ICP‐MS (Element‐2; Thermo‐Finnigan, Rodano, Italy) using medium mass resolution (M/∆M ~4,000). Fifty to hundred milligrams of freshly excised and snap‐frozen quadriceps were submitted to overnight dialysis. Samples were collected before and after dialysis to assess total and protein‐bound iron, respectively. Additionally, iron content was also measured in isolated mitochondria from quadriceps. All samples were digested overnight in 0.5 ml of concentrated HNO_3_ (70%) and mineralized by microwave heating for 6 min at 150°C (Milestone, Ethos Up Microwave Digestion System). A natural abundance iron standard solution was analyzed in parallel in order to check for changes in the systematic bias. The calibration curve was obtained using four iron standard solutions (Sigma‐Aldrich) in the range of 0.2–0.005 μg/ml. For liver and spleen iron, samples were heated at 180°C overnight and mineralized in 10 ml of HCl 3 M/ 10% trichloroacetic acid per gram of dry tissue overnight at 65°C with gentle shaking. 10 µl of supernatants were mixed with a solution of 1.7% of thioglycolic acid (TGA), 84.7% of sodium acetate acetic acid pH 4.5, 13.6% of BPS (Sigma 146617). After 1 h of incubation at 37°C, absorbance was measured at 535 nm. Iron content was determined using a standard curve of ferrous ammonium sulfate. Heme concentration was determined by fluorescence assay as previously described (Sinclair *et al,* 2001). Saturated oxalic acid solution was added to 40 µg of proteins from gastrocnemius lysates prior to heating at 95°C for 30 min. Samples were loaded in triplicates and fluorescence was measured at 400 nm excitation and 662 nm emission wavelengths.

Intracellular labile iron pool was measured as previously described with modifications (Schoenfeld *et al,* 2017). Briefly, C2C12 cells were treated with 500 nM Calcein AM (Sigma 56496) for 15 min and fluorescence intensity was measured using a microplate reader. Cells were then incubated for 15 min with 100 µm of 2′,2′‐Bipyridyl (BIP) prior to the second measurement of fluorescence. The LIP was calculated as following : LIP (A.U) = MFI after BIP – MFI before BIP. The obtained value were normalized on DAPI fluorescence intensity.

### Mitochondria isolation and metabolic assays

Mitochondria were isolated from snap‐frozen quadriceps by Mitocheck Mitochondrial Isolation kit (Cayman chemical 701010). ATP content was quantified in 20 μg of fresh isolated mitochondria by CellTiter‐Glo^®^ Luminescent Cell Viability Assay (Promega G7570). Aconitase activity was measured in quadriceps homogenates by enzymatic assay (Cayman Chemical 705502). Oxygen Consumption Rate (OCR) measurements were conducted using a Seahorse XFe96 analyzer according to manufacturer’s protocol. C2C12 cells were directly differentiated in XFe96 cell culture plates and treated with 10% C26 CM, ferric citrate 250 nM, or the combination of both for 48 h and incubated in 5% CO_2_ at 37°C. One hour prior to analysis, growth medium was replaced with assay medium (DMEM without phenol red and sodium bicarbonate (Corning 90‐013‐PB) that was supplemented with 1 mM of pyruvate, 2 mM of l‐glutamine, and 10 mM of glucose, pH 7.4) and incubated in a non‐CO_2_ incubator. During assay, 1 μM of oligomycin (Sigma 495455), 1 μM of FCCP (Sigma C2920), and 0.5 μM of rotenone/antimycin A (Sigma R8875 and A8674) were sequentially injected into each well in accordance with standard protocols. Absolute rates (p moles/min) were normalized to μg of protein determined by Bradford Assay (BioRad 5000006).

### Histology

Extracted gastrocnemii were immediately frozen in isopentane cooled in liquid nitrogen and stored at −80°C. Transversal sections of 5 µm thickness were cut at the midbelly with a cryostat. Sections were fixed for 10 min in PFA 4%, then blocked with 0.1% triton x‐100, 1% BSA in PBS before incubating with primary antibodies against fast/slow isoforms of myosin heavy chain and laminin (Abcam 91506, Abcam M8421, Santa Cruz 59854), followed by incubation with the corresponding secondary antibodies (Alexa‐488, Alexa‐568). For the electroporation of tibialis anterior with reporter plasmids, 8 μm cryosections were stained with AlexaFluor 555‐conjugated Wheat Germ Agglutinin (WGA, Thermo Fisher Scientific W32464) and DAPI. Pictures were taken with a fluorescent microscope and fiber areas were measured with ImageJ software (more than 500 fibers were analyzed per animal). The enzymatic activity of succinate dehydrogenase (SDH) was assessed on cryosections using the Succinic Dehydrogenase Stain (Bio‐Optica, 30–30114LY) according to manufacturer’s instructions.

### RNA isolation and quantitative PCR

Total RNA was isolated from snap‐frozen tissue samples using TRIzol reagent (Invitrogen 15596026) according to the manufacturer’s guidelines. 1 µg of total RNA was reverse‐transcribed using the High Capacity cDNA Reverse Transcriptase kit (Applied Biosystems 4374966). cDNA was analyzed by Real Time Quantitative PCR (ABI PRISM 7900HT FAST Real‐Time PCR system, Applied Biosystems) using the Luna Universal Probe qPCR master mix (NEB M3004) and the Universal Probe Library system (Roche Applied Science), or with SYBR Green master mix (Applied Biosystems A25741). Relative mRNA levels were calculated using the 2‐ΔΔCT method and normalized to GAPDH or 18s mRNA (Eukaryotic 18s rRNA Endogenous Control, Thermo Fisher 4310893E), respectively. For human skeletal muscle biopsies, 500 ng of RNA was reverse transcribed using SuperScript IV Reverse Transcriptase (Thermo Fisher 18090010). Human data were all normalized to Actb gene expression. The following primers were used:

**Table:** 

Gene	Forward sequence (5′–3′)	Reverse sequence (5′–3′)
Human TFR1	aggaaccgagtctccagtga	atcaactatgatcaccgagt
Human ACTB	gggaaatcgtgcgtgaca	ggactccatgcccagga
mTFR1	tcctttccttgcatattctgg	ccaaataaggatagtctgcatcc
mMFRN2	tgtgtggcgacattacttcat	gcatcctctgcttgacgact
mALAS2	ctcaccgtctttggttcgtc	ggacaggaccgtagcaacat
mATRO1	agtgaggaccggctactgtg	gatcaaacgcttgcgaatct
mMURF1	tgacatctacaagcaggagtgc	tcgtcttcgtgttccttgc
mREDD1	ccagagaagagggccttga	ccatccaggtatgaggagtctt
mCPT1B	aagagaccccgtagccatcat	gacccaaaacagtatcccaatca
mACOT1	caactacgatgacctcccca	gagccattgatgaccacagc
mMCD	gcacgtccgggaaatgaac	gcctcacactcgctgatctt
mGAPDH	aggtcggtgtgaacggatttg	tgtagaccatgtagttgaggtca

### RNA electrophoretic mobility shift assay (REMSA)

The fractionation method described by Rothermel *et al* (2000) was adopted with minor modification to extract the cytosolic protein fractions from gastrocnemius muscle. Briefly, after removal of connective tissues, muscle samples were homogenized for 1 min in ice‐cold lysis buffer (25 mM of Hepes pH 8, 5 mM of KCl, 0.5 mM of MgCl_2_, 1% NP‐40, 1× protease inhibitors) using a tight‐fitting Teflon pestle attached to a Potter S homogenizer (Sartorius Stedium) set to 1,000 rpm. Following centrifugation at 800 *g* for 15 min at 4°C to pellet the nuclei and cell debris, the supernatants were collected, subjected to further centrifugation three times at 500 *g* for 15 min at 4°C to remove residual nuclei and used for the REMSA as nuclei‐free total cytosolic protein fractions. The IRP‐IRE interactions were performed using the LightShift Chemiluminescent RNA Electrophoretic mobility shift assay kit (REMSA, Thermo Fisher 20148). Briefly, 20 μl of reaction containing nuclease‐free water, 5×REMSA Binding Buffer (50 mM of Tris‐HCl pH 8.0, 750 mM of KCl, 0.5% Triton‐X 100, 62.5% glycerol), tRNA (Thermo Fisher 20158), 6 μg of muscle cytosolic extracts, biotinylated and unlabeled ferritin probe 5′–UCCUGCUUCAACAGUGCUUGGACGGAAC–3′ and where indicated, 0.5 mM of EDTA and 1 mM of DTT, were incubated for 30 min at room temperature. Afterward, the samples were carefully mixed with 5 μl of 5×REMSA Loading Buffer and resolved on 6% polyacrylamide gel and transferred on to nylon membrane (Roche Diagnostics, Indianapolis, IN). After the membrane was cross linked with UV‐light, the IRP‐IRE complexes were visualized by the chemiluminescent nucleic acid detection module (Thermo Fisher 20158).

### mtDNA copy number analysis

Briefly, DNA was isolated from C2C12 myotubes using phenol–chloroform method as reported in (Quiros *et al,* 2017). Approximately 50 ng total DNA was used for quantitative analysis of mtDNA level by qPCR using SYBR Green master mix. The relative copy number of mtDNA was determined using mitochondrial‐specific primers for ND1 and 16S and nuclear specific Hexokinase 2 (HK2):

**Table:** 

Gene	Forward sequence (5′–3′)	Reverse sequence (3′–5′)
16S rRNA	CCGCAAGGGAAAGATGAAAGAC	TCGTTTGGTTTCGGGGTTTC
ND1	CTAGCAGAAACAAACCGGGC	CCGGCTGCGTATTCTACGTT
HK2	GCCAGCCTCTCCTGATTTTAGTGT	GGGAACACAAAAGACCTCTTCTGG

Quantitative analysis was carried out using the following reaction conditions; the pre‐amplification step was carried out at 95°C for 3 min, followed by 40 cycles at 95°C for 10 s, annealing was performed at 58°C for 30 s and final extension was carried out at 72°C for 30 s. After obtaining the Ct values, the mtDNA copy number was calculated using the following formula: ΔCt = Ct(mtDNA gene)−Ct(nDNA gene).

### Fusion index

After 48 h treatment with C26CM and FeCM as described previously, cells were fixed in 4% PFA, permeabilized and stained for Myosin Heavy Chain (Sigma M4276) and nuclei were stained with DAPI. The fusion index was determined as followed : (fusion%) = (number of nuclei per myotube)/(total number of nuclei in the field. At least 6 random fields (10× magnification) were analyzed per condition using the ImageJ Software.

### Quantification and statistical analysis

All graphs show mean ± SEM. N represents the total number of independent experiments. Statistical significance was tested as indicated in figure legends with GraphPad Prism (version 6.0, GraphPad Software). Statistical significance was tested using two‐tailed Student’s *t*‐test for comparisons of two groups, one‐way Anova followed by Bonferroni correction for comparisons of multiple groups with one variable, or two‐way Anova followed by Tukey’s multiple comparison test. Significance of survival rate difference was determined by Chi‐square test. Non‐parametric Mann–Whitney test was used for human grip strength data. Significance was defined as *P < 0.05, **P < 0.01, and ***P < 0.001.

## Author contributions

**Elisabeth Wyart:** Conceptualization; Data curation; Investigation; Writing – original draft. **Myriam Hsu:** Investigation; Methodology equal); Writing – original draft; Writing – review & editing. **Roberta Sartori:** Methodology. **Erica Mina:** Investigation. **Valentina Rausch:** Methodology. **Elisa Sefora Pierobon:** Resources. **Mariarosa Mezzanotte:** Methodology. **Camilla Pezzini:** Methodology. **Laure Bindels:** Resources; Formal analysis. **Andrea Lauria:** Software. **Fabio Penna:** Investigation. **Emilio Hirsch:** Formal analysis. **Miriam Martini:** Data curation; Formal analysis; Writing – review & editing. **Massimiliano Mazzone:** Conceptualization equal). **Antonella Roetto:** Investigation; Methodology. **Simonetta Geninatti Crich:** Investigation; Methodology. **Hans Prenen:** Conceptualization; Formal analysis. **Marco Sandri:** Data curation; Visualization. **Alessio Menga:** Project administration; Writing – review & editing. **Paolo Ettore Porporato:** Conceptualization; Supervision; Funding acquisition; Investigation; Writing – original draft; Project administration; Writing – review & editing.

## Disclosure and competing interests statement

The authors declare that they have no conflict of interest.

## Supporting information



Expanded View Figures PDF

Table EV1

Movie EV1

Source Data for Figure 1

Source Data for Figure 2

Source Data for Figure 3

Source Data for Figure 4

Source Data for Figure 5

Source Data for Figure 6

## Data Availability

No data requiring public database deposition have been generated.

## Footnotes

^§^
Correction added on 5 April 2022, after first online publication: “3” has been changed to “4”.
