# Supplementary material for: Iron supplementation is sufficient to rescue skeletal muscle mass and function in cancer cachexia
Source: EMBO Rep. 2022 Feb 24;23(4):e53746. doi: 10.15252/embr.202153746 (PMC8982578; doi:10.15252/embr.202153746)
Supplement: Supplementary file 1 — Expanded View Figures PDF [file EMBR-23-e53746-s009.pdf]

Expanded View Figures

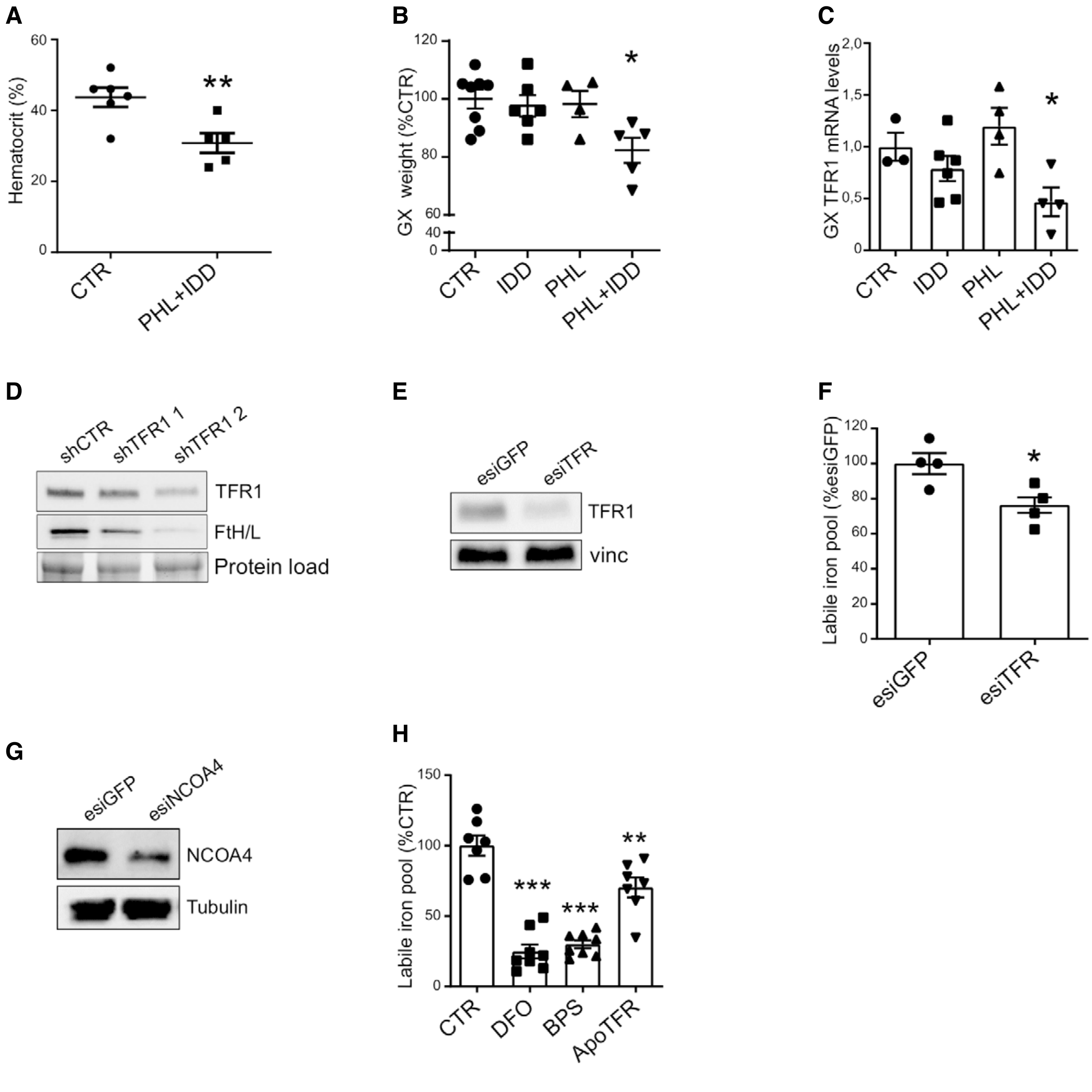

Figure EV1. Iron deficiency induces skeletal muscle atrophy.

- A Hematocrit in mice subjected to iron deprivation by a combination of iron-deficient diet (IDD) and phlebotomy (PHL) (*n* = 5–6).
- B Gastrocnemius weight in mice subjected to iron deprivation by iron-deficient diet (IDD), phlebotomy (PHL), or a combination of both (*n* = 4–8).
- C Gastrocnemius TFR1 mRNA levels in mice after iron deprivation by iron-deficient diet (IDD), phlebotomy (PHL) or a combination of both (*n* = 3–6).
- D Representative Western blot of TFR1 and ferritin after transfection with shTFR1-pGFP in 3T3 cells (*n* = 3).
- E Representative Western blot of TFR1 after knockdown in C2C12 myotubes (*n* = 3).
- F Labile iron pool in C2C12 myotubes after TFR1 knockdown (*n* = 3).
- G Representative Western blot of NCOA4 after knockdown in C2C12 myotubes (*n* = 3).
- H Labile iron pool in C2C12 myotubes treated with iron chelators DFO, BPS, or apo-transferrin (*n* = 3).

Data information: For all data, *n* represents the number of biological replicates. Statistical significance was calculated by unpaired, two-tailed Student's *t*-test (A, F) or one-way Anova with Bonferroni's correction (B, C, and H). Data are mean ± SEM. *$P < 0.05$, **$P < 0.01$, ***$P < 0.001$.

© 2022 The Authors. Published under the terms of the CC BY 4.0 license

Figure EV2. Altered iron metabolism in the skeletal muscle is a feature of cancer-induced cachexia.

A Hematocrit levels of C26 tumor-bearing mice at day 12 post C26 injection (n = 3–6).
B Body weight evolution of mice after C26 injection (n = 6).
C Quadriceps weight of C26 tumor-bearing mice normalized to tibial lenght (n = 6).
D Liver TFR1 mRNA levels normalized to 18s in C26 tumor-bearing mice (n = 5).
E Total liver iron content in C26 tumor-bearing mice (n = 11).
F, G Total body weight (F) and gastrocnemius weight (G) in LLC tumor-bearing mice (n = 5–6).
H TFR1 mRNA levels normalized to 18s in the gastrocnemius of LLC tumor-bearing mice (n = 3).
I, J Total body weight gain (I) and gastrocnemius weight (J) of BaF-transplanted mice (n = 6–8).
K TFR1 mRNA levels normalized to 18s in the gastrocnemius of BaF-transplanted mice (n = 6–8).
L Raw blots of Fig 2F RNA electrophoretic mobility shift assay (REMSA). The biotin-labeled IRE probe was incubated without (lane 1) or with cytosolic gastrocnemius extracts from CTR and C26 tumor-bearing mice, in native (lane 2) or reducing conditions (lane 4). Where indicated, unlabeled IRE probe was added in 200-fold molar excess (lanes 3 and 5) (n = 3).

Data information: For all data, n represents the number of biological replicates. Statistical significance was calculated by unpaired, two-tailed Student's t-test (A, C-K) or ordinary two-way Anova (B). Data are mean ± SEM. *$P < 0.05$, **$P < 0.01$, ***$P < 0.001$.

 © 2022 The Authors. Published under the terms of the CC BY 4.0 license

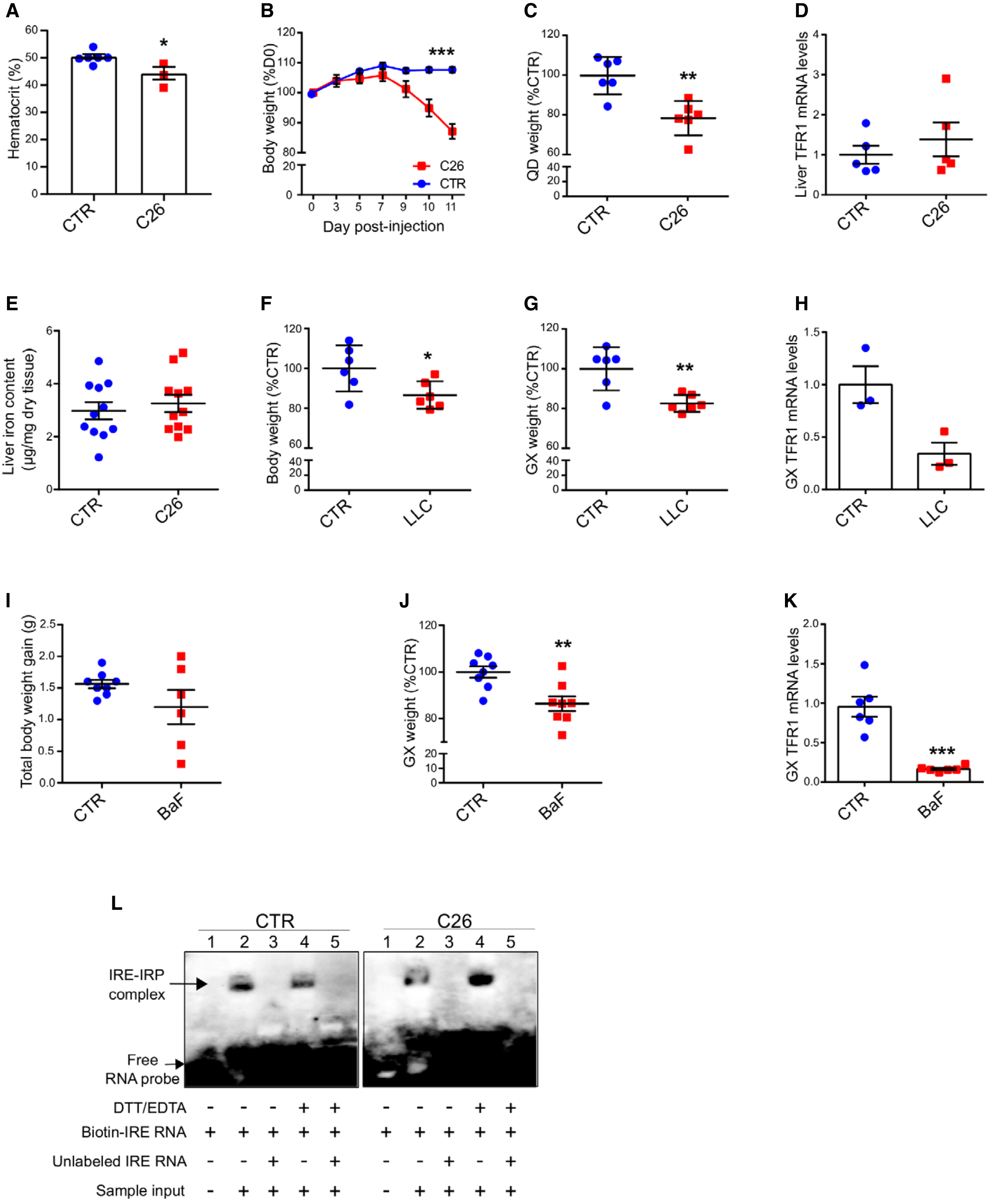
A
Hematocrit (%)
CTR
C26
B
Body weight (%D0)
Day post-injection
C26
CTR
C
QD weight (%CTR)
D
Liver TFR1 mRNA levels
E
Liver iron content (µg/mg dry tissue)
F
Body weight (%CTR)
LLC
G
GX weight (%CTR)
H
GX TFR1 mRNA levels
I
Total body weight gain (g)
BaF
J
GX weight (%CTR)
K
GX TFR1 mRNA levels
L
IRE-IRP complex
Free RNA probe
DTT/EDTA
Biotin-IRE RNA
Unlabeled IRE RNA
Sample input

Figure EV2.

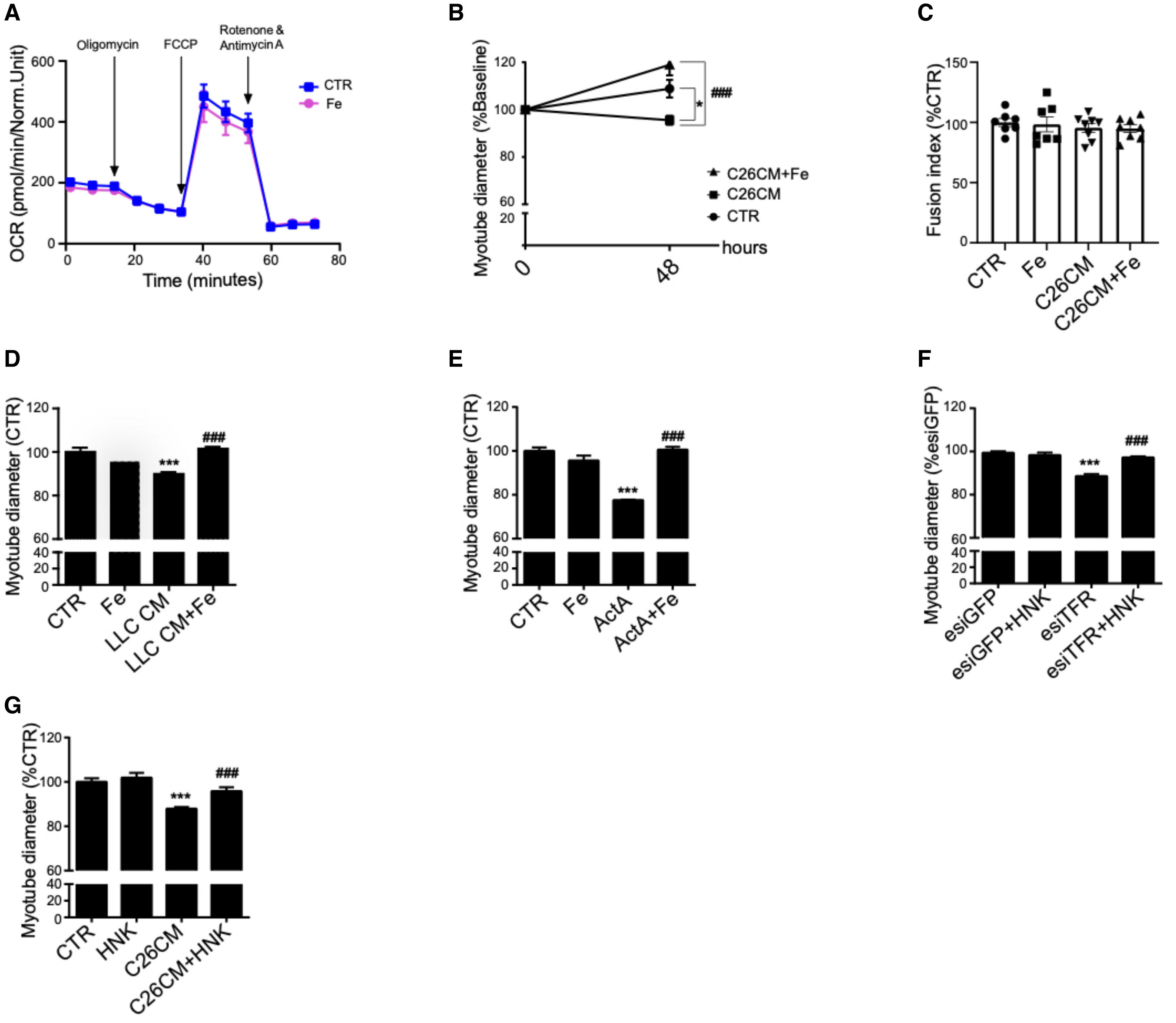

Figure EV3. Iron enhances mitochondrial function and prevents cancer-induced myotube atrophy.

A Profile of oxygen consumption rate OCR in C2C12 myotubes after 48 h treatment with ferric citrate (n = 6).
B Myotube diameter normalized to Day 0 values (n = 3).
C Fusion index of C2C12 myotubes treated with C26 CM and ferric citrate for 48 h (n = 7–8).
D Diameter of C2C12 myotubes treated with LLC CM and iron citrate for 48 h (n = 3).
E Diameter of C2C12 myotubes treated with Activin A (ActA) and ferric citrate for 48 h (n = 3).
F Diameter of TFR1-silenced C2C12 myotubes after 24 h treatment with iron ionophore hinokitiol (HNK) (n = 3).
G Diameter of C2C12 myotubes treated with C26 CM and HNK for 48 h (n = 3).

Data information: For all data, n represents the number of biological replicates. Statistical significance was calculated by one-way Anova with Bonferroni's correction (A-E). Data are mean ± SEM. ***P < 0.001 compared to control and ###P < 0.001 compared to conditioned medium, ActA, or esiTFR-treated group.

 © 2022 The Authors. Published under the terms of the CC BY 4.0 license

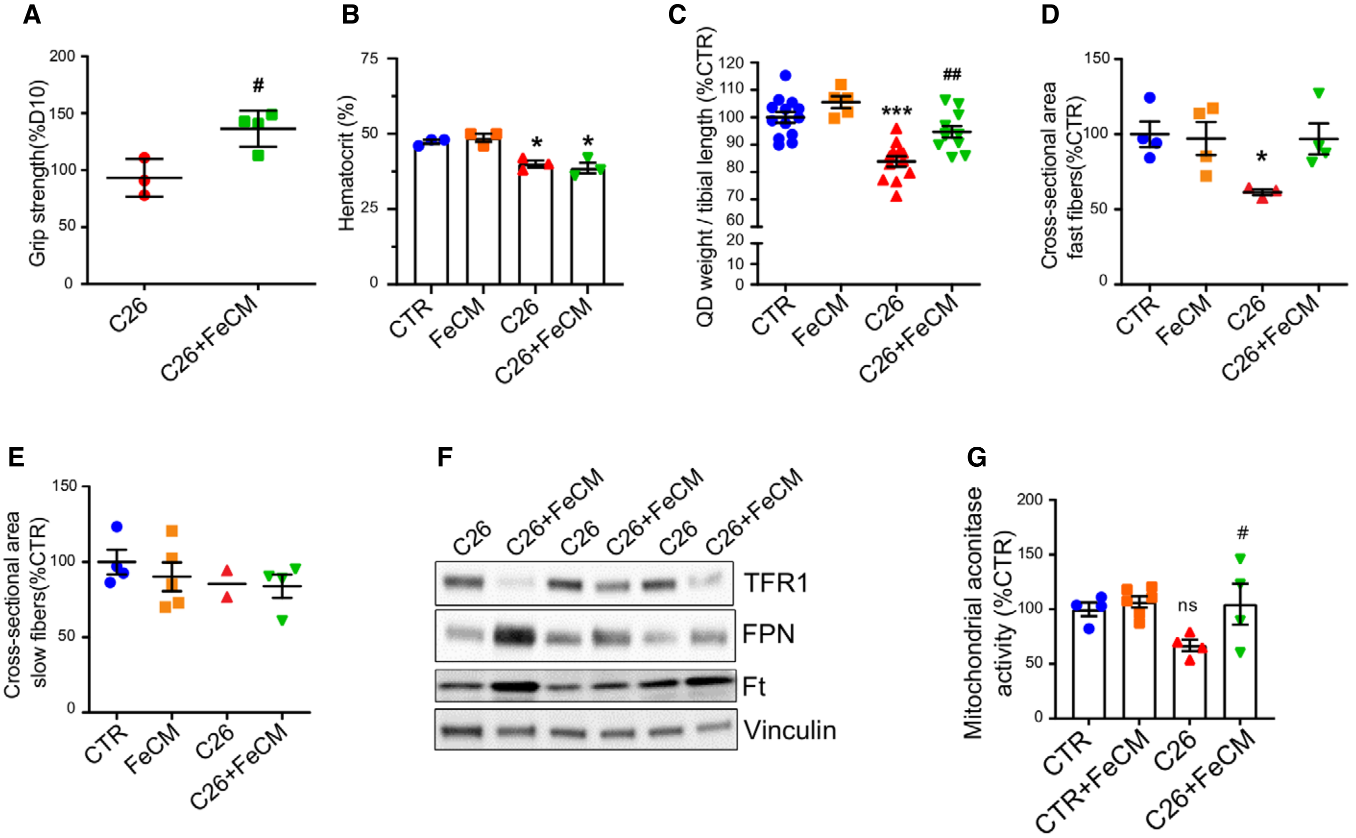

Figure EV4. Iron supplementation prevents cancer-induced cachexia.

A Grip strength of C26 tumor-bearing mice measured at day 11 and normalized to day 10 (n = 3–4).
B Hematocrit in tumor-bearing mice measured on day 12 post C26 injection (n = 3)
C Quadriceps weight normalized to tibial length of C26 tumor-bearing mice at day 12 post C26 injection (n = 5–12).
D, E Average cross-sectional area of fast (D) and slow (E)–twitch muscle fibers (n = 2–5).
F Representative Western blot of iron metabolism proteins in tumor extracts from C26 tumor-bearing mice showing a physiological response to iron loading (n = 3).
G Mitochondrial aconitase activity in quadriceps of C26 tumor-bearing mice supplemented with FeCM (n = 4–6).

Data information: For all data, n represents the number of biological replicates. Statistical significance was calculated by unpaired, two-tailed Student's t-test (A) or one-way Anova with Bonferroni's correction (B-D). Data are mean ± SEM. *$P < 0.05$, ***$P < 0.001$ compared to control and #$P < 0.05$, ##$P < 0.01$compared to C26-untreated group.

© 2022 The Authors. Published under the terms of the CC BY 4.0 license
