# Supplementary material for: Iron supplementation is sufficient to rescue skeletal muscle mass and function in cancer cachexia
Source: EMBO Rep. 2022 Feb 24;23(4):e53746. doi: 10.15252/embr.202153746 (PMC8982578; doi:10.15252/embr.202153746)
Supplement: Supplementary file 2 — Table EV1 [file EMBR-23-e53746-s008.docx]

**Table EV1**

| **Subject** | 1 | 2 | 3 | 4 | 5 | 6 | 7 |
| --- | --- | --- | --- | --- | --- | --- | --- |
| **Sex** | M | F | F | M | M | F | M |
| **Age (year)** | 70 | 60 | 50 | 80 | 69 | 64 | 59 |
| **Tumor type** | Pancreatic Cancer | Melanoma | Breast Cancer | Prostate Cancer | HCC | Oropharynx Cancer | Colon Cancer |
| **Height**  **(cm)** | 170 | 170 | 169 | 164 | 174 | 163 | 184 |
| **BW (kg)** | 67.1 | 74.4 | 92.1 | 73.3 | 92.2 | 58.1 | 132 |
| **Weight Loss (%)** | 18.46 | -2.9 | -0.98 | 4.8 | 5.62 | 4.28 | 3.86 |
| **Hemoglobin**  **(g/dL)** | 8.2 | 9.7 | 10.3 | 9.7 | 13.4 | 9.8 | 11.9 |
| **CRP (mg/L)** | <2.9 | 69.2 | 14.2 | <4 | 34.2 | 22.2 | 4 |
| **Ferritin (µg/L)** | 821 | 209 | 20 | 837 | N.D. | 585 | 18 |
| **Transferrin saturation (%)** | 18 | 8 | 9 | 19 | 17 | 16 | 6 |
| **Dominant hand initial strength** | 32 | 24 | 32 | 32 | 26 | 16 | 30 |
| **Dominant hand final strength (kg)** | 32 | 26 | 34 | 34 | 28 | 18 | 42 |
| **Non-dominant hand initial strength (kg)** | 32 | 22 | 28 | 32 | 22 | 18 | 24 |
| **Non-dominant hand final strength (kg)** | 28 | 26 | 28 | 34 | 28 | 18 | 30 |
| **Days after iron injection** | 4 | 6 | 4 | 14 | 24 | 7 | 7 |
