# Supplementary material for: Iron supplementation is sufficient to rescue skeletal muscle mass and function in cancer cachexia
Source: EMBO Rep. 2022 Feb 24;23(4):e53746. doi: 10.15252/embr.202153746 (PMC8982578; doi:10.15252/embr.202153746)
Supplement: Supplementary file 3 — Movie EV1 [file EMBR-23-e53746-s003.zip › EMBOR-2021-53746V3-Movie_EV1/MovieEV1_legend.docx]

Movie EV1: Condition of C26 tumor-bearing mice receiving repeated administration 12 days after subcutaneous

C26 injection. vehicle (left) or ferric carboxymaltose (right).
