# Supplementary material for: Iron supplementation is sufficient to rescue skeletal muscle mass and function in cancer cachexia
Source: EMBO Rep. 2022 Feb 24;23(4):e53746. doi: 10.15252/embr.202153746 (PMC8982578; doi:10.15252/embr.202153746)
Supplement: Supplementary file 4 — Source Data for Figure 1 [file EMBR-23-e53746-s010.pptx]

## Slide 1
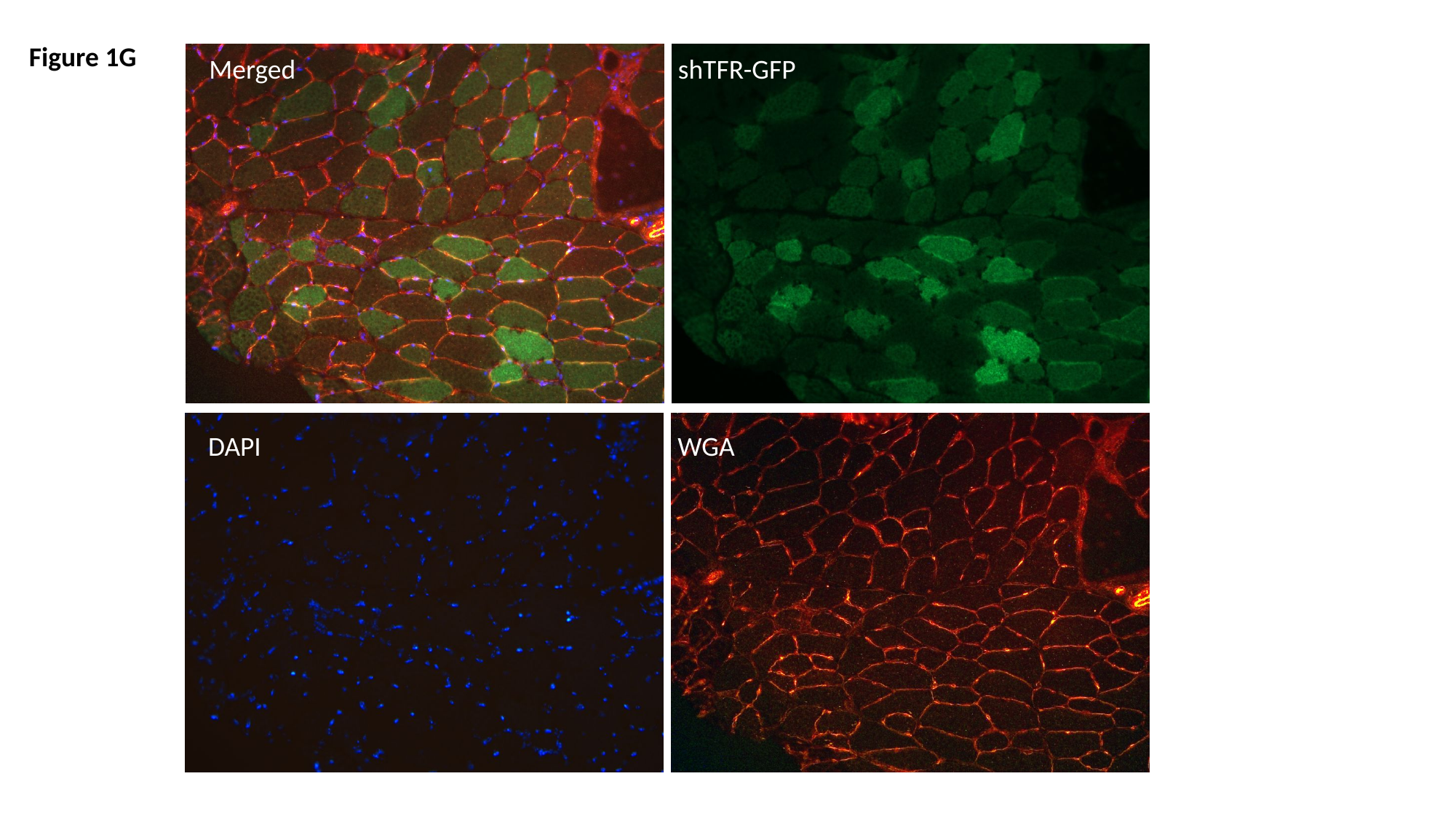

Figure 1G
Merged
shTFR-GFP
DAPI
WGA

## Slide 2
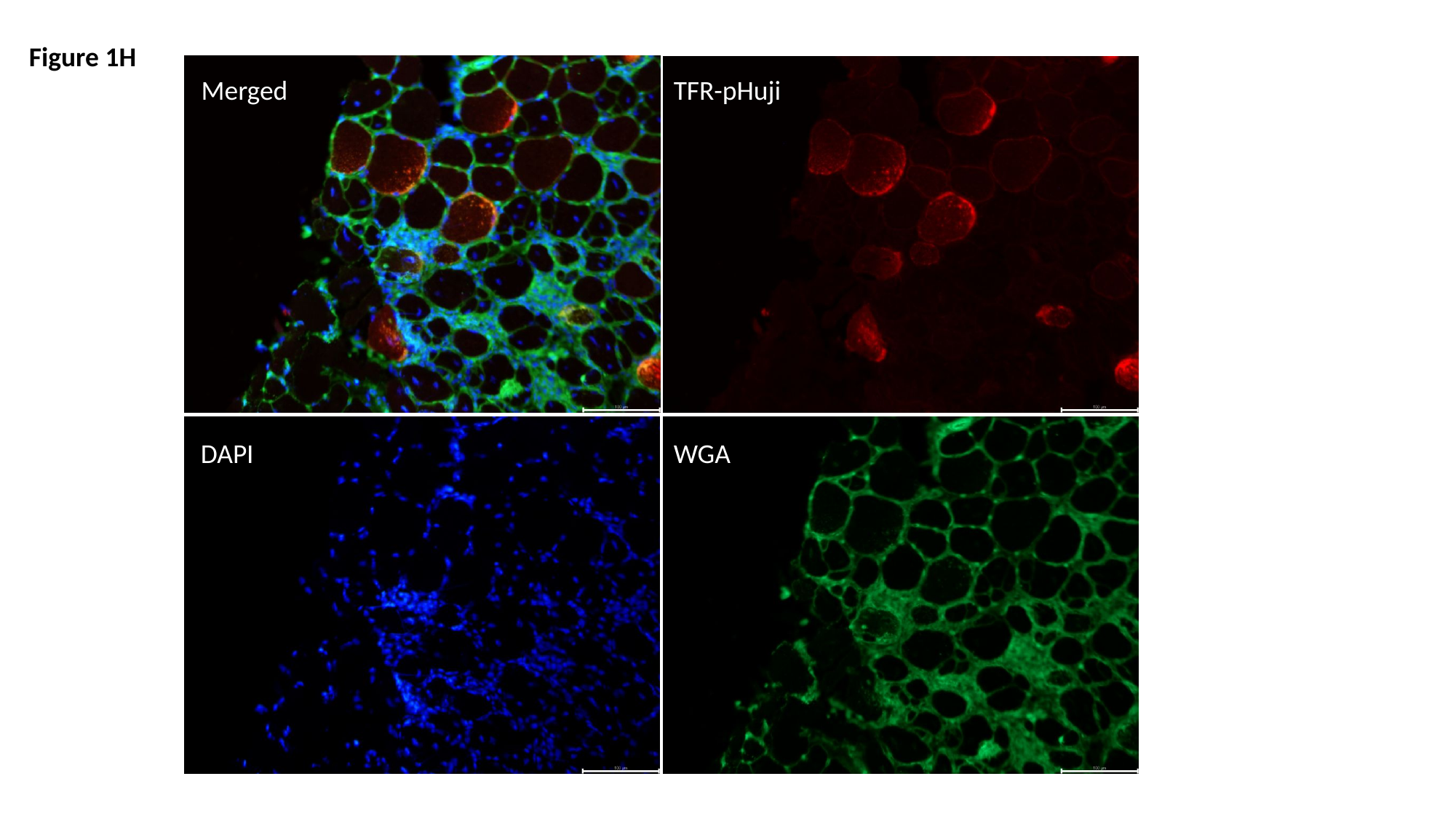

Figure 1H
Merged
TFR-pHuji
DAPI
WGA

## Slide 3
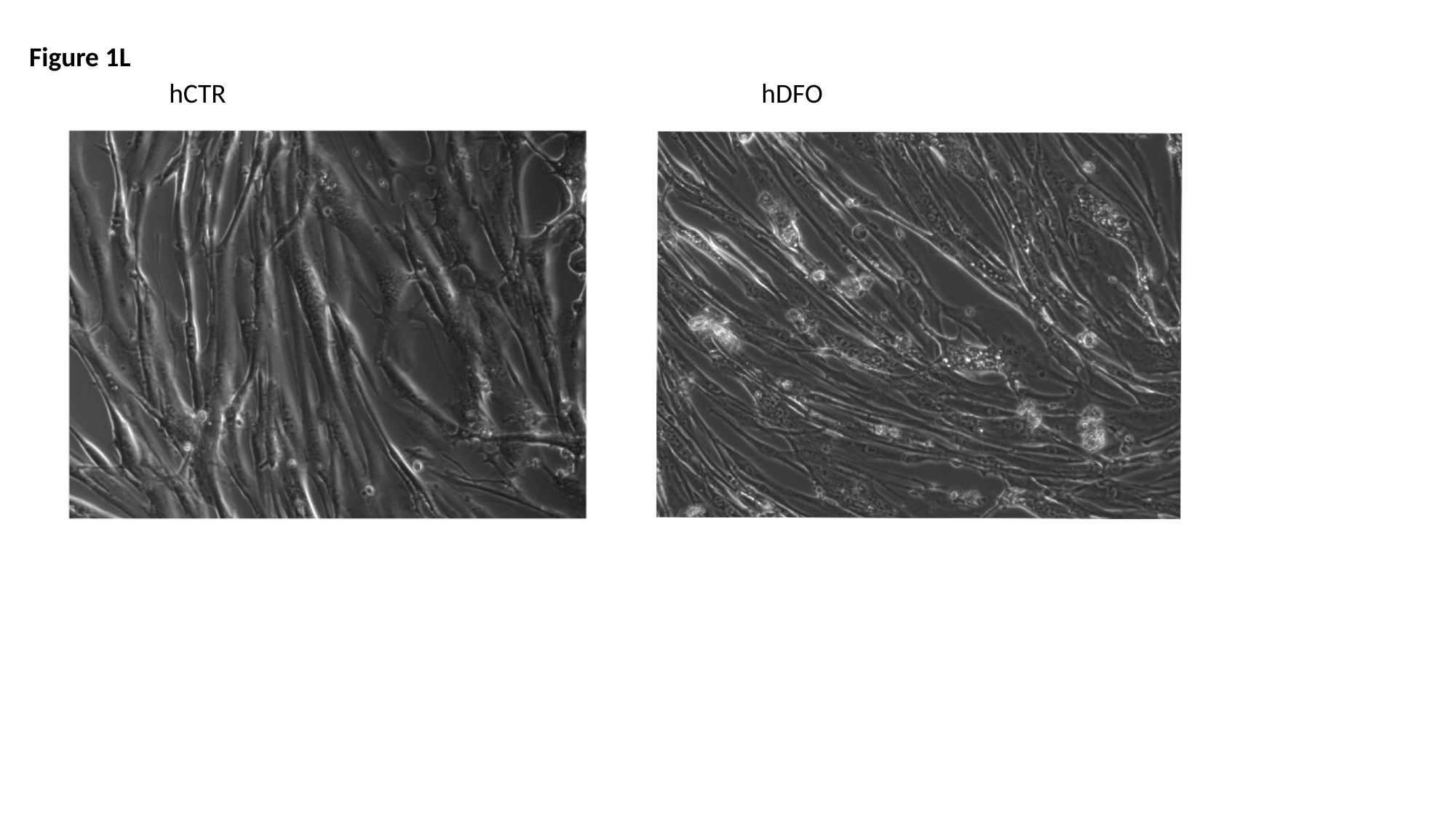

Figure 1L
hCTR
hDFO
