# Supplementary material for: Iron supplementation is sufficient to rescue skeletal muscle mass and function in cancer cachexia
Source: EMBO Rep. 2022 Feb 24;23(4):e53746. doi: 10.15252/embr.202153746 (PMC8982578; doi:10.15252/embr.202153746)
Supplement: Supplementary file 5 — Source Data for Figure 2 [file EMBR-23-e53746-s002.pptx]

## Slide 1
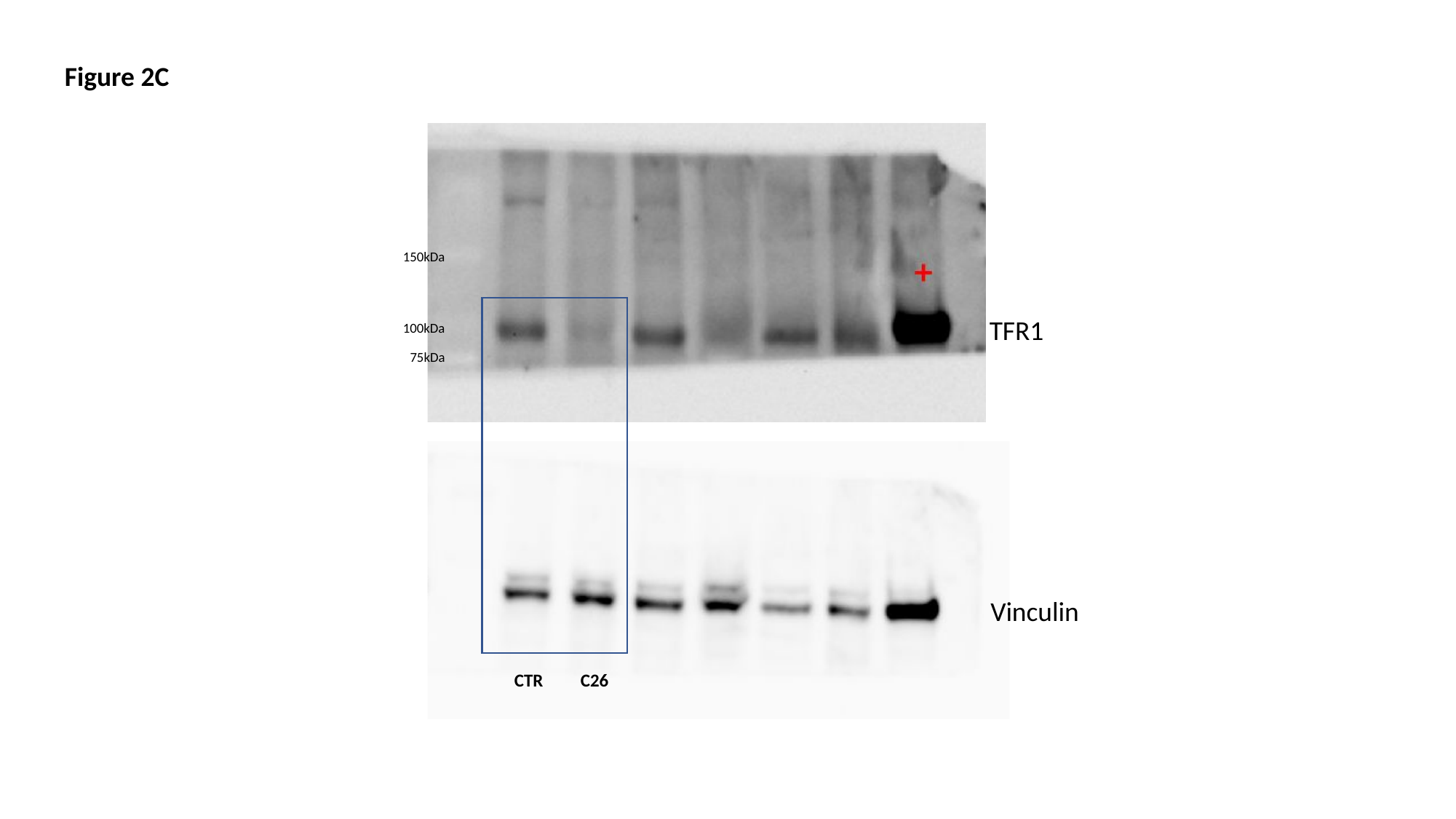

Figure 2C
150kDa
TFR1
100kDa
75kDa
Vinculin
C26
CTR

## Slide 2
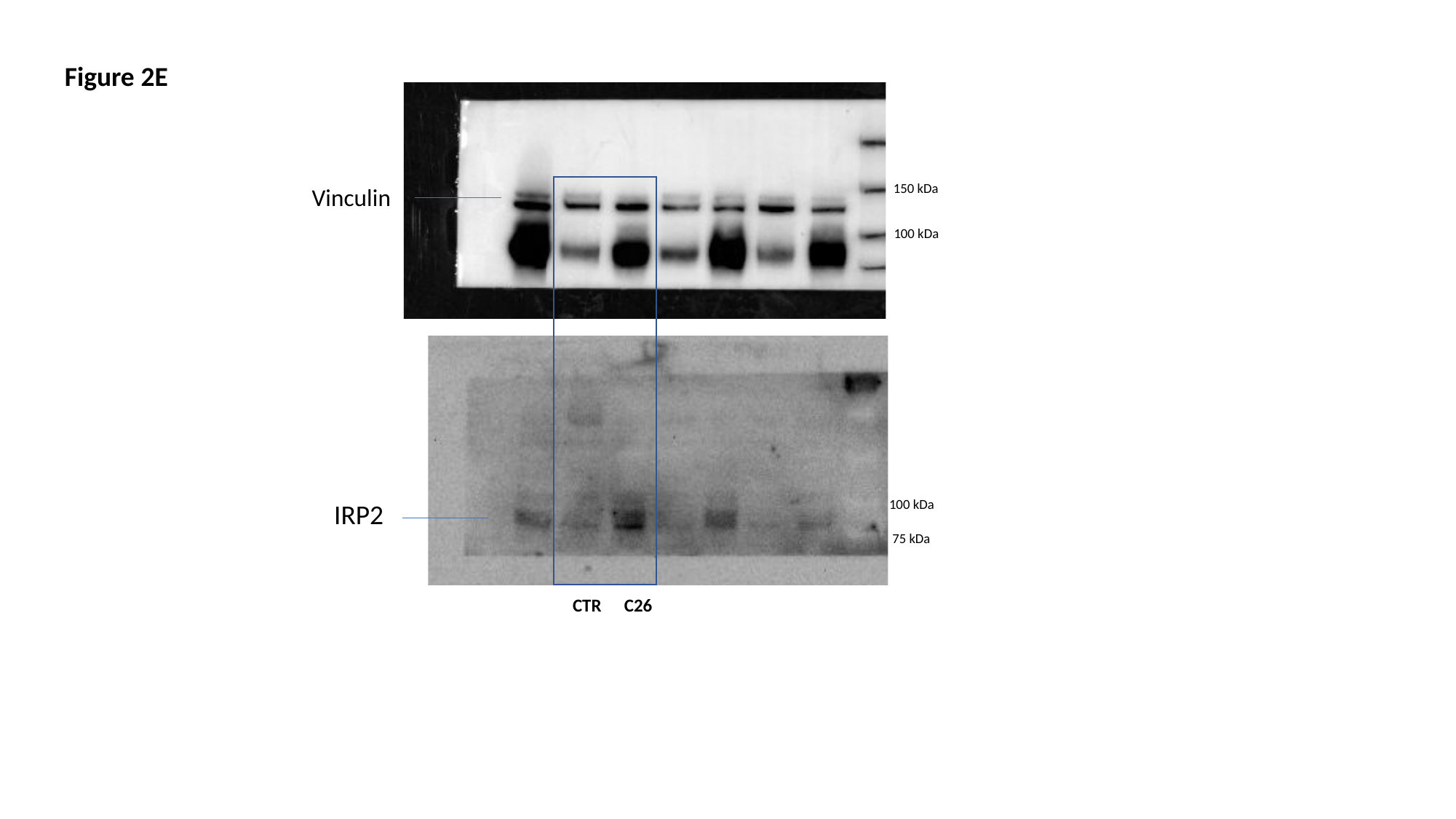

Figure 2E
150 kDa
Vinculin
100 kDa
100 kDa
IRP2
75 kDa
C26
CTR

## Slide 3
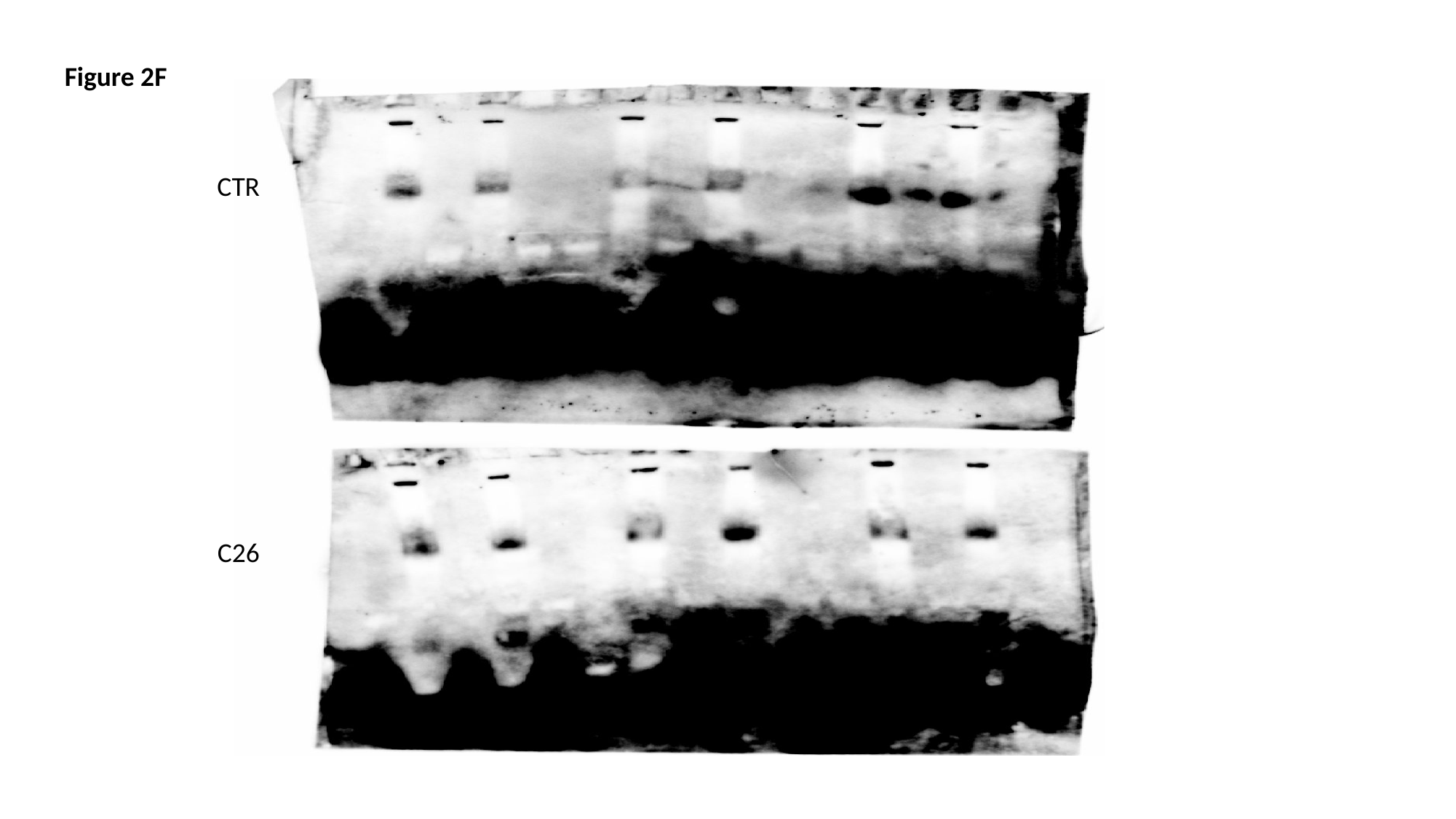

Figure 2F
CTR
C26

## Slide 4
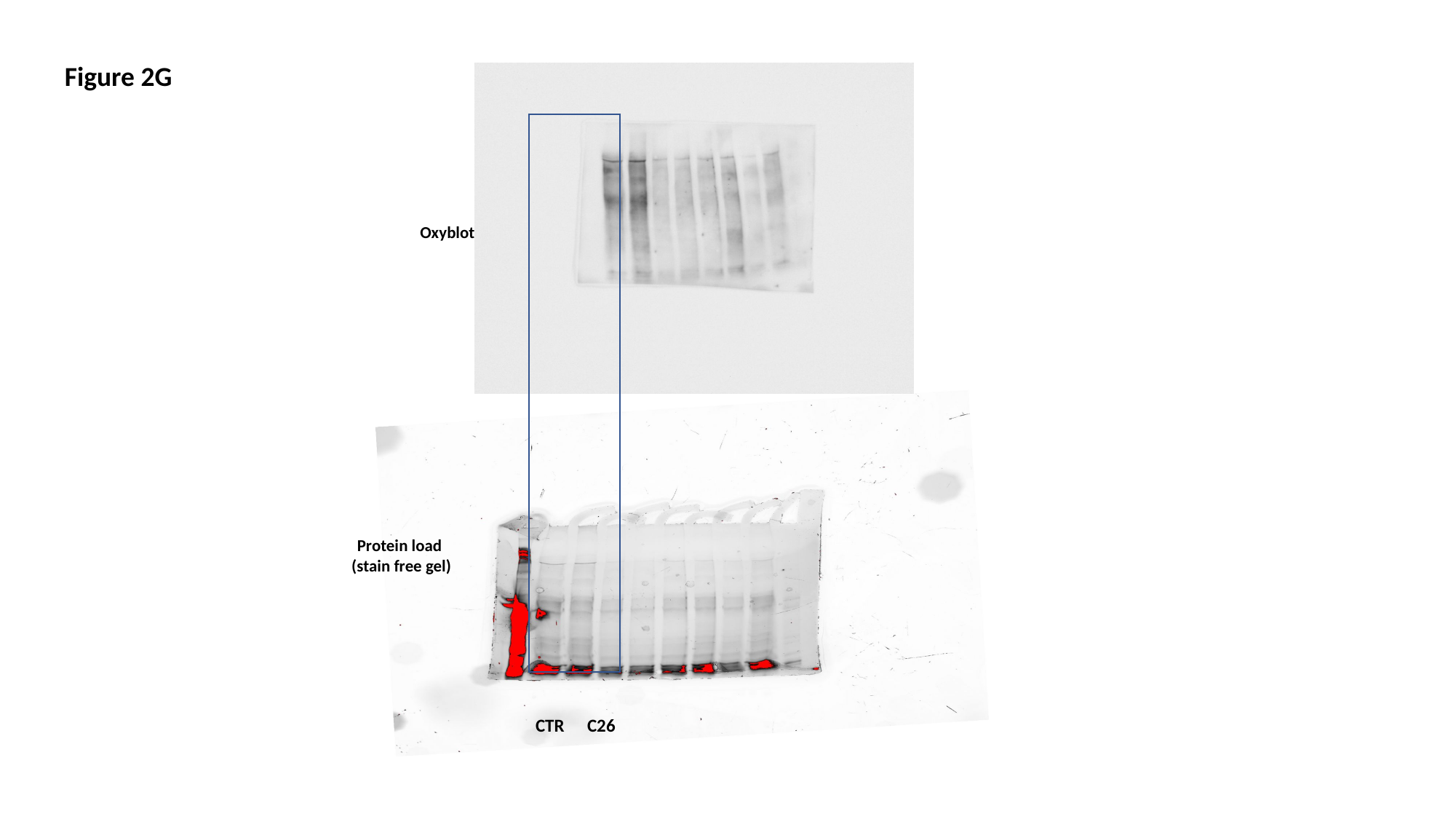

Figure 2G
Oxyblot
Protein load
(stain free gel)
C26
CTR

## Slide 5
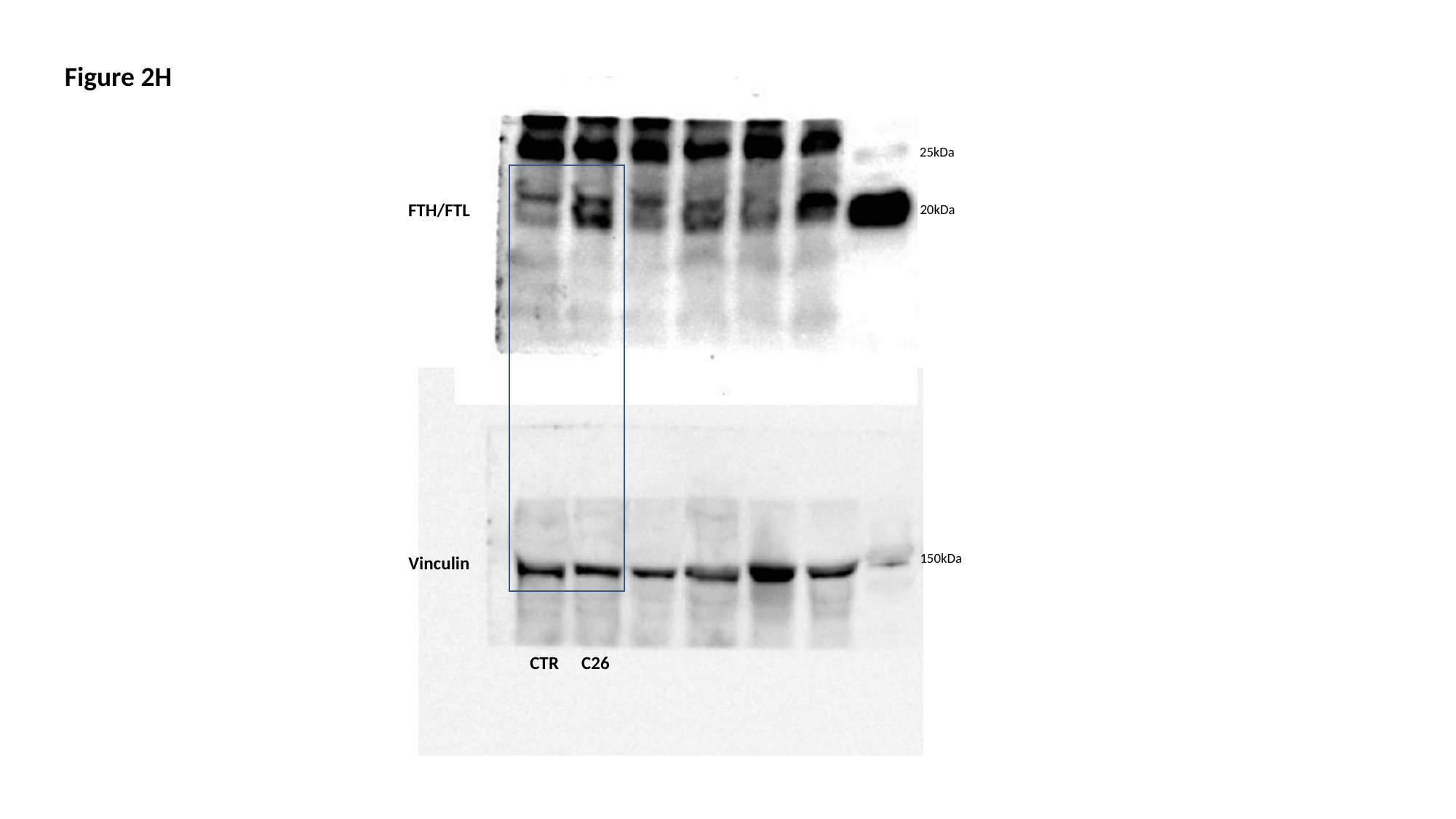

Figure 2H
25kDa
FTH/FTL
20kDa
150kDa
Vinculin
C26
CTR
