# Supplementary figures and images for: Iron supplementation is sufficient to rescue skeletal muscle mass and function in cancer cachexia
Source: EMBO Rep. 2022 Feb 24;23(4):e53746. doi: 10.15252/embr.202153746 (PMC8982578; doi:10.15252/embr.202153746)

## Slide 1
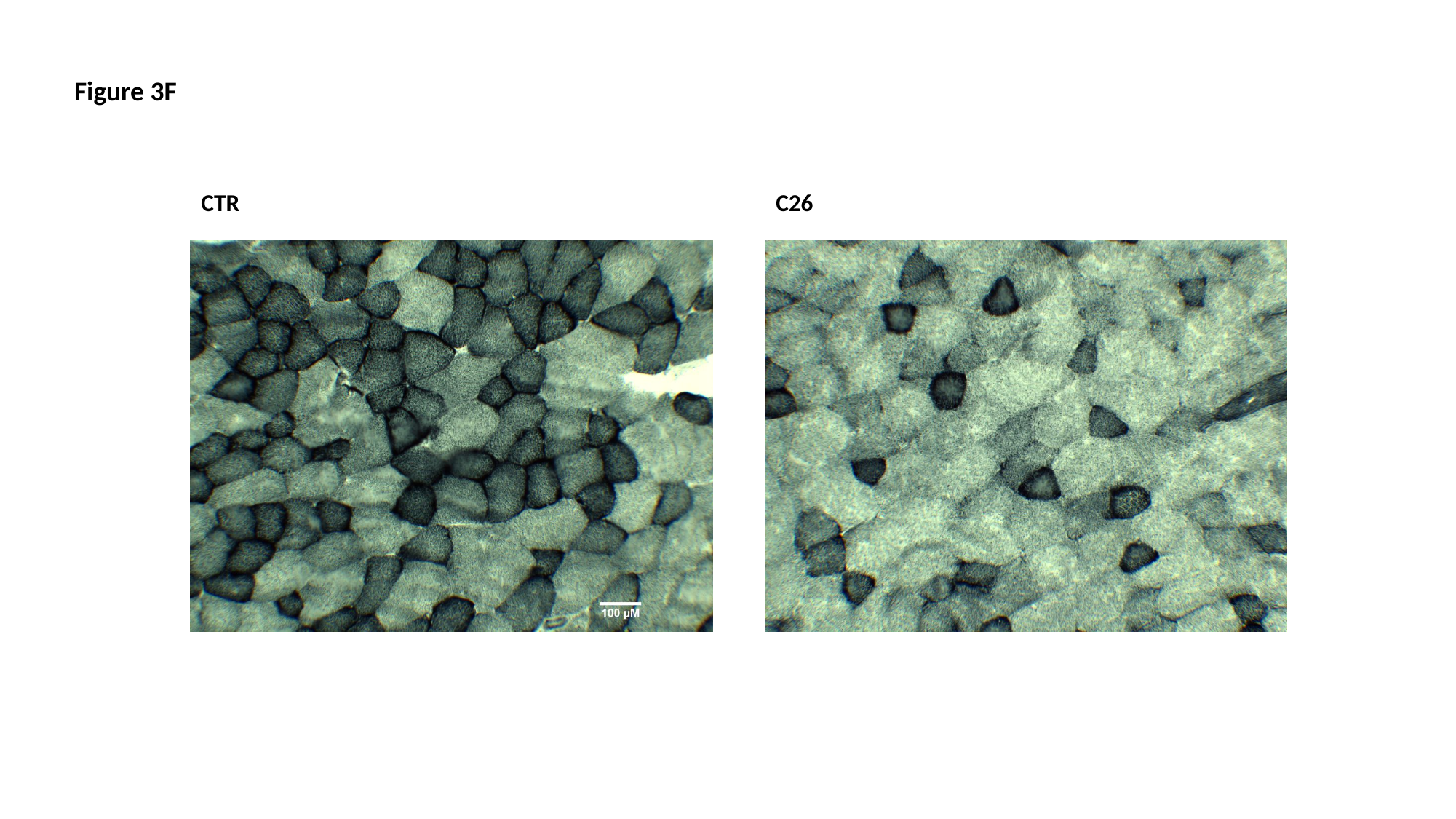

Figure 3F
CTR
C26

## Slide 2
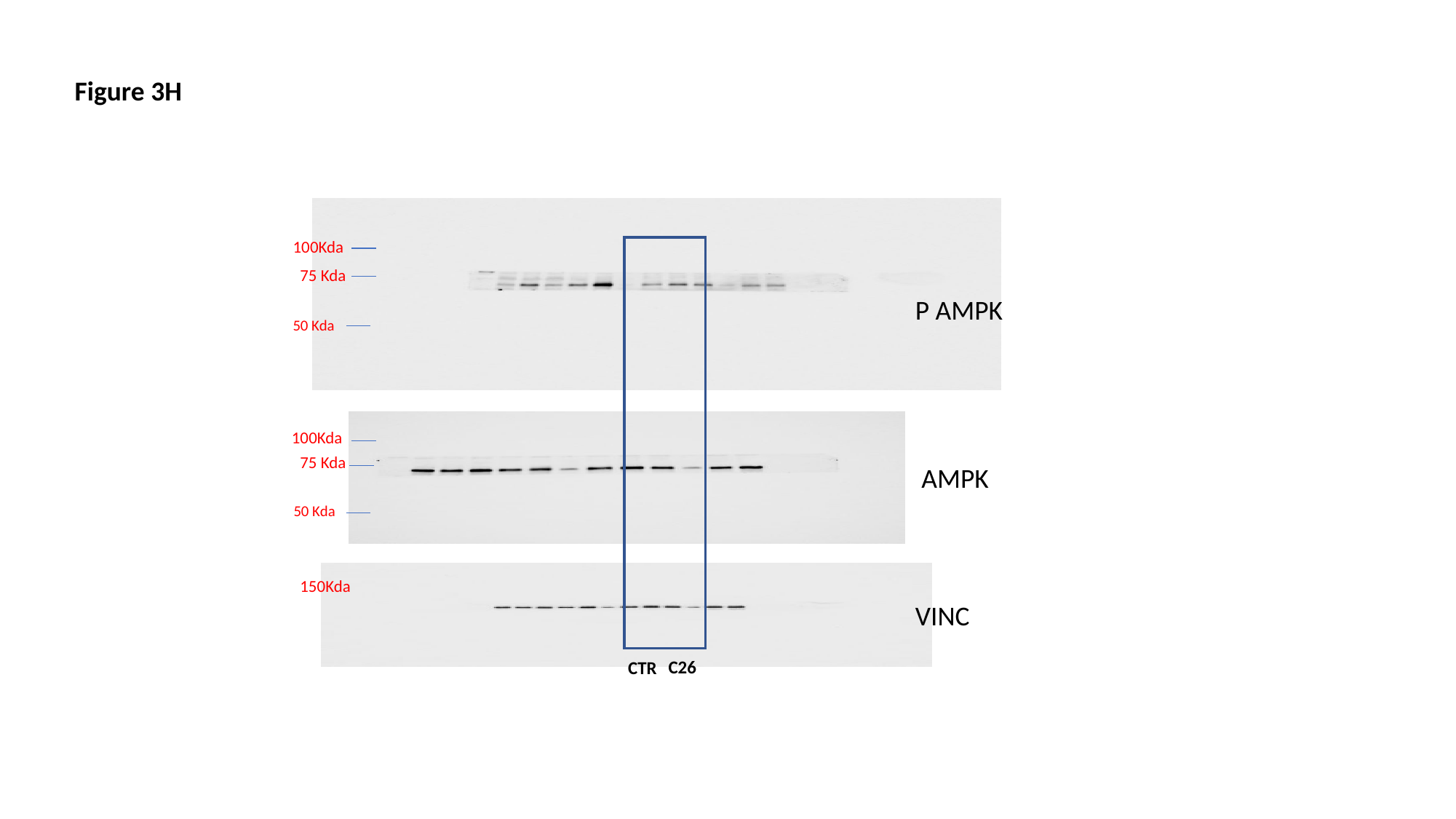

Figure 3H
100Kda
75 Kda
P AMPK
50 Kda
100Kda
75 Kda
 AMPK
50 Kda
150Kda
VINC
C26
CTR

Supplement: Supplementary file 6 — Source Data for Figure 3 [file EMBR-23-e53746-s007.pptx]

## Slide 1
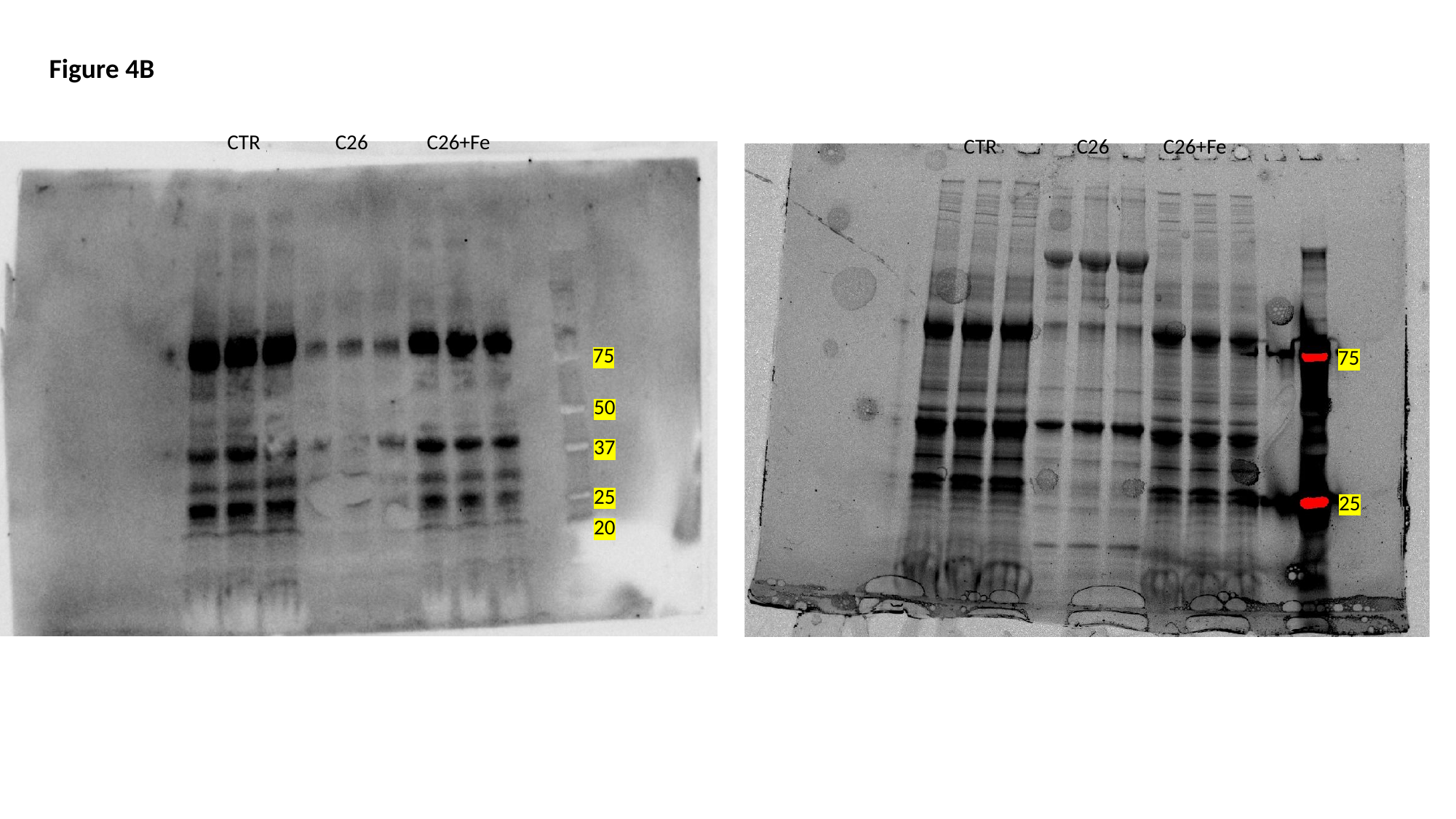

Figure 4B
CTR
C26
C26+Fe
CTR
C26
C26+Fe
75
75
50
37
25
25
20

## Slide 2
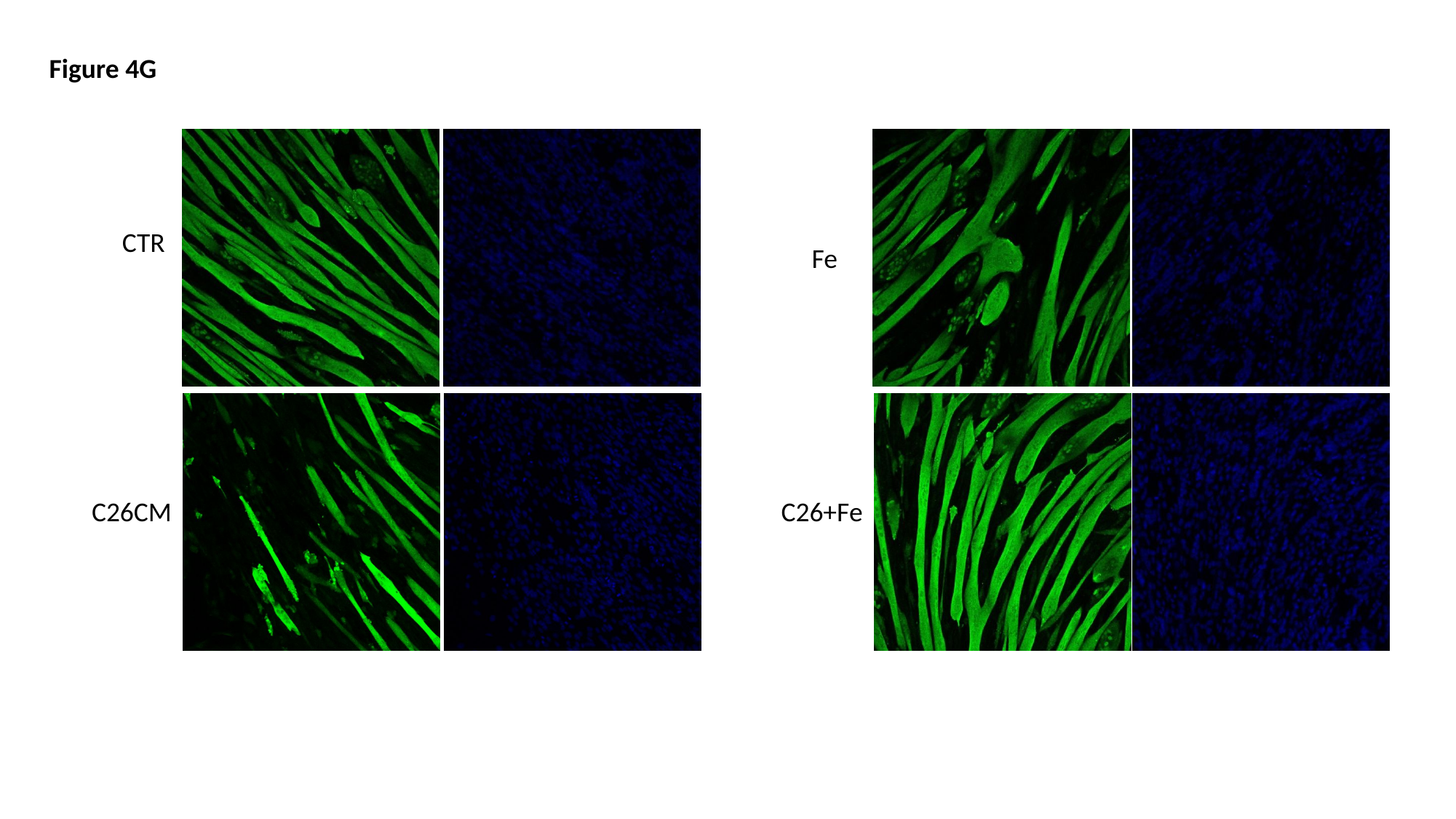

Figure 4G
CTR
Fe
C26CM
C26+Fe

## Slide 3
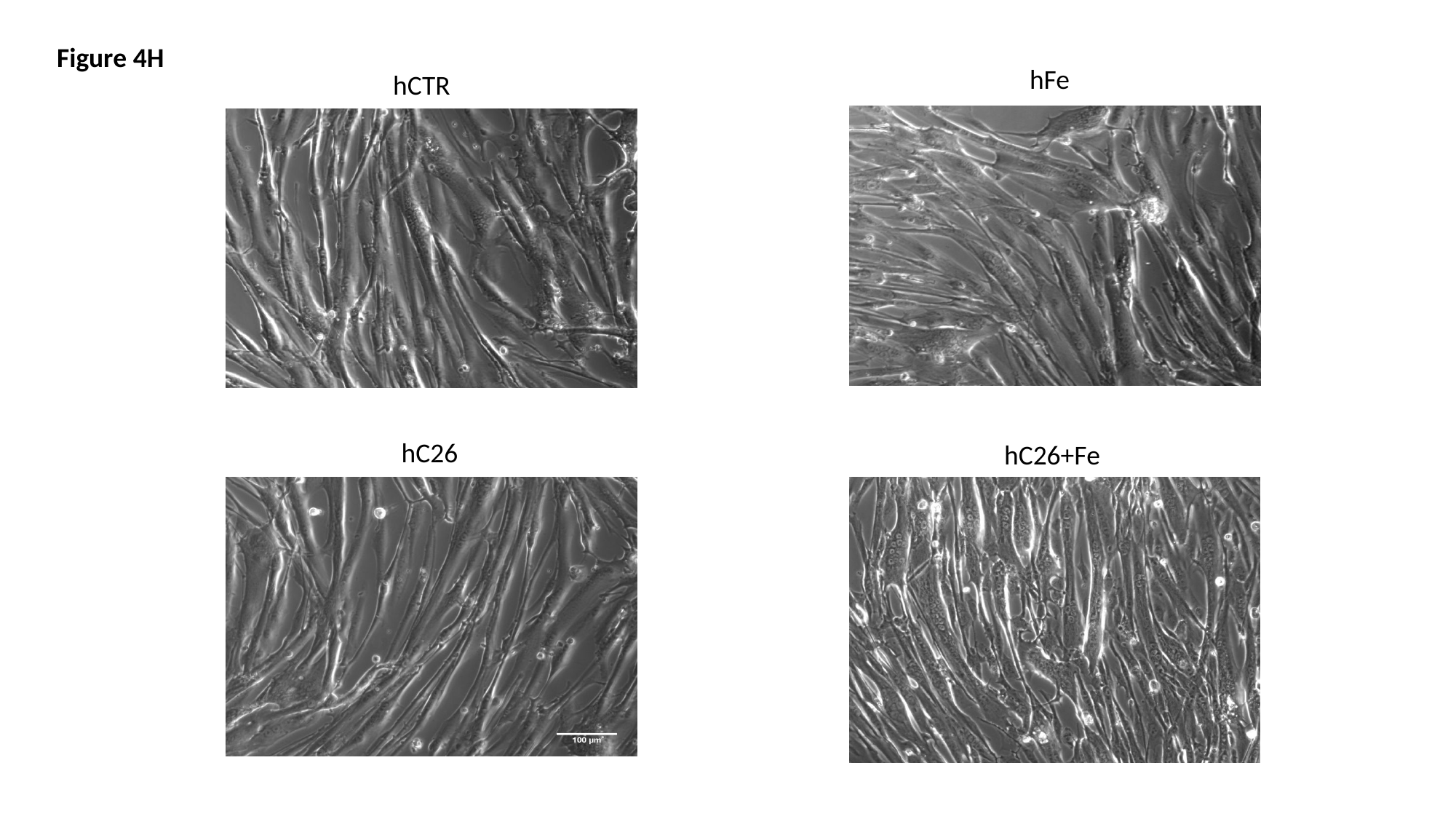

Figure 4H
hFe
hCTR
hC26
hC26+Fe

Supplement: Supplementary file 7 — Source Data for Figure 4 [file EMBR-23-e53746-s001.pptx]

## Slide 1
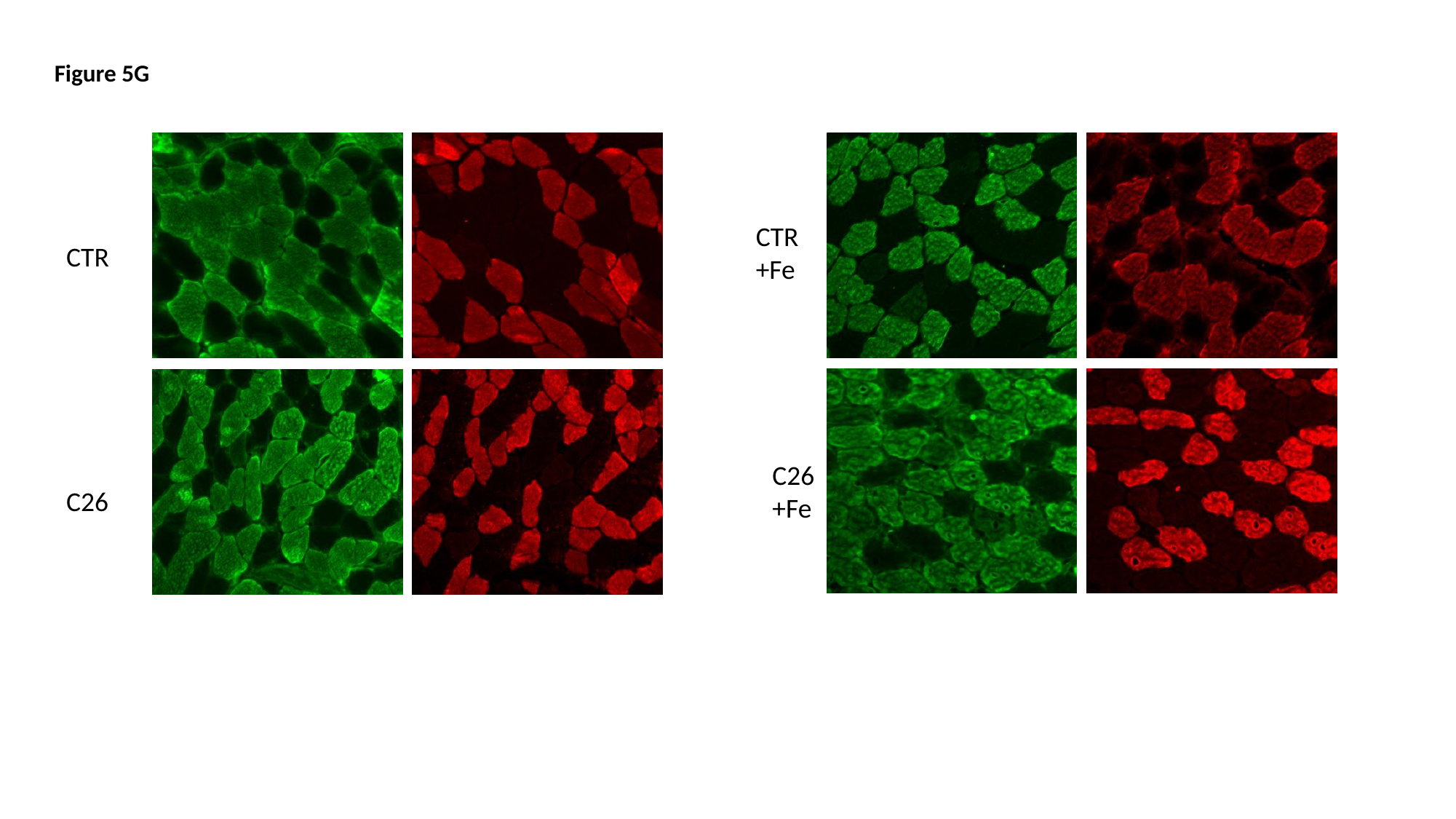

Figure 5G
CTR
+Fe
CTR
C26
+Fe
C26

Supplement: Supplementary file 8 — Source Data for Figure 5 [file EMBR-23-e53746-s004.pptx]

## Slide 1
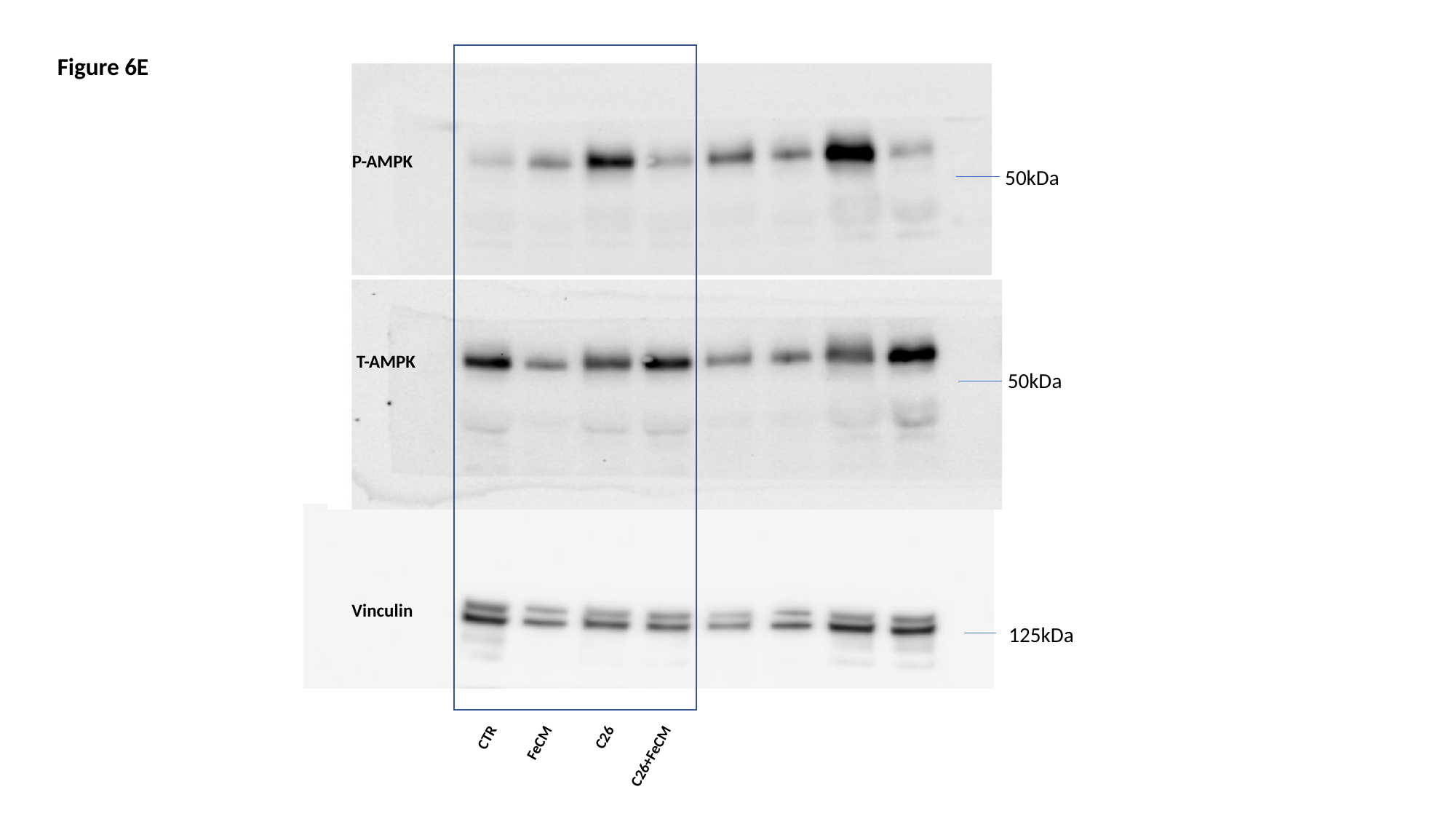

Figure 6E
P-AMPK
50kDa
T-AMPK
50kDa
Vinculin
125kDa
C26
CTR
FeCM
C26+FeCM

Supplement: Supplementary file 9 — Source Data for Figure 6 [file EMBR-23-e53746-s006.pptx]
