# Supplementary material for: Iron supplementation is sufficient to rescue skeletal muscle mass and function in cancer cachexia
Source: EMBO Rep. 2022 Feb 24;23(4):e53746. doi: 10.15252/embr.202153746 (PMC8982578; doi:10.15252/embr.202153746)
Supplement: Supplementary file 10 [file EMBR-23-e53746-s005.pdf]

EMBO reports

# Iron supplementation is sufficient to rescue cancer-induced muscle wasting and function.

Elisabeth Wyart, Myriam Hsu, Roberta Sartori, Erica Mina, Valentina Rausch, Elisa Pierobon, Mariarosa Mezzanotte, Camilla Pezzini, Laure Bindels, Andrea Lauria, Fabio Penna, Emilio Hirsch, Miriam Martini, Massimiliano Mazzone, Antonella Roetto, Simonetta Geninatti-Crich, Hans Prenen, Marco Sandri, Alessio Menga, and Paolo Porporato
**DOI: 10.15252/embr.202153746**

*Corresponding author(s): Paolo Porporato (paolo.porporato@unito.it)*

**Review Timeline:**

| | |
|---|---|
| Submission Date: | 3rd Aug 21 |
| Editorial Decision: | 23rd Aug 21 |
| Revision Received: | 7th Dec 21 |
| Editorial Decision: | 13th Jan 22 |
| Revision Received: | 20th Jan 22 |
| Accepted: | 25th Jan 22 |

*Editor: Deniz Senyilmaz Tiebe*

## Transaction Report:

(Note: With the exception of the correction of typographical or spelling errors that could be a source of ambiguity, letters and reports are not edited. Depending on transfer agreements, referee reports obtained elsewhere may or may not be included in this compilation. Referee reports are anonymous unless the Referee chooses to sign their reports.)

## 1st Editorial Decision 23rd Aug 2021

Dear Dr. Porporato,

Thank you for the submission of your research manuscript to our journal, which was now seen by three referees, whose reports are copied below.

We concur with the referees that the proposed link between iron metabolism and muscle cachexia is in principle very interesting. However, referees raise significant concerns that need to be addressed to consider publication here.

I find the reports informed and constructive, and believe that addressing the concerns raised will significantly strengthen the manuscript. As the reports are below, and I think all points need to be addressed, I will not detail them here.

Should you be able to address all referee concerns satisfactorily, we would like to invite you to revise your manuscript with the understanding that the referee concerns (as in their reports) must be fully addressed and their suggestions taken on board. Please address all referee concerns in a complete point-by-point response. Acceptance of the manuscript will depend on a positive outcome of a second round of review. It is EMBO reports policy to allow a single round of revision only and acceptance or rejection of the manuscript will therefore depend on the completeness of your responses included in the next, final version of the manuscript. Please note that we require strong support from the referees to consider publication here.

We generally allow three months as standard revision time. As a matter of policy, competing manuscripts published during this period will not negatively impact on our assessment of the conceptual advance presented by your study. However, we request that you contact the editor as soon as possible upon publication of any related work, to discuss how to proceed. Should you foresee a problem in meeting this three-month deadline, please let us know in advance and we may be able to grant an extension.

*** Temporary update to EMBO Press scooping protection policy:
We are aware that many laboratories cannot function at full efficiency during the current COVID-19/SARS-CoV-2 pandemic and have therefore extended our 'scooping protection policy' to cover the period required for a full revision to address the experimental issues highlighted in the editorial decision letter. Please contact the scientific editor handling your manuscript to discuss a revision plan should you need additional time, and also if you see a paper with related content published elsewhere.***

IMPORTANT NOTE: we perform an initial quality control of all revised manuscripts before re-review. Your manuscript will FAIL this control and the handling will be DELAYED if the following APPLIES:
1. A data availability section providing access to data deposited in public databases is missing (where applicable).
2. Your manuscript contains statistics and error bars based on n=2. Please use scatter plots in these cases.

You can submit the revision either as a Scientific Report or as a Research Article. For Scientific Reports, the revised manuscript can contain up to 5 main figures and 5 Expanded View figures. If the revision leads to a manuscript with more than 5 main figures it will be published as a Research Article. In this case the Results and Discussion section should be separate. If a Scientific Report is submitted, these sections have to be combined. This will help to shorten the manuscript text by eliminating some redundancy that is inevitable when discussing the same experiments twice. In either case, all materials and methods should be included in the main manuscript file

Supplementary/additional data: The Expanded View format, which will be displayed in the main HTML of the paper in a collapsible format, has replaced the Supplementary information. You can submit up to 5 images as Expanded View. Please follow the nomenclature Figure EV1, Figure EV2 etc. The figure legend for these should be included in the main manuscript document file in a section called Expanded View Figure Legends after the main Figure Legends section. Additional Supplementary material should be supplied as a single pdf labeled Appendix. The Appendix includes a table of content on the first page with page numbers, all figures and their legends. Please follow the nomenclature Appendix Figure Sx throughout the text and also label the figures according to this nomenclature. For more details please refer to our guide to authors.

Please note that for all articles published beginning 1 July 2020, the EMBO Reports reference style will change to the Harvard style for all article types. Details and examples are provided at
https://www.embopress.org/page/journal/14693178/authorguide#referencesformat

When submitting your revised manuscript, please carefully review the instructions that follow below. Failure to include requested items will delay the evaluation of your revision.

1) a .docx formatted version of the manuscript text (including legends for main figures, EV figures and tables). Please make sure that the changes are highlighted to be clearly visible.

2) individual production quality figure files as .eps, .tif, .jpg (one file per figure).

3) a .docx formatted letter INCLUDING the reviewers' reports and your detailed point-by-point responses to their comments. As part of the EMBO Press transparent editorial process, the point-by-point response is part of the Review Process File (RPF), which will be published alongside your paper. For more details on our Transparent Editorial Process, please visit our website: https://www.embopress.org/page/journal/14693178/authorguide#transparentprocess
You are able to opt out of this by letting the editorial office know (emboreports@embo.org). If you do opt out, the Review Process File link will point to the following statement: "No Review Process File is available with this article, as the authors have chosen not to make the review process public in this case."

4) a complete author checklist, which you can download from our author guidelines (<http://embor.embopress.org/authorguide>). Please insert information in the checklist that is also reflected in the manuscript. The completed author checklist will also be part of the RPF.

5) Please note that all corresponding authors are required to supply an ORCID ID for their name upon submission of a revised manuscript (<https://orcid.org/>). Please find instructions on how to link your ORCID ID to your account in our manuscript tracking system in our Author guidelines (<http://embor.embopress.org/authorguide>).

6) We replaced Supplementary Information with Expanded View (EV) Figures and Tables that are collapsible/expandable online. A maximum of 5 EV Figures can be typeset. EV Figures should be cited as 'Figure EV1, Figure EV2" etc... in the text and their respective legends should be included in the main text after the legends of regular figures.

- For the figures that you do NOT wish to display as Expanded View figures, they should be bundled together with their legends in a single PDF file called *Appendix*, which should start with a short Table of Content. Appendix figures should be referred to in the main text as: "Appendix Figure S1, Appendix Figure S2" etc. See detailed instructions regarding expanded view here: <http://embor.embopress.org/authorguide#expandedview>.

- Additional Tables/Datasets should be labeled and referred to as Table EV1, Dataset EV1, etc. Legends have to be provided in a separate tab in case of .xls files. Alternatively, the legend can be supplied as a separate text file (README) and zipped together with the Table/Dataset file.

7) We would also encourage you to include the source data for figure panels that show essential data.

Numerical data should be provided as individual .xls or .csv files (including a tab describing the data). For blots or microscopy, uncropped images should be submitted (using a zip archive if multiple images need to be supplied for one panel). Additional information on source data and instruction on how to label the files are available <http://embor.embopress.org/authorguide#sourcedata>.

8) Our journal encourages inclusion of *data citations in the reference list* to directly cite datasets that were re-used and obtained from public databases. Data citations in the article text are distinct from normal bibliographical citations and should directly link to the database records from which the data can be accessed. In the main text, data citations are formatted as follows: "Data ref: Smith et al, 2001" or "Data ref: NCBI Sequence Read Archive PRJNA342805, 2017". In the Reference list, data citations must be labeled with "[DATASET]". A data reference must provide the database name, accession number/identifiers and a resolvable link to the landing page from which the data can be accessed at the end of the reference. Further instructions are available at <http://embor.embopress.org/authorguide#datacitation>.

9) Please make sure to include a Data Availability Section before submitting your revision - if it is not applicable, make a statement that no data were deposited in a public database. Primary datasets (and computer code, where appropriate) produced in this study need to be deposited in an appropriate public database (see <http://embor.embopress.org/authorguide#dataavailability>).

Please remember to provide a reviewer password if the datasets are not yet public.

The accession numbers and database should be listed in a formal "Data Availability " section (placed after Materials & Method) that follows the model below. Please note that the Data Availability Section is restricted to new primary data that are part of this study.

# Data availability

The datasets (and computer code) produced in this study are available in the following databases:

- RNA-Seq data: Gene Expression Omnibus GSE46843 (https://www.ncbi.nlm.nih.gov/geo/query/acc.cgi?acc=GSE46843)
- [data type]: [name of the resource] [accession number/identifier/doi] ([URL or identifiers.org/DATABASE:ACCESSION])

*** Note - All links should resolve to a page where the data can be accessed. ***

10) Regarding data quantification, please ensure to specify the name of the statistical test used to generate error bars and P values, the number (n) of independent experiments underlying each data point (not replicate measures of one sample), and the test used to calculate p-values in each figure legend. Discussion of statistical methodology can be reported in the materials and methods section, but figure legends should contain a basic description of n, P and the test applied.
Please note that error bars and statistical comparisons may only be applied to data obtained from at least three independent biological replicates.
Please also include scale bars in all microscopy images.

We would also welcome the submission of cover suggestions, or motifs to be used by our Graphics Illustrator in designing a cover.

I look forward to seeing a revised version of your manuscript when it is ready. Please let me know if you have questions or comments regarding the revision.

Kind regards,

Deniz Senyilmaz Tiebe

Deniz Senyilmaz Tiebe, PhD
Editor
EMBO Reports

Referee #1:

This work provides links between iron metabolism and muscle cachexia. The authors conclude that muscle cachexia is associated with mitochondrial dysfunction due to mitochondrial iron deficiency. They show that iron supplementation prevents muscle wasting and improves muscle function in mouse models of cancer, and in a cohort of cancer patients. The data are potentially interesting, and highlight an important role of iron in muscle biology. Moreover, they have a translational potential. However, the mechanism leading to mitochondrial iron deficiency in the cancer models is not well defined.

1) A key initial observation is the reduced muscle TfR1 expression in patients and cancer models (Figs 1A, 1F, 1C), which appears to be critical for the size of muscle fibers. However, this is not associated with lower cellular iron content. Moreover, C2C12 cells treated with C26 conditioned media that develop a defective mitochondrial phenotype (Fig. 4A-E), seem to have increased TfR1 (Tfrc) expression (Fig. 4B and Table S1). Thus, the role of TfR1 in the development of muscle cachexia is questionable.
2) Did the iron depletion experiment (Fig. 1D-F) cause anemia in mice? Which was the iron content in the liver and muscles? Serum iron parameters?
3) Fig. 1J: Which are the effects of the treatments on TfR1 expression? How does apo-Tf operate as "TfR1 competitor", considering that it has ~500 times lower affinity than differic Tf?
4) The effects of cachexia on cellular iron metabolism are confusing. The authors show increased IRP2 expression but lower IRP2 activity. However, there is no proof that the IRE/IRP band in the EMSA represents IRP2. The authors seem to ignore IRP1, which is highly abundant in most mouse tissues. It is important to show how the IRP1 activity is affected under these experimental conditions. It should also be noted that IRP1/IRE and IRP2/IRE bands are easily separated in EMSAs with murine cell or tissue extracts; this is not the case in Figs 2E and Suppl. 2L).
5) Fig. 2G shows mRNA levels of ferroportin. This is not very informative; the authors should analyze protein expression.
6) In Fig. 3E, the data seem to correspond to total aconitase activity. The authors should assess mitochondrial and cytosolic (IRP1) aconitase activities separately.
7) In Fig. 3D, the authors state that the increased ALAS2 mRNA expression "reflects impaired IRP activity". This doesn't make any sense because IRPs do not control ALAS2 mRNA levels, but rather ALAS2 mRNA translation.
8) Fig. 4C-G shows that iron supplementation rescues mitochondrial respiration and myotube atrophy. Does it also reverse the gene expression phenotype depicted in Fig. A-B?
9) Fig. 5E shows that FeCM supplementation does not affect tumor weight. This appears counterintuitive because cancer cells have high requirements for iron. It will be important to examine whether the FeCM treatment affects iron content and expression of iron metabolism proteins in the tumor.
10) There are some typos in labeling of Figures (2SC-D, 6D, 6E).

Referee #2:

The manuscript by Wyart and Hsu et al. describes a novel role for muscle iron metabolism in cancer cachexia. The authors demonstrate that genes involved in iron metabolism are differentially expressed in cachectic muscle of mice or patients with cancer, correlating with reduced energy status of the cells and reduced muscle function. Cachexia and more specifically muscle weakness can be improved by treatment with ferric carboxymaltose, a drug approved for the treatment of iron deficiency. The presented data are of interest to the scientific community and for potential future treatment options.
The presented data seem to be of great quality, methods are adequately described, and the claims are supported by the data. Overall I am supportive of the manuscript as it combines mouse, cell culture, and patient data in a very convincing manner, thereby describing a novel important player in cachexia. Three major points warrant additional work in a revision as outlined below.
Major:
1) The literature regarding iron metabolism and cachexia (also associated to other chronic diseases besides cancer), muscle function, and cardiac function needs to be addressed better. For instance the presented data should be discussed in light of recent papers on this topic: PMID 33196891 (demonstrating the opposite, i.e. iron overload in muscle wasting), PMID 3005118 (showing iron deficiency in heart failure), or PMID 30178922
2) Fig. 1: Does the knockdown of TFR1 in muscle also lead to reduced intracellular iron levels? Or reduced iron availability? This connection should be demonstrated in order to prove the direct connection.
3) Fig. 4 (RNAseq): The authors write: "revealed clearly altered expression pattern of genes involved in electron transport chain, iron homeostasis (Fig. 4A and 3B) [...]". It should be Fig. 4A and 4B. Also, intriguingly, most genes involved in iron homeostasis (and also respiration and electron transfer) are upregulated in the dataset, with very small fold change. How is this increase explained? It goes against the claim of reduced mitochondrial activity. Significance should be indicated.
Even more importantly, the RNAseq data does not support the claims in the paper. Supplemental table 1 shows all significantly regulated pathways as result of the RNAseq, and also the gene lists shown in Fig. 4B. However, the pathways shown in Fig. 4B are not amongst the significantly regulated pathways, making it highly unlikely they play an important role in the C2C12 phenotype upon C26 CM treatment.
It is possible that the observed changes in iron homeostasis do not translate to large alterations in gene expression but are mediated by post-transcriptional events. However this needs to be addressed in a revised version of the manuscript. Do published sequencing data (for instance on cachectic muscle) support the authors' claims?

Minor:
- It is unclear which title is correct, as two titles are given. I think the second title is more accurate as iron compartmentalization has not been addressed in the paper.
- Fig. 2: TFR and IRP levels follow the same pattern as loading controls. These WB need to be more convincing. Including quantification and statistical testing would help (as done for Fig. 2F).
- Fig 4D, E: It seems Fe increases basal and ATP-linked OCR (as has previously been shown, for instance in PMID 29484788) but does cachexia significantly impair OCR? The effect size seems to be very small and a test of significance needs to be provided.
- Fig. 5: it should be specified in the text when these effects (improved body weight, muscle area etc.) were measured - was it at the end of the experiment (i.e. end of survival curve) or at an earlier time point?
- Fig. 6D and E are referred to wrongly in the text and in the figure legend
- Methods section, description of handgrip strength: Some text seems to be missing in the end of the paragraph
- Figure legend of Fig 5, incorrect labelling for I-K ("(I-K) mRNA levels of Murf 1 (G), Atrogin 1 (H), and REDD1 (I)").
- Figure legend 6, incorrect description of statistical testing (or perhaps the wrong statistical test was used as student's t test cannot be used for multiple comparisons)
- Figure legend 7B should specify what the red dots stand for

Referee #3:

Wyart, Hsu, and co-authors present an investigation into the role of iron in cancer cachexia. The authors suggest that when taken together, their data indicate that in cancer cachexia, a lack of iron accumulation within mitochondria is a cause of muscle wasting. Furthermore, they suggest that iron supplementation may be an effective means to rescue muscle weakness in patients with cancer cachexia. Despite the fact that this manuscript explores a new area of cancer cachexia, there are issues that must be addressed prior to publication.

Major Concerns
There are a number of issues in the manuscript that raise technical concerns. For example, from the graphs presented, it appears that the variability of the number of samples in several sets of figure panels that appear to have resulted from the same animal experiment is quite high. For example, in Figures 1D, 1E, and 1F, the control samples appear to have an n of 8, 5, and 3, while the treated samples are 5, 6, and 4. Figure 6 and Supplemental Figure 2 are other examples. The authors should clarify how they selected samples for their experiments and if outliers were excluded, that information should be included in the methods.

Additionally, the breaks in the axes of a significant number of graphs make actual differences between groups difficult to

interpret - the authors should determine if these are absolutely necessary to their data presentation. Examples include Figures 1D, 1H, 1I, 1J, 1K, and 2A. Without a compelling reason, these graphs should be adjusted to reflect the actual differences between groups.

In all myotube experiments, because myotubes continue to mature and increase in size after 4 days of differentiation, it is unclear to this reviewer if any of the effects shown actually reflect atrophy of the myotubes. Without a time zero control for treatment, it is possible that treatment with conditioned media, siRNA, etc, did not simply prevent or allow continued differentiation and in fact actually induced or prevented atrophy. This is point is particularly important because the authors assert that myogenesis is likely not the cause for improvement following iron treatment - but without doing these controls, these claims are not supported.

In Figure 5E, it is unclear at what timepoint the tumor volumes were measured - are all of these mice that were euthanized at 12 days, or does the figure include mice from the survival study? If the latter is accurate, then the claims that tumor volume were not affected by FeCM are not accurate. To truly make this claim, the data represented in Figure 5E should only be from mice euthanized 12 days following tumor cell injection.

In Figure 6, it is unclear why no data from either FeCM or control mice were presented for panels A through E. These data would provide an important contextualization about changes that are occurring during C26 tumor-bearing , which cannot be determined without including these groups. As currently presented, we only have information about how FeCM treatment during tumor-bearing changed these variables.

In Supplementary Figure 4A, it is unclear why grip strength is normalized to a percentage of Day 10 values, although the manuscript text suggests that the data are from day 6. This is particularly confusing to this reviewer, as by my understanding, these mice would have had a FeCM injection 5 days after tumor cell implantation, and therefore the data as presented are the difference between either two doses or zero doses. An unnormalized presentation of the data would more clearly represent the results of supplementation.

It is unclear why the one non-cancer subject was left in the dataset for Figure 7, if goal is to make argument that cancer patients benefit from iron supplementation, particularly because Supplemental Table 2 indicates that this person is otherwise healthy and significantly younger than all other included subjects. Lastly, the TSAT calculation should be included in the table, as it was a criterion used to determine iron deficiency.

Appropriate caveats of the human trial should also be noted somewhere in the manuscript - particularly the lack of a control group. If subjects were told that the treatment was going to improve their muscle strength, this amount of change isn't shocking.

Minor Concerns
In a number of figure legends, what ## or ### represents does not appear. For example, Supplementary Figure 3.

Figures 3H and 6D do not represent all data points as dot plots like all other graphs and should be updated. Also Figure 6 legend does not match the figure.

A row of Supplemental Table 2 appears to be mislabeled - two rows are labeled as non-dominant hand initial strength (kg).

In this reviewer's opinion, the supplemental movie does not add value to the manuscript.

**Point-by-Point Response**

**Referee #1:**

**This work provides links between iron metabolism and muscle cachexia. The authors conclude that muscle cachexia is associated with mitochondrial dysfunction due to mitochondrial iron deficiency. They show that iron supplementation prevents muscle wasting and improves muscle function in mouse models of cancer, and in a cohort of cancer patients. The data are potentially interesting, and highlight an important role of iron in muscle biology. Moreover, they have a translational potential. However, the mechanism leading to mitochondrial iron deficiency in the cancer models is not well defined.**

**A:** We would like to thank the reviewer for his/her thoughtful comments and efforts towards improving the quality of our manuscript. Below is our response to the issues raised in the review, including additional experiments to clarify and strengthen the proposed molecular mechanism. By quantifying iron (by ICP-MS) on different cellular compartments we demonstrated that mitochondrial iron is limited by the increase in protein-bound iron (separated by tissue dialysis). In this revised version, we provide additional mechanistic insights explaining the iron deficiency in muscle (i.e. altered TFR1 turnover and dysregulation of the IRE-IRP system).
Notably, we agree that the tissue specific regulation of iron metabolism in muscle (i.e. downregulating iron during iron deficiency) is an important point and further studies are required to fully elucidate this mechanism.

**1) A key initial observation is the reduced muscle TfR1 expression in patients and cancer models (Figs 1A, 1F, 1C), which appears to be critical for the size of muscle fibers. However, this is not associated with lower cellular iron content.**

**A:** As pointed out by the referee, we do not see any significant change in the total iron content of the skeletal muscle of C26-tumor bearing mice. However, a reduction of labile iron, which is characterized by doubled protein-bound iron (hence non bioavailable) and a lower mitochondrial iron was observed (**FIG 2J and FIG 3A**). Unfortunately, we cannot provide information about the muscle iron content in patients, but they presented significant anemia, as previously observed in PDAC patients (PMID 23567147).

**Moreover, C2C12 cells treated with C26 conditioned media that develop a defective mitochondrial phenotype (Fig. 4A-E), seem to have increased TfR1 (Tfrc) expression (Fig. 4B and Table S1). Thus, the role of TfR1 in the development of muscle cachexia is questionable.**

**A:** To directly assess the role of TFR1 in muscle mass regulation, we developed shRNA for *in vivo* silencing of TFR1 by electroporation (**FIG1G**). While we previously found that the overexpression of TFR1 in muscle (**FIG1H)** promoted fiber hypertrophy, on the contrary TFR1 knock-down by shRNA resulted in muscle fiber atrophy in healthy mice (**R1-Fig1a and updated FIG1G**). These data establish the direct role of TFR1 expression in the control of muscle mass, independently from other factors ( e.g. inflammation, cancer, starvation.).

Consistently, *in vitro*, TFR1 silencing promoted myotube atrophy which was rescued by an iron ionophore (hinokitiol, **FIG EV3D**) supporting the role of TFR1 in regulating atrophy *via* iron import. Importantly, as pointed-out by the reviewer TFR1 is transcriptionally upregulated *in vitro*. However, this appears to be an unsuccessful compensatory mechanism as total protein levels are unchanged (**R1-Fig1b**). This observation could result from the different kinetics occurring in the two different atrophic processes observed *in vitro* and *in vivo* (48h vs 12 days).
Nevertheless, we performed additional experiments showing that TFR1 function is deficient also *in vitro* as C26-CM treated myotubes are unable to import transferrin (*i.e.* transferrin internalization assay) (**R1-Fig1c**), which was further confirmed by a decrease in intracellular iron bioavailability assessed by calcein-AM assay (**R1-Fig1d**). We believe these data on altered iron internalization fall beyond the scope of this paper but are worth to be further studied in future works.

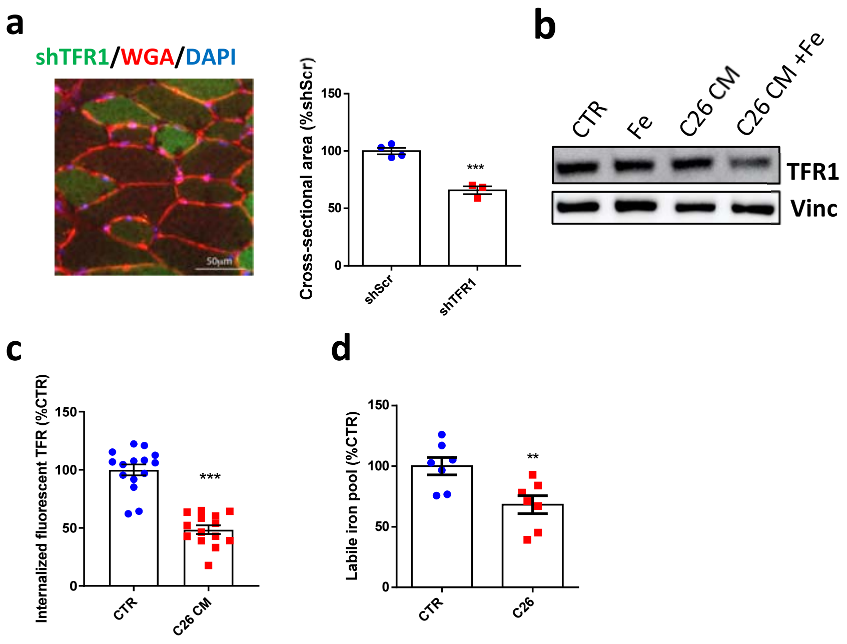


**R1-Fig.1: (a)** Measurement of cross-sectional area (CSA) of GFP positive myofibers (expressing either shScramble or shTFR1) normalized to the one of GFP negative fibers in the tibialis anterior of mice two weeks after *in vivo* electroporation. Right panel shows a representative magnified picture of a transversely cut tibialis anterior electroporated with shTFR-pGFP. **(b)** TFR1 levels in C2C12 myotubes treated for 48h with C26 conditioned medium. **(c)** Transferrin-mediated iron internalization quantified by fluorescence. C2C12 myotubes were treated with C26CM for 48h and were then incubated with fluorochrome-conjugated Tf for 1h. Fluorescence levels were measured with a plate reader and values were normalized to cell number. **(d)** Labile iron pool measured by calcein-AM assay.

**2) Did the iron depletion experiment (Fig. 1D-F) cause anemia in mice? Which was the iron content in the liver and muscles? Serum iron parameters?**

**A:** We included blood parameters and found that the combination of phlebotomy and iron deficient diet led to significant anemia and increased circulating transferrin in mice (**R1-Fig2a-b and updated FIG EV1A**), confirming the induction of iron deficiency. Notably, the iron content of liver was significantly decreased (**R1-Fig2c**).

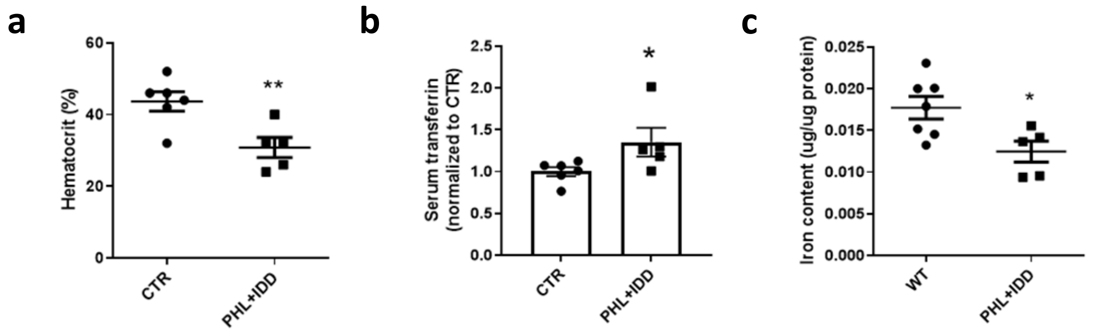


**R1-Fig. 2: (a)** Hematocrit of mice fed with normal diet (CTR) or receiving phlebotomy (PHL) plus iron deficiency diet (IDD). **(b)** Serum transferrin levels assessed by immunoblotting. **(c)** Liver iron content measured by ICP-MS.

3) **Fig. 1J: Which are the effects of the treatments on TfR1 expression? How does apo-Tf operate as "TfR1 competitor", considering that it has ~500 times lower affinity than differic Tf?**

**A:** In our experimental context we found a physiological upregulation of TFR1 with deferoxamine (DFO) but not with other treatments (**R1-Fig3a**). In addition, we further checked intracellular iron bioavailability using Calcein-AM assay (PMID11969183). Coherently, all iron chelators and apo-Tf promoted a decrease in labile iron pool (**R1-Fig3b FIG EV1H**).

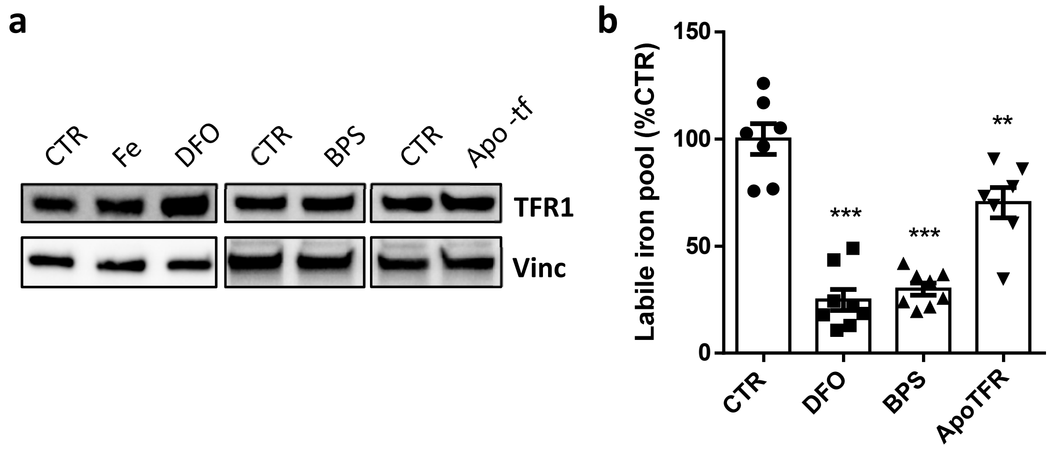


**R1- Fig. 3:** TFR1 protein levels in C2C12 treated for 48h with **(a)** iron or iron chelators DFO, BPS or apo-Tf. **(b)** Labile iron pool quantified by calcein-AM assay.

Concerning apoTF**,** we rephrased the related sentence for clarity as "TFR1 competitor" is not the expected mechanism of apo-TF. Indeed, it has been shown *in vitro* that apo-TF treatment reduces Holo-TF amount (while increasing the formation of mono-TF), leading to overall decreased iron uptake (PMID 29248829) and https://ashpublications.org/blood/article/124/21/4037/115083/Exogenous-Apo-Transferrin-Increases-Monoferric, as confirmed by our data.

**4) The effects of cachexia on cellular iron metabolism are confusing. The authors show increased IRP2 expression but lower IRP2 activity. However, there is no proof that the IRE/IRP band in the EMSA represents IRP2. The authors seem to ignore IRP1, which is highly abundant in most mouse tissues. It is important to show how the IRP1 activity is affected under these experimental conditions. It should also be noted that IRP1/IRE and IRP2/IRE bands are easily separated in EMSAs with murine cell or tissue extracts; this is not the case in Figs 2E and Suppl. 2L).**

**A:** We thank the reviewer for pointing out the role of IRP1 and we apologize for the lack of clarity, we improved the description of this part. Initially, we focused on IRP2 since it has been previously reported that the very small RNA-binding fraction of IRP1 is insensitive to cellular iron status in animal tissues, whereas IRP2 controls post-transcriptional regulation of iron metabolism (PMID: **14726953).** To assess the role of IRP1 in our model, we measured its enzymatic activity by aconitase assay following fractionation, and we observed a decreased activity in the cytosolic fraction (**R1-Fig4a and updated FIG2D**), indicating the switch of the enzyme to IRE binding function (PMID: 16850017).
Moreover, for the REMSA we better resolved the RNA-protein complexes on 4% non-denaturing polyacrylamide gels instead of 6%, since in (**FIG EV2L**) a double band was observed. Surprisingly, this technical improvement allowed us to unravel the impairment of IRP1 RNA binding activity in C26 muscles not only in native but also in reducing conditions (**R1-Fig4b**).

To further confirm these findings, we tried to perform a supershift assay with anti-IRP1 and IRP2 antibodies. Unfortunately, REMSA assay on skeletal muscles was very challenging and we were not able to resolve the RNA-IRP-antibody complexes without having smeared signals (**R1-Fig4c**).

In conclusion, we observed an involvement of IRP1 in C26 muscles. Hence, we modified different parts of the manuscript to include also the data showing the impairment of IRP1/aconitase and RNA binding activities, with the last one only partially rescued by reducing conditions, suggesting the presence of other mechanisms of down-modulation.

**a**

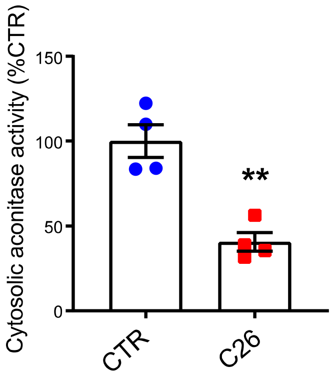


**b**

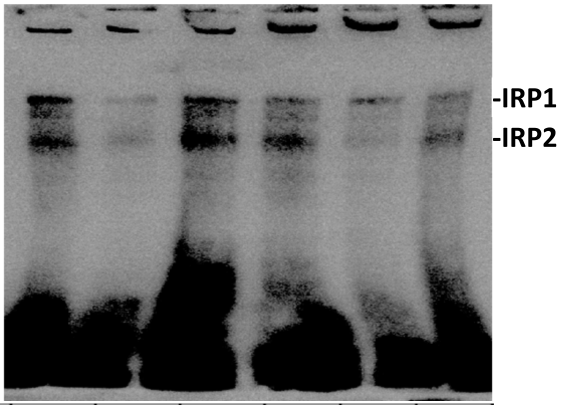


| Condition | 1 | 2 | 3 | 4 | 5 | 6 |
|---|---|---|---|---|---|---|
| DTT/EDTA | + | + | - | + | + | - |
| Biotin-IRE RNA | + | + | + | + | + | + |
| 200-fold molar excess of unlabeled IRE RNA | - | + | - | - | + | - |
| CTR Cytosolic Muscle Extract | + | + | + | - | - | - |
| C26 Cytosolic Muscle Extract | - | - | - | + | + | + |

**c**

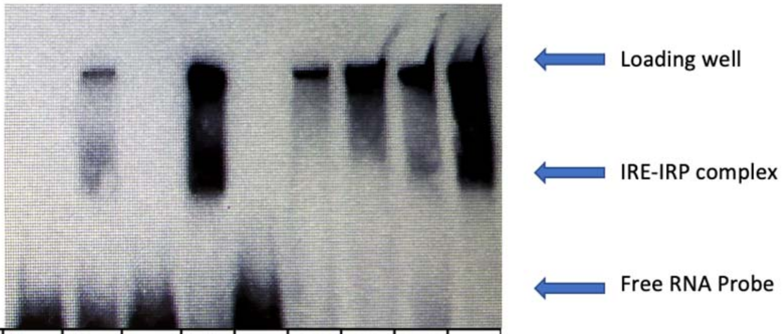


| Condition | 1 | 2 | 3 | 4 | 5 | 6 | 7 | 8 | 9 |
|---|---|---|---|---|---|---|---|---|---|
| DTT/EDTA | + | + | + | + | + | + | + | + | + |
| Biotin-IRE RNA | + | + | + | + | + | + | + | + | + |
| 200-fold molar excess of unlabeled IRE RNA | - | - | + | - | + | - | - | - | - |
| CTR Cytosolic Muscle Extract | - | + | + | - | - | + | - | + | - |
| C26 Cytosolic Muscle Extract | - | - | - | + | + | - | + | - | + |
| Anti-IRP1 | - | - | - | - | - | + | + | - | - |
| Anti-IRP2 | - | - | - | - | - | - | - | + | + |

**R1- Fig. 4: (a)** Cytosolic aconitase activity assessed after fractionation. **(b)** IRP REMSA in native and reducing conditions. **(c)** IRP supershift in reducing conditions assessed in cytosolic extract from gastrocnemius.

**5) Fig. 2G shows mRNA levels of ferroportin. This is not very informative; the authors should analyze protein expression.**

**A:** We thank the reviewer for his/her suggestion. We carefully examined ferroportin protein expression *in vivo* and, unlike TfR1, the upregulation of FPN in mRNA levels does not correlate with protein levels. As protein levels of FPN appears to be very low in muscle we think the antibodies used were not sufficiently sensitive. Hence, we prefer to remove the transcriptional data as they are not essential for the message of the paper.

**6) In Fig. 3E, the data seem to correspond to total aconitase activity. The authors should assess mitochondrial and cytosolic (IRP1) aconitase activities separately.**

**A**: We thank the reviewer for this constructive comment. We show in the text the total aconitase activity as aconitase (both mitochondrial and cytosolic) requires iron for their enzymatic activity. However only the cytosolic aconitase plays the role of iron regulating protein once it loses enzymatic activity. To address this, we replicated an *in vivo* experiment and performed fractionation prior to aconitase assay on freshly isolated samples. We were

able to appreciate that muscle presents a substantial portion of aconitase activity in the cytosol which is inactivated during cachexia in an iron-dependent manner (**R1-Fig5a and updated FIG2D**). The same trend could be observed in both mitochondrial and total lysates (**R1-Fig6b-c and updated FIG6B and EV4G).**

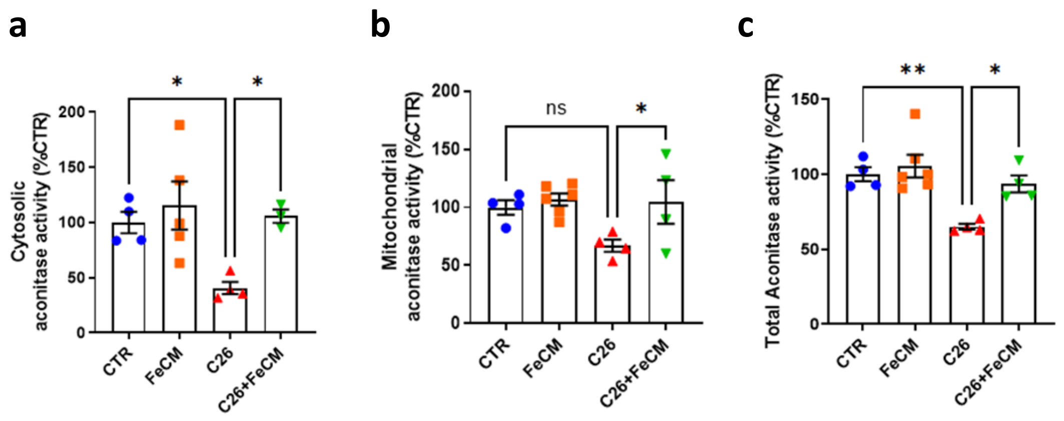


**R1-Fig.5:** Enzymatic activity of aconitase measured in **(a)** total lysate **(b)** cytosolic and **(c)** mitochondrial fraction of skeletal muscle in C26 bearing mice supplemented with iron.

**7) In Fig. 3D, the authors state that the increased ALAS2 mRNA expression "reflects impaired IRP activity". This doesn't make any sense because IRPs do not control ALAS2 mRNA levels, but rather ALAS2 mRNA translation.**

**A:** We thank the reviewer for the remark, we have now discussed the potential effects of ALAS2 overexpression on muscle atrophy and related mitochondrial dysfunction in light of the work of Peng et al. (PMID 33785075).

**8) Fig. 4C-G shows that iron supplementation rescues mitochondrial respiration and myotube atrophy. Does it also reverse the gene expression phenotype depicted in Fig. A-B?**

**A**: Since the RNAseq data did not match with the protein levels, (Reviewer 2 suggestion, point 1), we decided to remove this part of data and focus directly on the effects of iron supplementation on protein expression of mitochondrial complexes subunits (based on PMID 30059724). Interestingly, we observed a significant downregulation of the OXPHOS subunits in C26-treated myotubes that was reversed by iron supplementation **(R1-Fig6a and updated FIG4B).** Since mtDNA replication relies mostly on the cytoplasmic dNTP production catalyzed by the enzyme ribonucleotide reductase (an iron-dependent enzyme PMID 31793879), we evaluated the effects on mtDNA copy number and we found that iron treatment is able to prevent mtDNA instability (**R1-Fig6b and updated FIG4A)** in line with rescued expression of COXII-subunit of CIV (updated **FIG 4B**). We believe that these new data are providing a better mechanistic explanation of the observed bioenergetic dysfunction.

**9) Fig. 5E shows that FeCM supplementation does not affect tumor weight. This appears counterintuitive because cancer cells have high requirements for iron. It will be important to examine whether the FeCM treatment affects iron content and expression of iron metabolism proteins in the tumor.**

**A:** To answer this point, we performed an analysis of iron metabolism proteins in the tumor following iron treatment. Despite increased iron content, tumors from mice treated with FeCM presented a physiological response to iron loading (i.e. TFR1 downregulation and FT upregulation) to maintain iron homeostasis. These findings are congruent with the unchanged tumor growth **(R1-Fig7a, FIG 5E and updated FIG EV4F).**

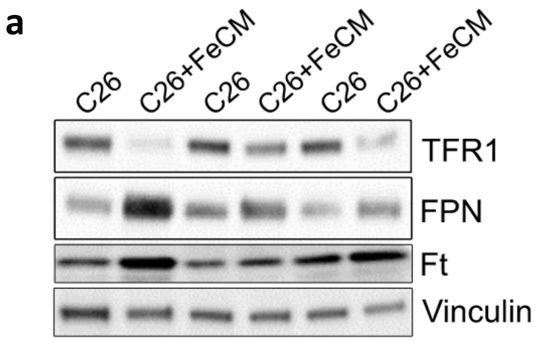


**R1-Fig.7: (a)** Protein expression of iron-trafficking proteins by C26-tumor extracted from mice following iron supplementation

**10) There are some typos in labeling of Figures (2SC-D, 6D, 6E).**

**A**: Thank you for the remark, the labeling is revised and corrected.

**Referee #2:**

**The manuscript by Wyart and Hsu et al. describes a novel role for muscle iron metabolism in cancer cachexia. The authors demonstrate that genes involved in iron metabolism are differentially expressed in cachectic muscle of mice or patients with cancer, correlating with reduced energy status of the cells and reduced muscle function. Cachexia and more specifically muscle weakness can be improved by treatment with ferric carboxymaltose, a drug approved for the treatment of iron deficiency. The presented data are of interest to the scientific community and for potential future treatment options.**
**The presented data seem to be of great quality, methods are adequately described, and the claims are supported by the data. Overall I am supportive of the manuscript as it combines mouse, cell culture, and patient data in a very convincing manner, thereby describing a novel important player in cachexia. Three major points warrant additional work in a revision as outlined below.**

We thank the referee for the careful and insightful review of our manuscript. As outlined below, we addressed all the concerns of the referee.

**Major:**

**1) The literature regarding iron metabolism and cachexia (also associated to other chronic diseases besides cancer), muscle function, and cardiac function needs to be addressed better. For instance the presented data should be discussed in light of recent papers on this topic: PMID 33196891 (demonstrating the opposite, i.e. iron overload in muscle wasting), PMID 3005118 (showing iron deficiency in heart failure), or PMID 30178922**

**A**: We now added more references in our discussion to better cover the state of the art. Indeed, data derived from cardiac patients are very interesting as well as the review proposed (PMID 30178922). We would like to point out that most of these papers associate iron to defect in erythropoiesis, while our work is linking iron to rapid modulation of strength, suggesting that these findings might have a potential impact also in the case of cardiac dysfunction. Importantly, the study by Zhou et al. (PMID 33196891) is now discussed, showing that iron levels are increased in the skeletal muscle of cachectic and sarcopenic gastric cancer patients compared to cancer patients without weight loss. Nonetheless, this paper does not include healthy controls in the presented cohort. Moreover, the “aging factor” has to be considered as a potential confounding factor, promoting muscle atrophy in a significant different way from cancer, as reported in our model. As discussed in our paper, the increase of iron in this model is minimal, while there is an important increase in ferritin, suggesting that the present iron is not bioavailable as observed in our model.

**2) Fig. 1: Does the knockdown of TFR1 in muscle also lead to reduced intracellular iron levels? Or reduced iron availability? This connection should be demonstrated in order to prove the direct connection.**

**A**: Thank you for raising this point. We measured in C2C12 myotubes the labile iron pool after silencing with esiTFR1 using calcein-based fluorescence assay (**R2-Fig1 a and FIG EV1F**), and we confirm that intracellular free iron (non-chelated or complexed) is significantly decreased by more than 20%. In addition, we added our validation experiment on 3T3 cells with TFR1-silencing plasmids used for *in vivo* electroporation (**updated FIG1G and EV1D**). Importantly, we detected a significant downregulation of ferritin following TFR1 silencing, which is physiological response to buffer the decreased intracellular iron.

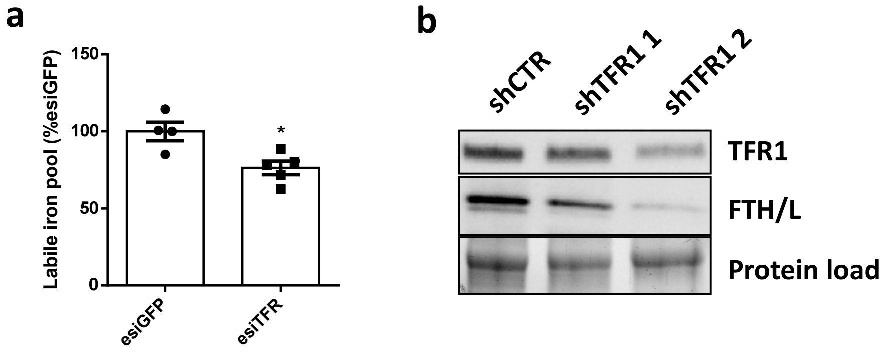


**R2-Fig.1: (a)** Labile iron pool quantified by calcein-AM assay. **(b)** Representative western blot showing transferrin receptor1 and ferritin expression in 3T3 cells following transfection of TFR1-silencing plasmids (48h post transfection).

**3) Fig. 4 (RNAseq): The authors write: "revealed clearly altered expression pattern of genes involved in electron transport chain, iron homeostasis (Fig. 4A and 3B) [...]". It should be Fig. 4A and 4B. Also, intriguingly, most genes involved in iron homeostasis (and also respiration and electron transfer) are upregulated in the dataset, with very small fold change. How is this increase explained? It goes against the claim of reduced mitochondrial activity. Significance should be indicated.**
**Even more importantly, the RNAseq data does not support the claims in the paper. Supplemental table 1 shows all significantly regulated pathways as result of the RNAseq, and also the gene lists shown in Fig. 4B. However, the pathways shown in Fig. 4B are not amongst the significantly regulated pathways, making it highly unlikely they play an important role in the C2C12 phenotype upon C26 CM treatment.**
**It is possible that the observed changes in iron homeostasis do not translate to large alterations in gene expression but are mediated by post-transcriptional events. However this needs to be addressed in a revised version of the manuscript. Do published sequencing data (for instance on cachectic muscle) support the authors' claims?**

We thank the reviewer for raising this critical point. Indeed, we performed a systemic analysis of protein expression of main targets of the RNAseq, which indicated that the transcriptional data do not match protein expression of altered targets. Moreover, the analysis of other meta-analyses of publicly available dataset revealed that our RNAseq data is not in line with genome-wide transcriptome data (PMID 30059724) performed *in vivo*. Therefore, we decided to remove these data from the manuscript and to include new experiments in order to evaluate mitochondrial fitness. We evaluated the protein expression of several mitochondrial complexes subunits and we observed a strong decrease in C2C12 myotubes (in line with the aforementioned metanalysis PMID 30059724) and surprisingly a rescue with iron (**updated Fig. 4B**). Since COXII (complex IV subunit) is encoded by mtDNA and iron dyshomeostasis is associated with mtDNA instability (PMID 31793879), we evaluated mtDNA copy number and we found a significant decrease in C2C12 myotubes-CM treated recovered by iron treatment (**updated FIG. 4A**), which is also in line with previously published data (PMID 31793879).

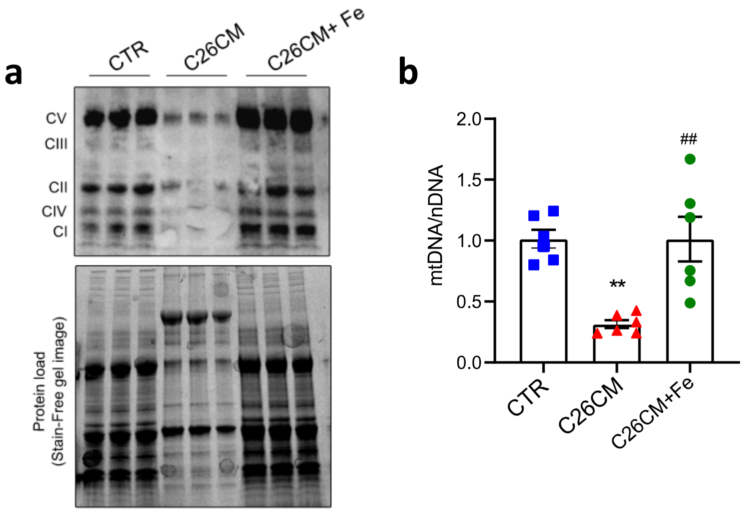


**R2-Fig.1: (a)** Representative western blot of mitochondrial OXPHOS respiratory complexes in C2C12 myotubes treated for 48h with C26CM and iron. **(b)** Mitochondrial DNA (mtDNA) on nuclear DNA (nDNA) quantified by qPCR in C2C12 myotubes treated for 48h with C26CM and iron

Minor:

**- It is unclear which title is correct, as two titles are given. I think the second title is more accurate as iron compartmentalization has not been addressed in the paper.**

**A**: We agree with the referee that the second title is more accurate. We have now corrected the title.

**- Fig. 2: TFR and IRP levels follow the same pattern as loading controls. These WB need to be more convincing. Including quantification and statistical testing would help (as done for Fig. 2F)**.

**A:** We have remade some immunoblots and included added the respective quantifications (Figure 2C-E)

**- Fig 4D, E: It seems Fe increases basal and ATP-linked OCR (as has previously been shown, for instance in PMID 29484788) but does cachexia significantly impair OCR? The effect size seems to be very small and a test of significance needs to be provided.**

**A:** We repeated experiments using freshly prepared conditioned medium and found a significant impairment of OCR, in particular of basal respiration, maximal respiration and ATP linked OCR. (**updated Fig 4C-F**)

**- Fig. 5: it should be specified in the text when these effects (improved body weight, muscle area etc.) were measured - was it at the end of the experiment (i.e. end of survival curve) or at an earlier time point?**

**A**: Thank you for the remark, we added more details in the text to clarify. Except the survival curve, all the phenotypic measurements *in vivo* were performed on day 12 post C26-injection.

**- Fig. 6D and E are referred to wrongly in the text and in the figure legend**

**A**: Correction has been made.

**- Methods section, description of handgrip strength: Some text seems to be missing in the end of the paragraph**

A: We apologize for this mistake. Methods section has been corrected accordingly.

**- Figure legend of Fig 5, incorrect labelling for I-K ("(I-K) mRNA levels of Murf 1 (G), Atrogin 1 (H), and REDD1 (I)").**

**A**: Correction has been made.

**- Figure legend 6, incorrect description of statistical testing (or perhaps the wrong statistical test was used as student's t test cannot be used for multiple comparisons)**
**A**: Correction has been made.

**- Figure legend 7B should specify what the red dots stand for**
**A**: Correction has been made.

**Referee #3:**

**Wyart, Hsu, and co-authors present an investigation into the role of iron in cancer cachexia. The authors suggest that when taken together, their data indicate that in cancer cachexia, a lack of iron accumulation within mitochondria is a cause of muscle wasting. Furthermore, they suggest that iron supplementation may be an effective means to rescue muscle weakness in patients with cancer cachexia. Despite the fact that this manuscript explores a new area of cancer cachexia, there are issues that must be addressed prior to publication.**

**Major Concerns**

**1- There are a number of issues in the manuscript that raise technical concerns. For example, from the graphs presented, it appears that the variability of the number of samples in several sets of figure panels that appear to have resulted from the same animal experiment is quite high. For example, in Figures 1D, 1E, and 1F, the control samples appear to have an n of 8, 5, and 3, while the treated samples are 5, 6, and 4. Figure 6 and Supplemental Figure 2 are other examples. The authors should clarify how they selected samples for their experiments and if outliers were excluded, that information should be included in the methods.**

**A:** We apologize for this inconsistency. This results from the fact that muscle material is often limiting and our assays require different preparations (*e.g.*. aconitase assay requires fresh tissue, iron content quantification requires whole muscle). Otherwise, most phenotypic measurements (on strength and weight) were done systematically, hence the accumulated data. Concerning the experiment from FIG1D-F, the difference in numbers of replicate resulted from loss of samples due to shipment. We managed to retrieve part of the remaining materials from our collaborators and to perform the measurements again, thus increasing consistency. Hence, we decided to remove samples from FIG1D which were not suitable for transcriptional analysis.

**2- Additionally, the breaks in the axes of a significant number of graphs make actual differences between groups difficult to interpret – the authors should determine if these are absolutely necessary to their data presentation. Examples include Figures 1D, 1H, 1I, 1J, 1K, and 2A. Without a compelling reason, these graphs should be adjusted to reflect the actual differences between groups.**

**A**: We choose to represent all graphs of myotubes diameters with a break in the y-axis to better highlight the difference of diameter between controls and treated cells. Indeed, in our different conditions we generally observed a decrease in the diameter fiber ranging from 10 to 20%. While this decrease can seem moderate, it is actually very consistent and supported by statistical analysis. Moreover, a decrease lower than 30% generally results in the death of myotubes. We believe that a comparisons plotted in a full-length graph would be more difficult to appreciate. Therefore, we decided to keep the representation as previously displayed.

**3- In all myotube experiments, because myotubes continue to mature and increase in size after 4 days of differentiation, it is unclear to this reviewer if any of the effects**

**shown actually reflect atrophy of the myotubes. Without a time zero control for treatment, it is possible that treatment with conditioned media, siRNA, etc, did not simply prevent or allow continued differentiation and in fact actually induced or prevented atrophy. This point is particularly important because the authors assert that myogenesis is likely not the cause for improvement following iron treatment - but without doing these controls, these claims are not supported.**

**A:** We thank the Referee for this insightful remark. To address this issue, we performed as suggested an experiment measuring myotube size before and after treatment in order to compare with the respective baseline. We observed a steady increase in myotube size in the untreated condition (**R3-Fig1a circle)**, whereas C26-CM led to a significant decrease (**R3-Fig1a square**) that is remarkably counteracted by iron supplementation (**R3-Fig1a triangle**).
In addition, to elucidate the impact of myonuclear turnover, we measured fusion index in our settings and found no significant change following C26CM or iron treatment (**R3-Fig1b and updated Fig EV3A**).

Altogether, these data indicate that the effects of our *in vitro* treatments on myogenesis are negligible.

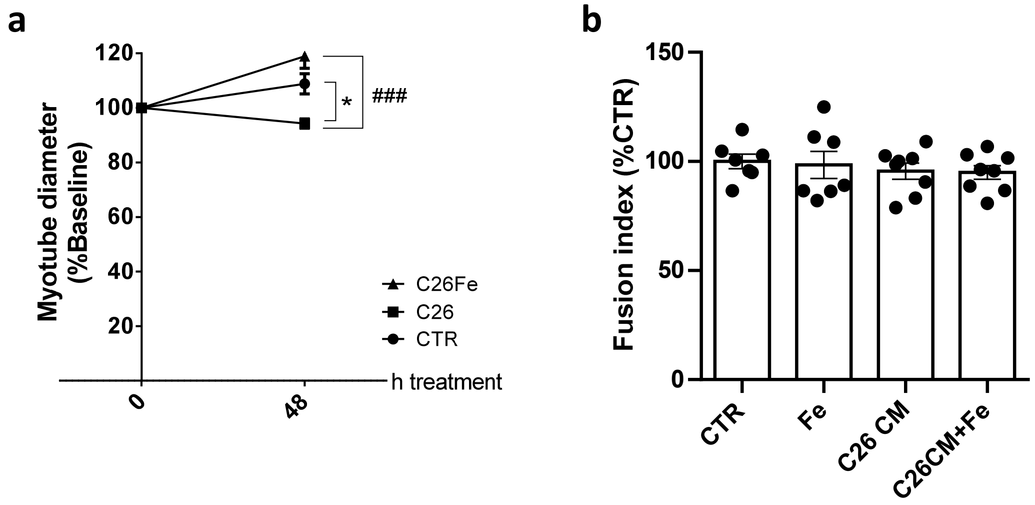


**R3- Fig.1: (a)** Myotube diameter normalized to day 0 values. **(b)** Fusion index of C2C12 myotubes after 48h treatment with Fe, C26CM or both. The fusion index was calculated as the ratio between the number of nuclei per myotubes and the total number of nuclei per field.

**In Figure 5E, it is unclear at what time point the tumor volumes were measured - are all of these mice that were euthanized at 12 days, or does the figure include mice from the survival study? If the latter is accurate, then the claims that tumor volume were not affected by FeCM are not accurate. To truly make this claim, the data represented in Figure 5E should only be from mice euthanized 12 days following tumor cell injection.**

**A:** We thank the referee for raising this point. We have added more precision in the corresponding text. Indeed, all mice were euthanized 12 days after tumor injection and tumors were weighted on the very same day. Survival experiments were performed in a second independent cohort.

**In Figure 6, it is unclear why no data from either FeCM or control mice were presented for panels A through E. These data would provide an important contextualization about changes that are occurring during C26 tumor-bearing , which cannot be determined without including these groups. As currently presented, we only have information about how FeCM treatment during tumor-bearing changed these variables.**

**A:** Besides mitochondrial iron quantification, we have now added in all the experiments a new set of samples that includes also controls and FeCM-treated healthy mice (**updated FIG 6**).

**In Supplementary Figure 4A, it is unclear why grip strength is normalized to a percentage of Day 10 values, although the manuscript text suggests that the data are from day 6. This is particularly confusing to this reviewer, as by my understanding, these mice would have had a FeCM injection 5 days after tumor cell implantation, and therefore the data as presented are the difference between either two doses or zero doses. An unnormalized presentation of the data would more clearly represent the results of supplementation.**

**A**: We have now corrected in the text. Mice were treated with iron on day 5 and day 10 after tumor cell implantation. Figure EV4A represents the grip strength measured on day 11 and normalized to day 10 to highlight the very rapid improvement of grip strength within 24h.

**It is unclear why the one non-cancer subject was left in the dataset for Figure 7, if goal is to make an argument that cancer patients benefit from iron supplementation, particularly because Supplemental Table 2 indicates that this person is otherwise healthy and significantly younger than all other included subjects. Lastly, the TSAT calculation should be included in the table, as it was a criterion used to determine iron deficiency.**
**Appropriate caveats of the human trial should also be noted somewhere in the manuscript - particularly the lack of a control group. If subjects were told that the treatment was going to improve their muscle strength, this amount of change isn't shocking**.

**A**: We included the transferrin saturation (TSAT) data and removed the non-cancer subject as suggested, data remained significant in the dominant hand. The present experiment was in the context of a clinical trial to correct anemia and given the preliminary nature of the study (and the patient condition), it was not possible to include a placebo. While it has not been explicitly mentioned to the patients that the treatment was meant to improve strength, further studies will be necessary to rule-out placebo effects (we addressed now this limitation in the discussion).

**Minor Concerns**

**In a number of figure legends, what ## or ### represents does not appear. For example, Supplementary Figure 3.**

**Figures 3H and 6D do not represent all data points as dot plots like all other graphs and should be updated. Also Figure 6 legend does not match the figure.**

**A row of Supplemental Table 2 appears to be mislabeled - two rows are labeled as non-dominant hand initial strength (kg).**

**In this reviewer's opinion, the supplemental movie does not add value to the manuscript.**

We corrected all the minor points as requested but prefer to keep the movie.

## 1st Revision - Editorial Decision 13th Jan 2022

Dear Dr. Porporato,

Thank you for submitting your revised manuscript. It has now been seen by all of the original referees.

The referees acknowledge that the study is significantly improved during revision. However, they also have some remaining concerns. In particular, referee #3 points out missing controls, recommends addition of the data provided as referee data to the manuscript and recommends toning down the conclusions regarding the patient data. Moreover, referee #2 finds that contradicting studies need to be discussed. We find that these are important concerns and they need to be addressed before publication.

Moreover, I need you to address the editorial points below before I can accept the manuscript.

• Please address the remaining referee concerns, provide a point-by-point response and mark the changes in the text (e.g. by leaving track changes on).
• We updated our journal's competing interests policy in January 2022 and request authors to consider both actual and perceived competing interests. Please review the policy https://www.embopress.org/competing-interests and update your competing interests if necessary.
• We note that Figure 6E blot was stripped and re-blotted. Please state this in the figure legend.
• Please provide 3-5 keywords for your study. These will be visible in the html version of the paper and on PubMed and will help increase the discoverability of your work.
• Please add "Disclosure statement and competing interests" and "Author Contributions" sections.
• Please rename "Methods" as "Materials and Methods".
• As per our format requirements, in the reference list, citations should be listed in alphabetical order and then chronologically, with the authors' surnames and initials inverted; where there are more than 10 authors on a paper, 10 will be listed, followed by 'et al.'. Please see https://www.embopress.org/page/journal/14693178/authorguide#referencesformat
• All funders mentioned in the manuscript submission system should be added to the Acknowledgements section.
• We note that the Appendix table is not called out in the text. Since the table is the only content of the Appendix file, it should be included in the manuscript.
• The movie needs to be ZIPped with its legends.
• Papers published in EMBO Reports include a 'synopsis' and 'bullet points' to further enhance discoverability. Both are displayed on the html version of the paper and are freely accessible to all readers. The synopsis includes a short standfirst summarizing the study in 1 or 2 sentences that summarize the paper and are provided by the authors and streamlined by the handling editor. I would therefore ask you to include your synopsis blurb and 3-5 bullet points listing the key experimental findings.
• In addition, please provide an image for the synopsis. This image should provide a rapid overview of the question addressed in the study but still needs to be kept fairly modest since the image size cannot exceed 550x400 pixels.

Thank you again for giving us to consider your manuscript for EMBO Reports, I look forward to your minor revision.

Kind regards,

Deniz Senyilmaz Tiebe

--
Deniz Senyilmaz Tiebe, PhD
Editor
EMBO Reports

Referee #1:

The revised manuscript is improved and all issues have been addressed.

Referee #2:

The revised version of this manuscript has added and clarified several critical aspects and is much improved. The authors have addressed most of my comments from the first revision. I feel, however, that the contradicting studies are still not adequately addressed / discussed, more care should be taken to address this. I believe that being more self-critical and careful in interpreting and comparing results actually adds to the scientific dialogue, and adds value, rather than making a paper weaker.

Referee #3:

The manuscript by Wyart and colleagues remains a novel concept for the field, and certainly adds to our understanding of cancer cachexia. The revised manuscript addresses many of my previous concerns and more clearly communicates the authors findings. However, I have three remaining concerns that I would like to express.

The authors provided reviewer data to respond to my concern about increases in myotube size over the 48 hours of treatment. However, these data has not been added to the manuscript. These data clearly show that approximately 75% of the difference in myotube size between the C26 conditioned media-treated myotubes and the C26 tumor media + iron myotubes is due to myotube growth in the iron group over the 48 hour treatment period. The C26 conditioned media treated myotubes appear to show a perhaps 5% atrophy over the 48 hours. This is much smaller than what is implied by the data in Figures 4G and 4H.

It would have also been nice to see an Fe control group in Figure 4D-4F, as it would have helped the reader to understand if iron simply shifts myotube metabolism. It would have also been interesting in the context of the iron-treated control mice which have now been added to Figure 6 - clearly iron alone affects whole muscle metabolism, as evidenced by changes in SDH activity. I find this to be particularly interesting, because the vast majority of mechanistic findings in C26 mice have failed in their translation to human patients, and therefore, a complete understanding of if a proposed therapeutic mechanism is specific to the C26 tumor or might apply to skeletal muscle in general is interesting.

While the authors have indeed included a caveat about the lack of a control group in the discussion of their iron supplementation data in patients, in my opinion, the language used in describing their results remains too strong. The authors state "these findings indicate that altered iron metabolism contributes to muscle weakness in cachectic patients." While possibly true, the lack of a control group means that they lack conclusive evidence, and "may contribute" is much more appropriate here. The assertion that improved strength was found in the non-dominant hand "for most patients" is also misleading, and it is difficult to see in Figure 7B if this is actually true. Furthermore, it is disappointing that the authors elected to remove the table of the patient data that they included in the first version of the manuscript - with the table, it was much easier to see if the claim of most patients having increased strength was true.

**Point-by-Point Response**

Dear Dr. Porporato,

Thank you for submitting your revised manuscript. It has now been seen by all of the original referees.

The referees acknowledge that the study is significantly improved during revision. However, they also have some remaining concerns. In particular, referee #3 points out missing controls, recommends addition of the data provided as referee data to the manuscript and recommends toning down the conclusions regarding the patient data. Moreover, referee #2 finds that contradicting studies need to be discussed. We find that these are important concerns and they need to be addressed before publication.

Moreover, I need you to address the editorial points below before I can accept the manuscript.

• Please address the remaining referee concerns, provide a point-by-point response and mark the changes in the text (e.g. by leaving track changes on).

**We replied to all the concerns, provided modified text (with track-changes) and updated the figures.**

• We updated our journal's competing interests policy in January 2022 and request authors to consider both actual and perceived competing interests. Please review the policy https://www.embopress.org/competing-interests and update your competing interests if necessary.

**We verified**

• We note that Figure 6E blot was stripped and re-blotted. Please state this in the figure legend.

**We stated in legends**

• Please provide 3-5 keywords for your study. These will be visible in the html version of the paper and on PubMed and will help increase the discoverability of your work.

**The keywords have been added.**

• Please add "Disclosure statement and competing interests" and "Author Contributions" sections.

**The two sections have been added.**

• Please rename "Methods" as "Materials and Methods".

**We have modified accordingly.**

• As per our format requirements, in the reference list, citations should be listed in alphabetical order and then chronologically, with the authors' surnames and initials inverted; where there are more than 10 authors on a paper, 10 will be listed, followed by 'et al.'. Please see https://www.embopress.org/page/journal/14693178/authorguide#referencesformat

**We have modified accordingly.**

• All funders mentioned in the manuscript submission system should be added to the Acknowledgements section.

**The funders have been added.**

• We note that the Appendix table is not called out in the text. Since the table is the only content of the Appendix file, it should be included in the manuscript.

**We have now included the appendix in the manuscript.**

• The movie needs to be ZIPped with its legends.

**DONE**

• Papers published in EMBO Reports include a 'synopsis' and 'bullet points' to further enhance discoverability. Both are displayed on the html version of the paper and are freely accessible to all readers. The synopsis includes a short standfirst summarizing the study in 1 or 2 sentences that summarize the paper and are provided by the authors and streamlined by the handling editor. I would therefore ask you to include your synopsis blurb and 3-5 bullet points listing the key experimental findings.

**We included**

• In addition, please provide an image for the synopsis. This image should provide a rapid overview of the question addressed in the study but still needs to be kept fairly modest since the image size cannot exceed 550x400 pixels.

**We would like to provide the following picture.**

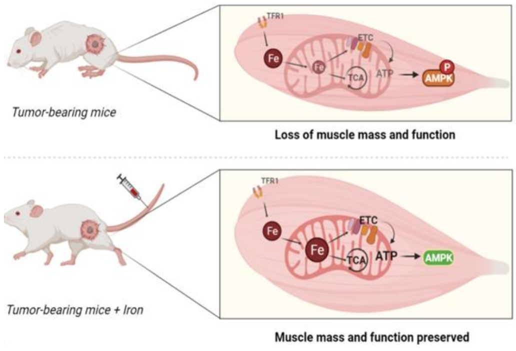

Referee #1:

The revised manuscript is improved and all issues have been addressed.

**Thank you**

Referee #2:

The revised version of this manuscript has added and clarified several critical aspects and is much improved. The authors have addressed most of my comments from the first revision. I feel, however, that the contradicting studies are still not adequately addressed / discussed, more care should be taken to address this. I believe that being more self-critical and careful in interpreting and comparing results actually adds to the scientific dialogue, and adds value, rather than making a paper weaker.

**We have now addressed more in detail the mentioned studies and added new ones to enlarge the scope of our discussion, addressing in a constructive way other studies characterizing muscle iron loading.**

Referee #3:

The manuscript by Wyart and colleagues remains a novel concept for the field, and certainly adds to our understanding of cancer cachexia. The revised manuscript addresses many of my previous concerns and more clearly communicates the authors findings. However, I have three remaining concerns that I would like to express.

The authors provided reviewer data to respond to my concern about increases in myotube size over the 48 hours of treatment. However, these data has not been added to the manuscript. These data clearly show that approximately 75% of the difference in myotube size between the C26 conditioned media-treated myotubes and the C26 tumor media + iron myotubes is due to myotube growth in the iron group over the 48 hour treatment period. The C26 conditioned media treated myotubes appear to show a perhaps 5% atrophy over the 48 hours. This is much smaller than what is implied by the data in Figures 4G and 4H.

**Indeed, the smaller difference compared to Figures 4G and 4H is due to the fact that all our data of myotube diameter were quantified at the experimental endpoint as commonly done in the field (for example: 28541289, 28928431, 26137861, 30894018), and normalized to control values (untreated group). In this new version, we have now added the corresponding graph in EV3 previously presented only in the point-by-point, as requested by the reviewer.**

It would have also been nice to see an Fe control group in Figure 4D-4F, as it would have helped the reader to understand if iron simply shifts myotube metabolism. It would have

also been interesting in the context of the iron-treated control mice which have now been added to Figure 6 - clearly iron alone affects whole muscle metabolism, as evidenced by changes in SDH activity. I find this to be particularly interesting, because the vast majority of mechanistic findings in C26 mice have failed in their translation to human patients, and therefore, a complete understanding of if a proposed therapeutic mechanism is specific to the C26 tumor or might apply to skeletal muscle in general is interesting.

**We have added the new graph as EV3A, showing that iron alone does not change the metabolic profile of myotubes.**

While the authors have indeed included a caveat about the lack of a control group in the discussion of their iron supplementation data in patients, in my opinion, the language used in describing their results remains too strong. The authors state "these findings indicate that altered iron metabolism contributes to muscle weakness in cachectic patients." While possibly true, the lack of a control group means that they lack conclusive evidence, and "may contribute" is much more appropriate here. The assertion that improved strength was found in the non-dominant hand "for most patients" is also misleading, and it is difficult to see in Figure 7B if this is actually true. Furthermore, it is disappointing that the authors elected to remove the table of the patient data that they included in the first version of the manuscript - with the table, it was much easier to see if the claim of most patients having increased strength was true.

**We have toned down our description about patients' data in the results and discussion based on reviewer's suggestion and added more precision about the observation of improved strength upon iron supplementation. The table was present in the previous version as appendix, it is now in the Extended Version of the manuscript.**

## 2nd Revision - Editorial Decision 25th Jan 2022

Dear Paolo,

Thank you for submitting your revised manuscript. I have now looked at everything and all is fine. Therefore, I am very pleased to accept your manuscript for publication in EMBO Reports.

Congratulations on a nice work!

Kind regards,

Deniz
--
Deniz Senyilmaz Tiebe, PhD
Editor
EMBO Reports

At the end of this email I include important information about how to proceed. Please ensure that you take the time to read the information and complete and return the necessary forms to allow us to publish your manuscript as quickly as possible.

As part of the EMBO publication's Transparent Editorial Process, EMBO reports publishes online a Review Process File to accompany accepted manuscripts. As you are aware, this File will be published in conjunction with your paper and will include the referee reports, your point-by-point response and all pertinent correspondence relating to the manuscript.

If you do NOT want this File to be published, please inform the editorial office within 2 days, if you have not done so already, otherwise the File will be published by default [contact: emboreports@embo.org]. If you do opt out, the Review Process File link will point to the following statement: "No Review Process File is available with this article, as the authors have chosen not to make the review process public in this case." Please note that the author checklist will still be published even if you opt out of the transparent process.

Thank you again for your contribution to EMBO reports and congratulations on a successful publication. Please consider us again in the future for your most exciting work.

***********************************************************************************

THINGS TO DO NOW:

You will receive proofs by e-mail approximately 2-3 weeks after all relevant files have been sent to our Production Office; you should return your corrections within 2 days of receiving the proofs.

Please inform us if there is likely to be any difficulty in reaching you at the above address at that time. Failure to meet our deadlines may result in a delay of publication, or publication without your corrections.

All further communications concerning your paper should quote reference number EMBOR-2021-53746V3 and be addressed to emboreports@wiley.com.

Should you be planning a Press Release on your article, please get in contact with emboreports@wiley.com as early as possible, in order to coordinate publication and release dates.

# EMBO PRESS

YOU MUST COMPLETE ALL CELLS WITH A PINK BACKGROUND ↓

PLEASE NOTE THAT THIS CHECKLIST WILL BE PUBLISHED ALONGSIDE YOUR PAPER

Corresponding Author Name: Paolo Ettore Porporato
Journal Submitted to: Embo Reports
Manuscript Number: EMBOR-2021-53746

## Reporting Checklist For Life Sciences Articles (Rev. June 2017)

This checklist is used to ensure good reporting standards and to improve the reproducibility of published results. These guidelines are consistent with the Principles and Guidelines for Reporting Preclinical Research issued by the NIH in 2014. Please follow the journal's authorship guidelines in preparing your manuscript.

### A- Figures
### 1. Data
### The data shown in figures should satisfy the following conditions:

- the data were obtained and processed according to the field's best practice and are presented to reflect the results of the experiments in an accurate and unbiased manner.
- figure panels include only data points, measurements or observations that can be compared to each other in a scientifically meaningful way.
- graphs include clearly labeled error bars for independent experiments and sample sizes. Unless justified, error bars should not be shown for technical replicates.
- if n< 5, the individual data points from each experiment should be plotted and any statistical test employed should be justified
- Source Data should be included to report the data underlying graphs. Please follow the guidelines set out in the author ship guidelines on Data Presentation.

### 2. Captions

### Each figure caption should contain the following information, for each panel where they are relevant:

- a specification of the experimental system investigated (eg cell line, species name).
- the assay(s) and method(s) used to carry out the reported observations and measurements
- an explicit mention of the biological and chemical entity(ies) that are being measured.
- an explicit mention of the biological and chemical entity(ies) that are altered/varied/perturbed in a controlled manner.

- the exact sample size (n) for each experimental group/condition, given as a number, not a range;
- a description of the sample collection allowing the reader to understand whether the samples represent technical or biological replicates (including how many animals, litters, cultures, etc.).
- a statement of how many times the experiment shown was independently replicated in the laboratory.
- definitions of statistical methods and measures:
  - common tests, such as t-test (please specify whether paired vs. unpaired), simple $\chi 2$ tests, Wilcoxon and Mann-Whitney tests, can be unambiguously identified by name only, but more complex techniques should be described in the methods section;
  - are tests one-sided or two-sided?
  - are there adjustments for multiple comparisons?
  - exact statistical test results, e.g., P values = x but not P values < x;
  - definition of 'center values' as median or average;
  - definition of error bars as s.d. or s.e.m.

Any descriptions too long for the figure legend should be included in the methods section and/or with the source data.

**In the pink boxes below, please ensure that the answers to the following questions are reported in the manuscript itself. Every question should be answered. If the question is not relevant to your research, please write NA (non applicable). We encourage you to include a specific subsection in the methods section for statistics, reagents, animal models and human subjects.**

### USEFUL LINKS FOR COMPLETING THIS FORM

http://www.antibodypedia.com
http://1degreebio.org
http://www.equator-network.org/reporting-guidelines/improving-bioscience-research-repor
http://grants.nih.gov/grants/olaw/olaw.htm
http://www.mrc.ac.uk/Ourresearch/Ethicsresearchguidance/Useofanimals/index.htm
http://ClinicalTrials.gov
http://www.consort-statement.org
http://www.consort-statement.org/checklists/view/32-consort/66-title
http://www.equator-network.org/reporting-guidelines/reporting-recommendations-for-tumo
http://datadryad.org
http://figshare.com
http://www.ncbi.nlm.nih.gov/gap
http://www.ebi.ac.uk/ega
http://biomodels.net/
http://biomodels.net/miriam/
http://jjj.biochem.sun.ac.za
https://osp.od.nih.gov/biosafety-biosecurity-and-emerging-biotechnology/
http://www.selectagents.gov/

### B- Statistics and general methods

Please fill out these boxes ↓ (Do not worry if you cannot see all your text once you press return)

| | |
|---|---|
| 1.a. How was the sample size chosen to ensure adequate power to detect a pre-specified effect size? | Sample size was based on sample sizes used in pregress literature and based on our experience. |
| 1.b. For animal studies, include a statement about sample size estimate even if no statistical methods were used. | Sample size was chosen analogous to sample sizes used in general in the field and based on our experience. |
| 2. Describe inclusion/exclusion criteria if samples or animals were excluded from the analysis. Were the criteria pre-established? | NA |
| 3. Were any steps taken to minimize the effects of subjective bias when allocating animals/samples to treatment (e.g. randomization procedure)? If yes, please describe. | Key experiments concerning myotube diameter analysis was performed in blind. |
| For animal studies, include a statement about randomization even if no randomization was used. | Animals were randomized according to initial body weight |
| 4.a. Were any steps taken to minimize the effects of subjective bias during group allocation or/and when assessing results (e.g. blinding of the investigator)? If yes please describe. | as mentioned before |
| 4.b. For animal studies, include a statement about blinding even if no blinding was done | No blinding were done in animal experiments |
| 5. For every figure, are statistical tests justified as appropriate? | Yes, statistical tests are reported in figure legends and justified as appropriate in Materials and Methods. |
| Do the data meet the assumptions of the tests (e.g., normal distribution)? Describe any methods used to assess it. | Yes |
| Is there an estimate of variation within each group of data? | Yes, estimates of variation within each data group are shown as SEM (for bars) or quantiles (for boxplots) or data points (for scatterplots). |

| | |
|---|---|
| Is the variance similar between the groups that are being statistically compared? | Yes |

### C- Reagents

| | |
|---|---|
| 6. To show that antibodies were profiled for use in the system under study (assay and species), provide a citation, catalog number and/or clone number, supplementary information or reference to an antibody validation profile. e.g., Antibodypedia (see link list at top right), 1DegreeBio (see link list at top right). | Catalog number and manifacturer are included in the manuscript for each antibody |
| 7. Identify the source of cell lines and report if they were recently authenticated (e.g., by STR profiling) and tested for mycoplasma contamination. | The source of cell lines are listed appropriately and tested for mycoplasma contamination. |

* for all hyperlinks, please see the table at the top right of the document

### D- Animal Models

| | |
|---|---|
| 8. Report species, strain, gender, age of animals and genetic modification status where applicable. Please detail housing and husbandry conditions and the source of animals. | Indicated in the manuscript |
| 9. For experiments involving live vertebrates, include a statement of compliance with ethical regulations and identify the committee(s) approving the experiments. | Yes |
| 10. We recommend consulting the ARRIVE guidelines (see link list at top right) (PLoS Biol. 8(6), e1000412, 2010) to ensure that other relevant aspects of animal studies are adequately reported. See author guidelines, under 'Reporting Guidelines'. See also: NIH (see link list at top right) and MRC (see link list at top right) recommendations. Please confirm compliance. | Yes |

### E- Human Subjects

| | |
|---|---|
| 11. Identify the committee(s) approving the study protocol. | Antwerp University Hospital ethical committee (#B300201941420) and Ethical Committee for Clinical Experimentation of Provincia di Padova (protocol number 3674/AO/15) |
| 12. Include a statement confirming that informed consent was obtained from all subjects and that the experiments conformed to the principles set out in the WMA Declaration of Helsinki and the Department of Health and Human Services Belmont Report. | All participants gave written informed consent to participate in the study and the study was approved by the Antwerp University Hospital ethical committee (handgrip strenght) and Ethical Committee for Clinical Experimentation of Provincia di Padova (biopsies) in accordance with the ethical standards established by the 1964 Declaration of Helsinki. |
| 13. For publication of patient photos, include a statement confirming that consent to publish was obtained. | NA |
| 14. Report any restrictions on the availability (and/or on the use) of human data or samples. | NA |
| 15. Report the clinical trial registration number (at ClinicalTrials.gov or equivalent), where applicable. | NA |
| 16. For phase II and III randomized controlled trials, please refer to the CONSORT flow diagram (see link list at top right) and submit the CONSORT checklist (see link list at top right) with your submission. See author guidelines, under 'Reporting Guidelines'. Please confirm you have submitted this list. | NA |
| 17. For tumor marker prognostic studies, we recommend that you follow the REMARK reporting guidelines (see link list at top right). See author guidelines, under 'Reporting Guidelines'. Please confirm you have followed these guidelines. | NA |

### F- Data Accessibility

| | |
|---|---|
| 18: Provide a "Data Availability" section at the end of the Materials & Methods, listing the accession codes for data generated in this study and deposited in a public database (e.g. RNA-Seq data: Gene Expression Omnibus GSE39462, Proteomics data: PRIDE PXD000208 etc.) Please refer to our author guidelines for 'Data Deposition'.<br><br>Data deposition in a public repository is mandatory for:<br>a. Protein, DNA and RNA sequences<br>b. Macromolecular structures<br>c. Crystallographic data for small molecules<br>d. Functional genomics data<br>e. Proteomics and molecular interactions | NA |
| 19. Deposition is strongly recommended for any datasets that are central and integral to the study; please consider the journal's data policy. If no structured public repository exists for a given data type, we encourage the provision of datasets in the manuscript as a Supplementary Document (see author guidelines under 'Expanded View' or in unstructured repositories such as Dryad (see link list at top right) or Figshare (see link list at top right). | NA |
| 20. Access to human clinical and genomic datasets should be provided with as few restrictions as possible while respecting ethical obligations to the patients and relevant medical and legal issues. If practically possible and compatible with the individual consent agreement used in the study, such data should be deposited in one of the major public access-controlled repositories such as dbGAP (see link list at top right) or EGA (see link list at top right). | NA |
| 21. Computational models that are central and integral to a study should be shared without restrictions and provided in a machine-readable form. The relevant accession numbers or links should be provided. When possible, standardized format (SBML, CellML) should be used instead of scripts (e.g. MATLAB). Authors are strongly encouraged to follow the MIRIAM guidelines (see link list at top right) and deposit their model in a public database such as Biomodels (see link list at top right) or JWS Online (see link list at top right). If computer source code is provided with the paper, it should be deposited in a public repository or included in supplementary information. | NA |

### G- Dual use research of concern

| | |
|---|---|
| 22. Could your study fall under dual use research restrictions? Please check biosecurity documents (see link list at top right) and list of select agents and toxins (APHIS/CDC) (see link list at top right). According to our biosecurity guidelines, provide a statement only if it could. | NA |
